# Distinctive characters of *Nostoc* genomes in cyanolichens

**DOI:** 10.1186/s12864-018-4743-5

**Published:** 2018-06-05

**Authors:** Andrey N. Gagunashvili, Ólafur S. Andrésson

**Affiliations:** Faculty of Life and Environmental Sciences, University of Iceland, Sturlugata 7, Reykjavík, 101 Iceland

**Keywords:** Cyanobacteria, *Nostoc*, Lichen, Symbiosis, Symbiotic competence

## Abstract

**Background:**

Cyanobacteria of the genus *Nostoc* are capable of forming symbioses with a wide range of organism, including a diverse assemblage of cyanolichens. Only certain lineages of *Nostoc* appear to be able to form a close, stable symbiosis, raising the question whether symbiotic competence is determined by specific sets of genes and functionalities.

**Results:**

We present the complete genome sequencing, annotation and analysis of two lichen *Nostoc* strains. Comparison with other *Nostoc* genomes allowed identification of genes potentially involved in symbioses with a broad range of partners including lichen mycobionts. The presence of additional genes necessary for symbiotic competence is likely reflected in larger genome sizes of symbiotic *Nostoc* strains. Some of the identified genes are presumably involved in the initial recognition and establishment of the symbiotic association, while others may confer advantage to cyanobionts during cohabitation with a mycobiont in the lichen symbiosis.

**Conclusions:**

Our study presents the first genome sequencing and genome-scale analysis of lichen-associated *Nostoc* strains. These data provide insight into the molecular nature of the cyanolichen symbiosis and pinpoint candidate genes for further studies aimed at deciphering the genetic mechanisms behind the symbiotic competence of *Nostoc*. Since many phylogenetic studies have shown that *Nostoc* is a polyphyletic group that includes several lineages, this work also provides an improved molecular basis for demarcation of a *Nostoc* clade with symbiotic competence.

**Electronic supplementary material:**

The online version of this article (10.1186/s12864-018-4743-5) contains supplementary material, which is available to authorized users.

## Background

Lichens are symbiotic associations between a fungus (mycobiont) and a photosynthetic partner (photobiont) that can be an eukaryotic alga (phycobiont), a cyanobacterium (cyanobiont), or both [1]. While the vast majority of lichen fungi (> 13,500 species), mainly from Ascomycota, associate with green algae (*Chlorophyta*), over 1500 species of lichen-forming fungi form so called “cyanolichens” that have cyanobacteria as primary photobionts (forming “bipartite” lichens) or accessory photobionts (forming “tripartite” lichens) [2]. Cyanobacterial symbioses have evolved repeatedly in different lineages of lichen-forming fungi [3–5], resulting in convergently similar thallus morphology in distantly related cyanolichens [2].

In lichen symbioses cyanobacteria provide mycobionts with photosynthate and/or fixed nitrogen. At the same time, the fungal partners provide the cyanobacteria with moisture, carbon dioxide and inorganic ions, as well as a relatively stable habitat, protected from environmental extremes and predation [1].

The association of fungal mycobiont partner and the photobiont partner (e.g. *Nostoc*) can either be by codispersal, e.g. in the lecanoromycete lichen *Lobaria pulmonaria* [6], or de novo by the pairing of a germinating spore and a free-living photobiont, as generally found in the lecanoromycete genus *Peltigera* [7, 8].

Most lichen symbioses are thought to be obligate as the majority of mycobionts are refractory to propagation in vitro and do not survive without their photosynthetic partners [9, 10]. And, although many cyanobacterial symbionts can be readily isolated and maintained in pure culture [11], they often appear unable to establish aposymbiotic populations outside lichen thalli in nature [12].

*Nostoc* is common in cyanolichens, especially in the temperate and cold regions of the world. All *Nostoc* species are filamentous and have complex life cycles involving cellular differentiation. Their non-branching filaments consist of cylindrical or spherical vegetative cells with intercalary heterocysts, large specialized nitrogen-fixing cells developing in mature trichomes [13]. The filaments of *Nostoc* strains are usually covered with a sheath of mucilage and many free-living *Nostoc* can form gelatinous macroscopic colonies in nature. The ability to produce mucilage and to form hormogonia, slender motile filaments, is generally used to distinguish *Nostoc* strains from the closely related genus *Anabaena* [13, 14], but they can be more reliably differentiated by akinete size and shape, together with other morphological characters [15]. However, some strains of *Nostoc* only produce hormogonia erratically or do not produce them at all [14, 16, 17].

The taxonomy of the family Nostocaceae is still rather poorly resolved, as exemplified by the placement of *Calothrix* and *Tolypothrix* spp. in several phylogenetic clades and the separation of *Nostoc* spp. into different clades [18]. Symbiotic *Nostoc* strains of many cyanolichens, including both bi- and tripartite species of *Peltigera*, have traditionally been called *Nostoc punctiforme*, but cyanobacterial strains resembling *Nostoc muscorum*, *Nostoc sphaericum* and *Nostoc linckia* have also been cultured from *Peltigera* species [19, 20].

In the lichen symbiosis cyanobacteria tend to undergo several morphological and structural changes [19–23], confounding phenotypic identification. Thus, molecular techniques offer a powerful addition for studying the diversity of these organisms, and for comparing lichen symbiotic strains as well as free-living cyanobacteria. During the past fifteen years the cyanobacterial symbionts of lichens have been the subject of many molecular investigations which have greatly increased our understanding of symbiont diversity. However, most of these studies have been based on a limited number of marker genes (e.g. 16S rDNA, *rbcLX*, *trnL*) and have mainly been focused on phylogenetic relationships of different strains [8, 24–30]. So far little is known about what defines symbiotic competence of cyanobacteria on the genome level.

Genome sequencing of *N*. *punctiforme* PCC 73102 [31], a model strain for cyanobacterial symbiosis with plants, together with transposon mutagenesis [32, 33] and insertion of antibiotic resistance cassettes [34] have identified a number of genes involved in the symbiosis [35]. Here we present the complete sequence and analysis of genomes from lichen-symbiotic *Nostoc* strains - one from the bipartite lichen *Peltigera membranacea* and one from the tripartite lichen *Lobaria pulmonaria*, - together with a discussion of genes which appear distinctive for symbiotic *Nostoc*. In addition we make use of draft genome data from three more *Nostoc* strains derived from *P. membranacea* and metagenome data from the lichens *P. membranacea* and *P. malacea*, as well as currently available whole genome data from members of the Nostocales.

## Results and discussion

### Genome properties

**Fig. 1:**
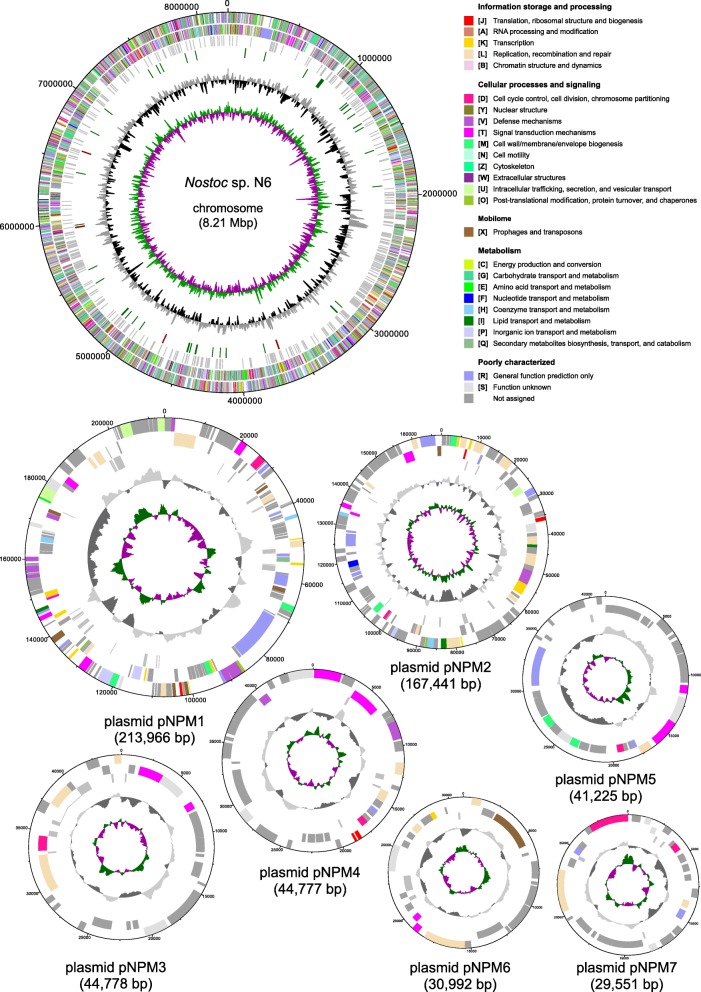
Chromosome and seven circular plasmids of the *Nostoc* sp. N6 genome. The outermost and second circles indicate genes in forward and reverse orientation color-coded by their COG categories. The third circles show pseudogenes. The fourth circle of the chromosome shows the rRNA genes (brown) and tRNA genes (green). The two innermost circles show GC content in gray and black and the GC skew in green (+) and purple (–)

**Fig. 2:**
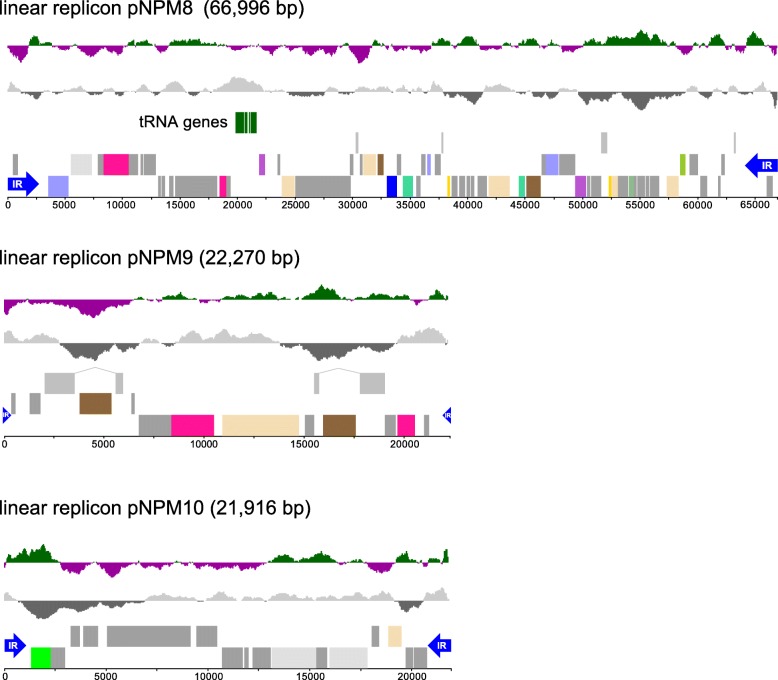
Linear replicons of *Nostoc* sp. N6. The lowermost and second lines indicate genes in forward and reverse orientation color-coded by their COG categories (see Figure 1). The third lines show pseudogenes. The two uppermost lines show GC content in gray and black and the GC skew in green (+) and purple (–). Blue arrows represent terminal inverted repeats (IR)

**Fig. 3:**
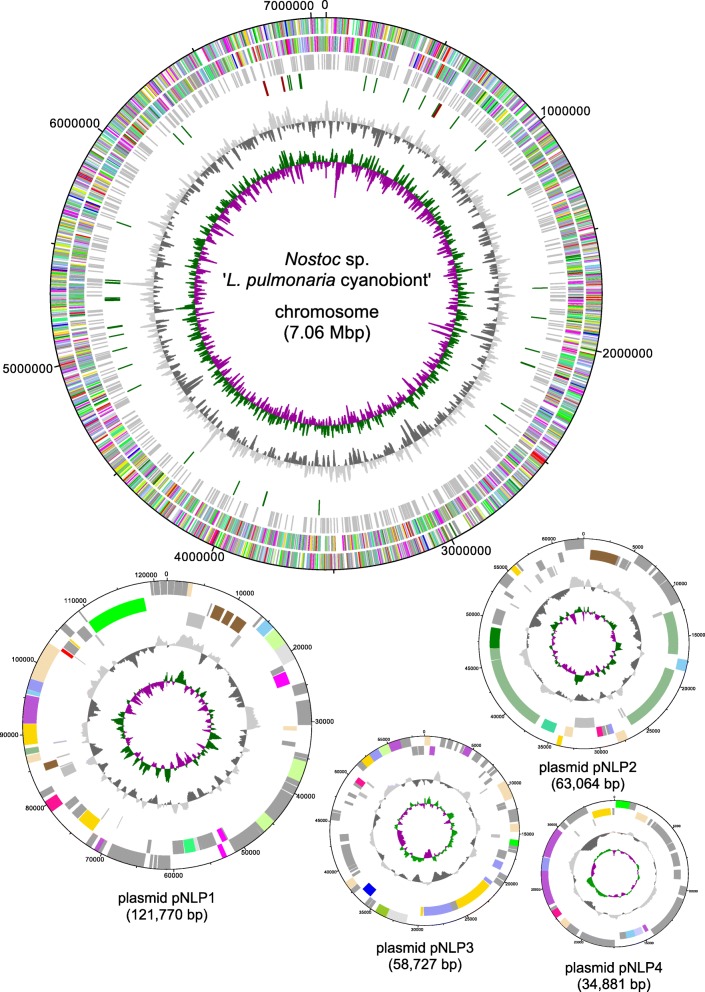
Chromosome and four plasmids of the *Nostoc* sp. ‘*L. pulmonaria* cyanobiont’ genome. The outermost and second circles indicate genes in forward and reverse orientation color-coded by their COG categories. The third circles show pseudogenes. The fourth circle of the chromosome shows the rRNA genes (brown) and tRNA genes (green). The two innermost circles show GC content in gray and black and the GC skew in green (+) and purple (–)

We have shotgun-sequenced DNA from five lichen-associated *Nostoc* strains and two lichens (Table 1). Draft genome assemblies were generated for three of the strains. The genomes of two strains, namely of the nosperin producer *Nostoc* sp. N6 [36] and the cyanobiont of *L. pulmonaria*, were completely assembled and annotated. The genome of *Nostoc* sp. N6 (8.9 Mb) is similar in size to that of symbiotic *N. punctiforme* PCC 73102 but it is larger than genomes of free-living *Nostoc* and *Anabaena* strains (Table 1 and Additional file 1). It consists of one circular chromosome (8.21 Mb) (Fig. 1) and 10 extrachromosomal replicons – 7 circular (pNPM1, 213,966 bp; pNPM2, 167,441 bp; pNPM3, 44,778 bp; pNPM4, 44,777 bp; pNPM5, 41,255 bp; pNPM6, 30,992 bp; pNPM7, 29,551 bp) (Fig. 1) and 3 linear (pNPM8, 66,996 bp; pNPM9, 22,270 bp; pNPM10, 21,916 bp) (Fig. 2). Based on the sequence coverage obtained, the linear replicons are present in higher copy numbers than the circular ones. pNPM9 and pNPM10 are characterized by a lower GC content (36.6% and 37.7%, respectively) than the rest of the genome (Table 2). The ends of pNPM8, pNPM9 and pNPM10 are in each case composed of identical or nearly identical inverted repeats 2.73, 0.16 and 1.06 kb long, respectively, with a conserved AAATTAACRGAC sequence at each end (Additional file 2: Figure S1). The ends of the linear plasmids form covalently closed hairpins, in which one DNA strand loops around and becomes the complementary strand. This was inferred from two observations: (i) presence of reads with palindrome sequences in the Nextera XT library and (ii) drop in coverage close to the ends of the plasmids and absence of read pairs spanning putative palindromes in the Nextera mate pair library. These methods use different DNA inputs for adapter addition: Nextera XT uses PCR to add adapters to denatured DNA whereas Nextera Mate Pair ligates adapters to blunt ends of double-stranded DNA.

**Table 1:** List of lichen-associated *Nostoc* strains sequenced in this study

Strain	Genome status	Genome size	DNA source
*Nostoc* sp. N6 ‘*Peltigera membranacea* cyanobiont’	Complete	8.9 Mb	Pure culture
*Nostoc* sp. 210A ‘*P. membranacea* cyanobiont’	Draft	8.33 Mb	Pure culture
*Nostoc* sp. 213 ‘*P. membranacea* cyanobiont’	Draft	8.33 Mb	Pure culture
*Nostoc* sp. 232 ‘*P. membranacea* cyanobiont’	Draft	9.16 Mb	Pure culture
*Nostoc* sp. ‘*Lobaria pulmonaria* cyanobiont’	Complete	7.34 Mb	Pure culture
*Nostoc* sp. ‘*P. malacea* cyanobiont’	Draft	8.52 Mb	Metagenomic

**Table 2:** Summary of the *Nostoc* sp. N6 and *Nostoc* sp. ‘*Lobaria pulmonaria* cyanobiont’ genomes

Replicon	GenBank accession	Topology	Size, bp	GC, %	CDS	Pseudogenes	Total	rRNA operons	tRNA genes	CRISPR arrays
*Nostoc* sp. N6										
chromosome	CP026681	circular	8,214,648	41.4	6205	518	6723	4	103	13
pNPM1	CP026682	circular	213,966	41.1	180	20	200	0	0	2
pNPM2	CP026684	circular	167,441	40.4	122	10	132	0	0	0
pNPM3	CP026685	circular	44,778	41.4	30	5	35	0	0	0
pNPM4	CP026686	circular	44,777	40.9	38	4	42	0	0	0
pNPM5	CP026687	circular	41,225	40.9	32	1	33	0	0	0
pNPM6	CP026688	circular	30,992	41.0	27	3	30	0	0	0
pNPM7	CP026689	circular	29,551	42.4	30	4	34	0	0	0
pNPM8	CP026690	linear	66,996	41.3	69	4	73	0	24	0
pNPM9	CP026691	linear	22,270	36.6	14	2	16	0	0	0
pNPM10	CP026683	linear	21,916	37.7	20	0	20	0	0	1
*Nostoc* sp. ‘*L. pulmonaria* cyanobiont’										
chromosome	CP026692	circular	7,061,466	41.6	5311	737	6048	3	74	7
pNLP1	CP026693	circular	121,770	40.3	82	9	91	0	0	0
pNLP2	CP026694	circular	63,064	42.2	36	10	46	0	0	0
pNLP3	CP026695	circular	58,727	42.0	60	4	64	0	0	0
pNLP4	CP026696	circular	34,881	42.4	33	2	35	0	0	0
Other genome features					*Nostoc* sp. N6			*Nostoc* sp. ‘*L. pulmonaria* cyanobiont’		
Intron in tRNA ^fMet^ gene					Yes			No		
Number of genes with inteins					5			0		
Number of excision elements in *nif* cluster					3			1		

Terminal inverted repeats and hairpins are common in linear DNA molecules enabling replication of genome ends [37]. Linear replicons are rarely found in Cyanobacteria, the only known examples being a 429.7 kb linear chromosome of *Cyanothece* sp. 51142 (accession NC_010547) [38] and a 37.15 kb incision element of *Anabaena variabilis* ATCC 29413 (accession NC_014000) [39]. An interesting feature of the largest linear plasmid pNPM8 is that it carries 24 tRNA genes out of the minimum of 32 tRNAs required for translation according to Crick’s wobble hypothesis [40]. Genes for tRNAs carrying isoleucine and histidine are not present, while there are 3 different tRNA genes for arginine and 2 for lysine, serine, glutamine and glutamic acid. tRNA genes have frequently been found in phages where they facilitate expression of phage genes with codons that are rare in the host genome [41, 42]. However, codon frequencies in the *Nostoc* sp. N6 chromosome vs. pNPM8 did not provide support for this (Additional file 2: Figure S2). The *Nostoc* sp. N6 linear elements might be phage remnants that have lost their structural proteins but retain the ability for self-replication in the host cells. Linear plasmid prophages are uncommon in nature, e.g. N15 in *E. coli* [43], PY54 in *Yersenia enterocolitica* [44] and *φ*KO2 in *Klebsiella oxytoca* [45]. Genes involved in chromosome partitioning and segregation, such as *parAB* [46] and *parM* [47], typical of many low copy number plasmids and bacterial chromosomes, were not found on the linear replicons, except for pNPM8 which carries a presumptive *parA* gene (NPM_80015). Interestingly, a gene encoding a typical phage protein, terminase, involved in DNA packaging into empty phage capsids, was found in pNPM9 (NPM_90012) disrupted by an insertion sequence.

The genome of the *L. pulmonaria* cyanobiont is nearly 1.5 Mb smaller than that of *Nostoc* sp. N6 and has a total size of 7.34 Mb. It consists of one circular chromosome (7.06 Mb) and 4 circular extrachromosomal replicons – pNLP1 (121,770 bp), pNLP2 (63,064 bp), pNLP3 (58,727 bp) and pNLP4 (34,881 bp) (Fig. 3). Compared to *Nostoc* sp. N6, the *L. pulmonaria* cyanobiont genome contains a smaller number of coding regions, a larger number of pseudogenes and 3 ribosomal DNA (rDNA) operons instead of the 4 copies generally found in Nostocales [48]. These features along with much slower growth observed in pure culture (3–4 times slower than *Nostoc* sp. N6) suggest genome shrinkage, gene loss and a possible semi-obligate nature of the cyanobiont. Interestingly, *cbiM* transporter genes, involved in the uptake of cobalt for cobalamin (vitamin B12) biosynthesis (locus tags NLP_0266 and NLP_2774), were found to be pseudo in the *L. pulmonaria* cyanobiont. Several other essential genes had disabling mutations but had intact functional homologues.

Inversion of the GC skew ((G −C)/(G+C)) from positive to negative, typically seen at the replication origin of bacterial chromosomes, cannot be applied to predict the location of *oriC* in Cyanobacteria since their DNA asymmetry is greatly disturbed by mutational pressure [49] and extensive chromosome rearrangements (see below). A putative *oriC* for the chromosomes was identified in both strains downstream of *dnaA*, encoding a chromosomal replication initiation protein (locus tags NPM_0001 and NLP_0001) (Additional file 2: Figure S3). Both *oriC* regions contain 6 DnaA boxes, most with TTTTCCACA, the DnaA box motif specific for Cyanobacteria [50]. Location of *oriC* adjacent to the *dnaN* gene encoding the *β* subunit of DNA polymerase III has been claimed to be universal among Cyanobacteria [50, 51]. In free-living *Nostoc* strains and in *Anabaena variabilis*, *oriC* is located in the intergenic region between the *dnaA* and *dnaN* genes. However, in lichen-associated *Nostoc* strains and in *N. punctiforme*, *dnaA* and *dnaN* genes are not adjacent (∼ 52 kb apart in *N. punctiforme*). Interestingly, no apparent DnaA boxes were found adjacent to either the *dnaA* gene (Npun_F0001) or the *dnaN* gene (Npun_F0034) in *N. punctiforme* whereas a putative *oriC* with a cluster of DnaA boxes lies within the Npun_F0036–Npun_F0037 intergenic region.

### Genome and proteome comparison

**Fig. 4:**
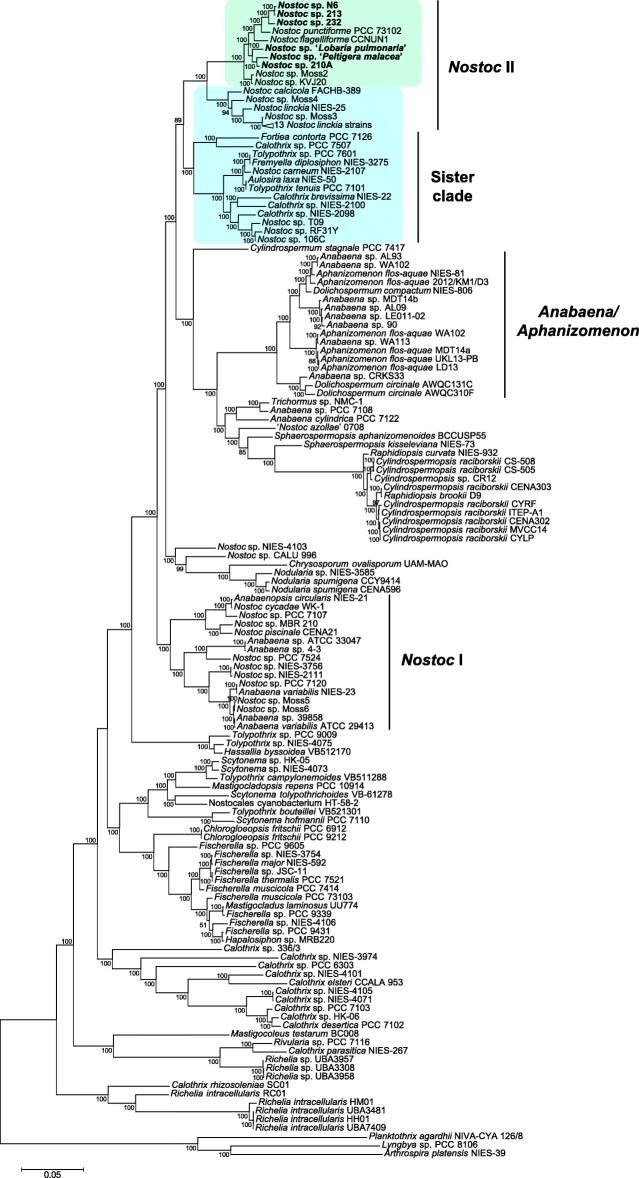
Maximum liklelihood phylogenomic tree of Nostocales strains based on 31 single-copy core bacterial phylogenetic markers [135]. *Arthrospira platensis* NIES-39, Lyngbya sp. PCC 8106 and *Planktothrix agardhii* NIVA-CYA 126/8 from the order Oscillatoriales were used as the outgroup. Numbers at branch nodes are bootstrap percentages based on 100 replicates (only values >50 are shown). Scale bar indicates 5% sequence divergence. Selected clades are named according to [29]. Predominantly symbiotic clade is highlighted with green, paraphyletic group is highlighted with blue. Lichen-associated strains are shown in bold

Phylogenetic analysis of 31 conserved single copy protein genes from Nostocales strains available in GenBank and JGI-IMG showed that lichen-associated *Nostoc* strains and *N. punctiforme* group together in a clade (*Nostoc* II; Fig. 4) suggesting a monophyletic origin of these symbiotic *Nostoc* strains. The recently sequenced symbiotic strains *Nostoc* sp. KVJ20 [52] and *Nostoc* sp. Moss 2 [30] also associate with this clade, whereas two other moss derived isolates together with terrestrial soil isolates of *Nostoccalcicola* and *Nostoclinckia* form a subclade within the *Nostoc* II clade. The free-living aquatic *Nostoc* strains group together with some *Anabaena* strains (clade *Nostoc* I; Fig. 4) while other *Anabaena* strains group with members of the genera *Aphanizomenon* and *Dolichospermum* (clade *Anabaena*/*Aphanizomenon*). These major phylogenetic relationships are in accord with what has been found by O’Brien and coworkers [29] and by Warshan [30]. Although O’Brien’s clade *Nostoc* II contains three free-living terrestrial isolates – *N. punctiforme* SAG 71.79, *N. commune* 02011101 and *N. muscorum* SAG 57.79 (currently known as *Desmonostoc muscorum*) – none of them have been tested for symbiotic competence and two of the strains have *P. membranacea* cyanobionts as their closest phylogenetic relatives. *Nostoc* strains with specificity for symbiosis with *Gunnera* also fall within clade II [53] but their genome sequences are currently not available.

Large scale genome comparisons of lichen-associated strains with *N. punctiforme* PCC 73102 reveal a low level of synteny between them (Additional file 2: Figure S4) indicating high genome plasticity and genome shuffling in these strains (Additional file 2: Figure S5). The ten most prominent regions of synteny include the whole set of genes involved in nitrogen fixation (the *nif* gene cluster; locally collinear block 1), some photosynthetic genes (locally collinear block 3), and genes encoding the majority of ribosomal proteins (Clusters of Orthologous Groups (COG) category J; locally collinear block 7) (Additional file 2: Figure S5, Additional file 3), which are known to be syntenic across species [54, 55], as well as many genes involved in carbohydrate transport and metabolism (G) and cell wall/membrane/envelope biogenesis (M).

**Fig. 5:**
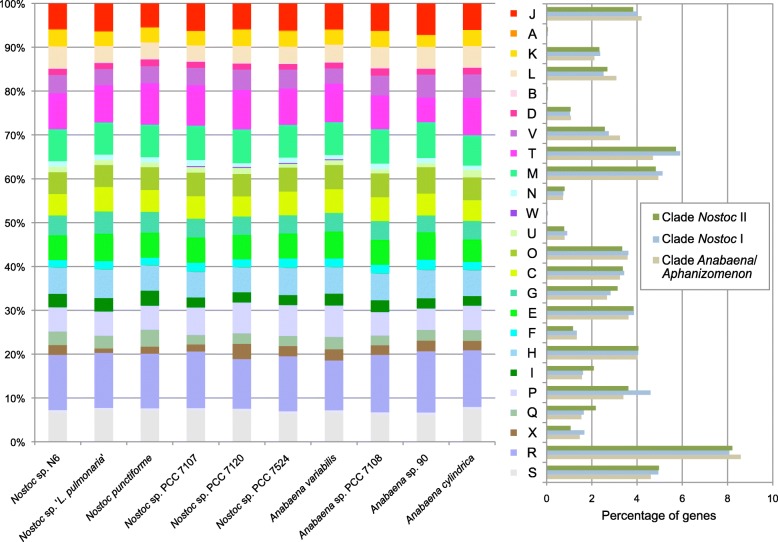
COG category distribution of the proteins encoded in the genomes of selected *Nostoc* and *Anabaena* strains. The ordinate axes indicate the percentage of genes in each COG functional category relative to the genes of all COG categories (left) and percentage COG category distribution among different clades (right)

Although symbiotic *Nostoc* strains N6 and *N. punctiforme* show a higher number of encoded proteins in most COG categories than the free-living strains (mean total 4344 vs. 3587; Additional file 2: Table S5), the fraction assigned to COG categories was similar (63-68%) and the distribution among categories was similar for all analyzed *Nostoc* and *Anabaena* strains (Fig. 5). On the average, clade II of symbiotic *Nostoc* strains has a higher proportion of genes devoted to carbohydrate transport and metabolism (G), lipid transport and metabolism (I) and secondary metabolite biosynthesis, transport and catabolism (Q) compared to clade I *Nostoc* and *Anabaena* strains. Interestingly, clade I, comprised of free-living *Nostoc* and *Anabaena* strains (Additional file 1), exhibits the highest number of genes for inorganic ion transport and metabolism (P) (Fig. 5). In contrast, the *Nostoc* strains in symbiosis may benefit from host (plant or fungus) provision of inorganic ions, e.g. by the action of mycobiont siderophores [56].

The genome of *Nostoc* sp. N6 was found to encode the highest number of COG category L proteins (DNA replication, recombination and repair) (Additional file 2: Table S3). One possible explanation for this is that terrestrial cyanobacteria are generally subject to ultraviolet (UV) irradiation, and therefore are expected to possess efficient mechanisms for repair of UV-induced DNA damage [57]. Nevertheless, one of the genes involved in biosynthesis of the cyanobacterial sunscreen scytonemin (tyrosinase, *tyrP*) [58–60] is missing in the *Nostoc* sp. N6 genome, and another one (DSBA oxidoreductase, *frnE*) was found to be a pseudogene due to an in-frame stop codon (Additional file 2: Table S6). Both genes are thought to participate in oxidative dimerization of precursors to form scytonemin [61]. The cyanobionts of *L. pulmonaria* and *P. malacea* appear to have all genes necessary for scytonemin biosynthesis (Additional file 2: Table S6). It is possible that *Nostoc* sp. N6 compensates for the lack of scytonemin with a larger repertoire of enzymes for DNA repair.

*Nostoc* sp. N6 has a high number of transposable elements and inteins (Additional file 2: Tables S7-S9). The best studied case of inteins in Cyanobacteria is in DnaE (the *α* subunit of DNA polymerase III) encoded by two different ORFs and assembled by trans-splicing [62]. More information on transposons and inteins can be found in Supporting Information.

The majority of lichen associated *Nostoc* strains studied appears to have an alternative vanadium-based nitrogenase in addition to the standard molybdenum-based nitrogenase. This includes three of the strains studied here, *Nostoc* spp. 210A, 213 and 232, as well as the *P. malacea* lichen cyanobiont [63]. The reason for the common occurrence of this alternative nitrogenase in lichen-associated cyanobacteria is not clear, but may relate to low availability of molybdenum in cyanolichens and/or a functional advantage at relatively low growth temperatures [64]. A novel finding is that these lichen *Nostoc* strains carry a near complete duplication of VnfD, with a cyanobacterial aminoacyl-tRNA synthetase domain (CAAD; pfam14159) inserted at the carboxy end, similar to peptide insertions found in GluRS, ValRS, LeuRS and IleRS amino acid tRNA synthetases in a variety of cyanobacteria, where this domain is thought to direct the proteins to thylakoid membranes, a key source of reducing power and ATP [65]. Further information on the molybdenum-based (*nif*) and the vanadium-based gene clusters (*vnf*) is provided in Supporting Information (Additional file 2: Figures S9 and S10).

### Comparison to minimal bacterial and cyanobacterial gene sets

In order to see what pathways might differ, be incomplete or deteriorating in lichen cyanobionts, we performed comparative analyses with the minimal bacterial [66] and the cyanobacterial “core” and “shell” [67] gene sets, represented by 206 and 682 genes respectively (Additional files 4 and 5). The most prominent differences were observed for pyrimidine metabolism, in split ribonucleotide reductase enzymes, carbohydrate catabolism and potassium transport, as described in the Supporting Information.

### Identification of genes specific to symbiotic *Nostoc* strains

To identify functions enriched in symbiotic *Nostoc* genomes (present in over 80% of group), we performed an all-by-all BLASTP search of all the proteomes from the *Nostoc* I and II clades plus the sister clade (Fig. 4) and assigned identified hits into orthologous groups. For the lichen-associated *Nostoc* strains 152 protein orthologs satisfied the criteria set (see Methods), 189 orthologs for the predominantly symbiotic clade, 399 for the *Nostoc* II clade and 385 for the combined *Nostoc* II clade and sister clade (see Additional file 6 for listing). A few of the most prominent gene collectives associated with symbiotic *Nostoc* are discussed below.

**Hormogonium regulating locus.**

Hormogonia are relatively short motile filaments, lacking heterocysts, formed by cyanobacteria from the orders Nostocales and Stigonematales. A hormogonium-inducing factor (HIF) secreted by plant hosts induces symbiotic cyanobacteria to differentiate hormogonia and they then dedifferentiate back into nitrogen-fixing filaments after about 48 h [68]. The capacity of *Nostoc* strains to form hormogonia has been found to be necessary, but not singularly sufficient, for symbiotic competence [69, 70]. An aqueous extract of the hosting hornwort *Anthoceros punctatus* appears to contain a hormogonium repressing factor (HRF) because it suppresses HIF-induced hormogonia formation. Analysis of*N. punctiforme* mutants led to proposal of the following model of HRF-dependent modulation of HrmR transcriptional regulation [71]: HRF enters the *Nostoc* cell and it, or a derivative similar to galacturonate, binds to the repressor protein HrmR, decreasing affinity for the *hrmR* and *hrmE* promoter regions. This derepresses transcription of these genes, somehow leading to inhibition of hormogonia formation and return to the vegetative state [72].

**Fig. 6:**
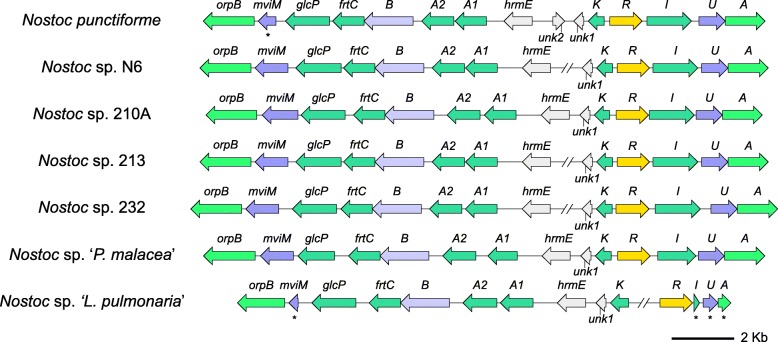
Hormogonium regulating and sugar transporter loci in symbiotic *Nostoc* strains. Pseudogenes are denoted with an asterisk. *orpB*, carbohydrate-selective porin; *mviM*, inositol-2-dehydrogenase; *glpC*, glucose permease; *frtA1A2BC*, ABC-type fructose transporters; *hrmE*, inositol oxygenase; *hrmK*, gluconate kinase; *hrmR*, LacI family transcriptional regulator; *hrmI*, glucuronate isomerase; *hrmU*, D-mannonate oxidoreductase; *hrmA* and *unk*, unknown. A broken genome line indicates 2 separate loci

In *N. punctiforme* the hormogonium regulating locus is linked to genes involved in sugar transport (Fig. 6) [73]. It has been hypothesized that these genes are involved in HRF-induced synthesis of a metabolite inhibitor of hormogonium differentiation, rather than a carbon catabolic function [72]. This metabolite, probably similar to galacturonate [72], binding to the HrmR protein, may act in a positive feedback loop alleviating repression of the *hrm* locus, leading to increased production of the metabolite and at the same time facilitating increased import of sugars such as glucose, fructose and sucrose. Since PfkA (6-phosphofructokinase) appears to be nonessential in symbiotic *Nostoc*, these sugars must be channeled through the oxidative pentose phosphate (OPP) pathway or the Entner-Doudoroff (ED) pathway, both producing NADPH reducing equivalents facilitating biosynthesis and decreasing dependence on the non-oxidative pentose phosphate reactions (Calvin cycle). This catabolic shift may simultaneously induce development from hormogonia to vegetative cells and heterocysts. The shift from vegetative cells to heterocysts is accompanied by an increase of the OPP-specific Gnd and an even greater increase in Zwf [74], indicating increased carbon flow via the ED pathway. The *hrm* locus is restricted to the *Nostoc* II clade and its sister clade.

**D-alanine-D-alanine ligase operon.** In addition to a conventional cell-wall specific D-Ala-D-Ala ligase (DdlA), the lichen associated *Nostoc* strains uniquely harbour another D-Ala-D-Ala ligase, of type 3, thought to be involved in modification of peptide moieties in peptidoglycans as described in Supporting Information (Additional file 2: Figures S14 and S15).

**Phosphonate biosynthetic genes.**

**Fig. 7:**
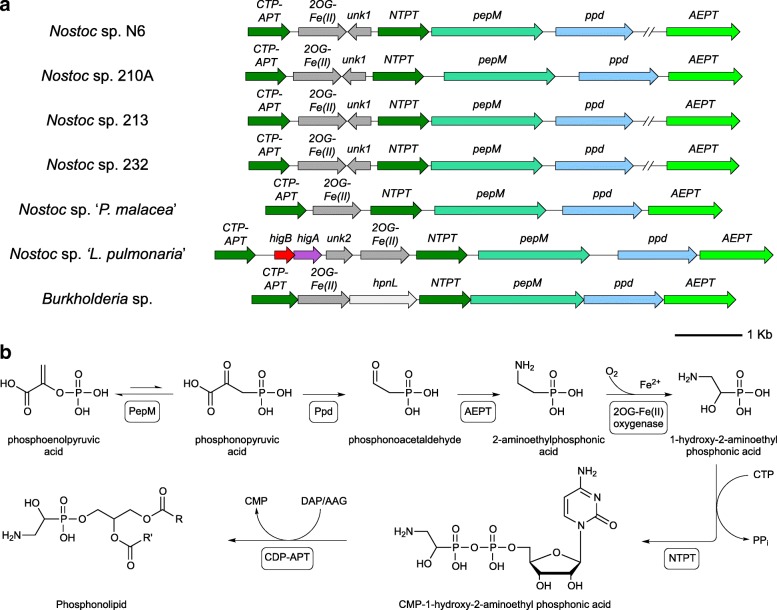
Phosphonate biosynthetic gene clusters of lichen cyanobionts (**a**) and proposed encoded biosynthetic pathway (**b**) (adapted from [75]). A homologous gene cluster from *Burkholderia* is shown for comparison. CTP-APT, CDP-alcohol phosphatidyltransferase; OG-Fe(II), 2-oxoglutarate non-heme Fe(II) dependent oxidase; *unk*, conserved hypothetical proteins; NTPT, NTP transferase; *pepM*, phosphoenolpyruvate phosphomutase; *ppd*, phosphonopyruvate decarboxylase; AEPT, 2-aminoethylphosphonate aminotransferase; *hpnL*, putative membrane protein; *higBA*, toxin-antitoxin module. A broken genome line indicates separate loci

Phosphonates are organophosphorus compounds containing direct carbon-phosphorus bonds, e.g. in phosphonolipids where they can not be cleaved by regular phospholipases. The biochemical pathways and gene clusters for phosphonolipid synthesis are well studied [75], facilitating recognition in new settings as in the case of the lichen-associated *Nostoc* strains in this study (Fig. 7). This cluster is characteristic of the *Nostoc* II clade. Extended information is provided in the Supporting Information.

The additional peptidoglycan and phosphonate lipid functions may lead to cell wall modifications that are well tailored to the intrathalline environment, as well as being recognized as compatible by a mycobiont during establishment of symbiosis. Despite being sheltered by a mycobiont, lichen cyanobionts are subjected to extracellular enzymes and metabolites produced by both the mycobiont and intrathalline bacteria. Therefore, a specific ability to withstand some unfavorable aspects of this cohabitation is expected from lichen associated *Nostoc* strains.

**Chloramphenicol phosphotransferase.** Chloramphenicol is an antibiotic produced by *Streptomyces venezuelae* ATCC 10712 and several other actinomycetes [76]. The bacteriostatic activity of chloramphenicol results from its binding to the 50S subunit of the bacterial ribosome blocking peptidyl transferase [77]. *S. venezuelae* escapes the toxicity of its own lethal secondary metabolite by expressing a chloramphenicol phosphotransferase (CPT) that phosphorylates the primary (C-3) hydroxyl of chloramphenicol (Additional file 2: Figure S16) [78]. Genes encoding CPT were found almost exclusively in the *Nostoc* II clade.

**Gas vesicles, sulfur metabolism.** Genes encoding gas vesicle proteins have been shown to be involved in hormogonium function and establishment of the *N*. *punctiforme* symbiosis [79, 80] as well as in the symbiosis of *Nostoc* with feathermoss [81]. Gas vesicle proteins GvpC, GvpV and GvpW appear to be characteristic for the *Nostoc* II clade and its sister clade. Several genes associated with assimilation of alkane sulfonates in the moss-*Nostoc* association [82] were also found to be enriched in the *Nostoc* II clade.

**Sensory mechanisms.** All the comparison groups were found to have differences related to sensory mechanisms and motility, including signal transduction histidine kinases, methyl-accepting proteins as well as diguanylate cyclases, thought to be involved in regulating motility in cyanobacteria [83]. The diversity and rapid divergence of sensory mechanisms underlines the great variety of ecotypes found in the genus *Nostoc*, especially in strains with symbiotic capacity [84]. Differences in genes involved in sensory mechanisms were also found in the comparison made by Warshan et al. [82].

### Secondary metabolites

Cyanobacteria produce a multitude of secondary metabolites, many of them toxic [85, 86]. In a recent study, Liaimer et al. [52] found that *Nostoc* symbionts of the liverwort *Blasia pusilla* more frequently produce nodularin and microcystin type compounds antagonistic to other *Nostoc* strains than free living *Nostoc* from the same locality. Most types of secondary compounds were detected in only 1 to 4 out of the 20 strains examined. The occurrence of the main secondary metabolite pathways in *Nostoc punctiforme*, in the *Nostoc* strains from the *Blasia* habitats [52] and in the lichen-derived strains of the present study shows little overlap. One of the secondary compounds detected by Liaimer et al. [52] is the polyketide synthase plus non-ribosomal peptide synthase (PKS-NRPS) product nosperin [36]. We previously suggested that nosperin might have cytotoxic properties analogous to cyanobiont microcystins [87, 88] which can serve as protective compounds in cyanolichens, e.g. against grazers. Interestingly, *Nostoc* sp. 232 was found to be devoid of *nsp* genes encoding nosperin, but it has a putative microcystin gene cluster not found in the *nsp* containing *Nostoc* sp. N6 strain. Similarly, the single *Blasia*-habitat *Nostoc* strain showing nosperin does not exhibit any of the other metabolites under study [52]. Remnants of the *nsp* gene cluster were found on the chromosome of the *L. pulmonaria* cyanobiont (Additional file 2: Figure S19), where almost the entire cluster has been deleted, probably due to the absence of selective pressure.

Whole genome sequencing of the nosperin producer *Nostoc* sp. N6 revealed that the *nsp* gene cluster is located on the chromosome. The abundance of insertion sequences surrounding the cluster and the apparent mixed gene origin suggests that it has been acquired as a genomic island through horizontal transfer and undergone several intragenomic recombination events [36]. The genome of *Nostoc* sp. N6 was also found to encode pathways for the biosynthesis of nostopeptolide- [89] and banyaside/suomilide-like [52] compounds as well as nostocyclopeptide [90] (Additional file 2: Table S14). Nostopeptolide in *Nostoc punctiforme* has been found to be a major hormogonium-repressing factor and is therefore considered responsible for cellular differentiation of *Nostoc* [91].

Nostocyclopeptides are cyclic heptapeptides with a unique imino linkage in the macrocyclic ring, isolated from the lichen cyanobiont *Nostoc* sp. ATCC 53789 [92]. Two homologous NRPS functions (locus tags NPM_1843 and NPM_1844) were found in the genome of *Nostoc* sp. N6. A nostoclide-like compound with a very similar structure, cyanobacterin, produced by the cyanobacterium *Tolypothrix* sp. PCC 9009 (*Scytonema hofmanni* UTEX 2349) [93, 94], was found to inhibit the growth of many cyanobacteria, as well as green algae and angiosperms [95, 96]. Based on the homology with *Tolypothrix* sp. PCC 9009, we identified putative gene clusters for biosynthesis of nostoclide-like compounds in the genomes of *Nostoc* spp. 210A and 232 (Additional file 2: Figure S20b). More extensive information on secondary products can be found in the Supporting Information.

## Conclusions

The complete genome sequences and comparative genomic analyses of two lichen-associated *Nostoc* strains are presented here. The finished genomes, manually curated, are appropriate for all types of detailed analyses and act as high-quality references for comparative purposes [97]. Comparative genome analysis of symbiotic and free-living cyanobacteria allowed the identification of several pathways that may contribute to symbiotic competence of *Nostoc* strains. One pathway, encoded by the hormogonium regulating (*hrm*) locus, was previously identified in symbiotically competent *N. punctiforme* and plays a central role in abrogating hormogonia formation. This pathway is similar to pathways of sugar uronate metabolism in heterotrophic non-cyanobacterial prokaryotes [71, 72]. Although the *hrm* locus has been shown to be important in the *Nostoc*-plant symbiosis, its presence in all of the lichen-associated *Nostoc* strains from this study suggests it is also relevant to establishing *Nostoc*-mycobiont symbioses. Pathways that may be involved in cell wall biogenesis of lichen cyanobionts were also identified, including novel gene clusters encoding synthesis of phosphonate lipids and an MXAN_4097-like amidoligase (D-Ala-D-Ala ligase).

It is apparent that the ability to form and maintain symbiosis is a complex trait governed by many factors and different combinations of these factors may result in different symbiotic associations – from loose to the most intimate. The study presented here is the first attempt to determine, on a whole genome level, what genes and features may contribute to symbiotic competence of *Nostoc* cyanobionts in lichens. Although we have pinpointed candidate symbiotic genes in the lichen-associated *Nostoc* genomes, a more thorough analysis, e.g. with targeted mutations and resynthesis of symbiosis, is required to verify the importance and involvement of individual genes and pathways. Some progress has been achieved in studying plant-cyanobacterial symbioses using the readily cultured hornwort *Anthoceros* and the liverwort *Blasia* as model organisms. However, there are substantial differences between plant- and mycobiont-cyanobacterial symbioses, e.g. due to the heterotrophic nature of fungi. In contrast to many lichens with green algal photobionts, the bionts of cyanolichens are difficult to culture and synthesize in the laboratory. Problems include slow growth or unculturability of most mycobionts, difficulties in obtaining axenic cultures of photobionts, and in maintaining resynthesized biont cultures for long periods of time. Few attempts have been documented of cyanolichen resynthesis under laboratory conditions [98–103] and currently there are no available models to study mycobiont-*Nostoc* symbiosis. The use of the glomeromycete *Geosiphon pyriforme*, which is easily culturable and capable of forming symbiosis with *Nostoc* strains, can help to overcome some of these limitations [104].

Recent studies of ten genomes and proteomes from moss-associated *Nostoc* strains compared to the non-symbiotic *Nostoc* sp. CALU 996, identified a number of gene families present in the symbiotic strains but not in the comparison strain [81], [82]. Several of these, including the *hrm* locus, genes encoding gas vesicle proteins, genes connected with sulfur metabolism and genes linked to sensory mechanisms were identical or similar to symbiotic-specific gene clusters identified in the lichen-associated *Nostoc*.

In addition to *Nostoc*, several other nostocean cyanobacteria have been reported in lichen symbioses. Members of the genera *Scytonema*, *Calothrix*, *Dichothrix*, and *Tolypothrix* have also been found in lichens as cyanobionts [20, 105, 106]. Isolation and genome sequencing of these lichen-associated strains can add more support and knowledge to our current understanding of what determines symbiotic competence in *Nostoc* and other cyanobacteria.

## Methods

### Isolation and culture of *Nostoc* strains

*Peltigera membranacea* thalli for cyanobiont isolation were collected from a moss carpet (*Hylocomium splendens* and *Pleurozium schreiberi*) at Keldur, Reykjavik, Iceland, and *Lobaria pulmonaria* thallus was collected from a maple tree trunk (*Acer macrophyllum*) at Cedar Road, Vancouver Island, British Columbia, Canada. *Nostoc* strains were isolated on BG-11_0_ agar medium as previously described [107], purified by repeated streaking on the same medium and maintained at room temperature.

### DNA extraction, library construction and sequencing

Genomic DNA was prepared from *Nostoc* cultures grown in liquid BG-11_0_ medium at an illumination of 50 *μ*mol photons m ^−2^ s ^−1^ as described in [108]. Sequencing libraries were prepared using Nextera XT and, for some strains, Nextera Mate Pair Sample Preparation Kits (Illumina) according to the manufacturer’s protocols and sequenced using MiSeq Reagent Kits v2 with 2 ×250 and 2 ×150 cycles, respectively (Additional file 2: Table S1). Roche 454 reads of *P. membranacea* and *P. malacea* metagenomes generated previously [109] were also used in this study to increase the number of lichen-associated *Nostoc* strains.

### Genome assembly

Draft assemblies of *Nostoc* spp. N6 and ‘*Lobaria pulmonaria* cyanobiont’ genomes were constructed using MIRA v3.2.1 (www.chevreux.org/projects_mira.html) and further processed and verified using GAP5 (Staden package) [110] (Additional file 2: Table S1). Remaining gaps were closed by PCR and Sanger sequencing. Draft genomes of *Nostoc* spp. 210A, 213, 232 and the *P. malacea* metagenome were assembled using SPAdes v3.10.1 [111] with default parameters. Prior to assembly Illumina reads were processed with Trimmomatic v0.36 [112] with “LEADING:20 TRAILING:20 SLIDINGWINDOW:4:15 MINLEN:20” parameters. SPAdes contigs >1 kb were binned using MaxBin v2.2.4 [113] and those belonging to Cyanobacteria were scaffolded using BESST v2.2.6 [114, 115]. The resulting assemblies were improved with FinishM v0.0.9 (https://github.com/wwood/finishm) and Pilon v2.11.6 [116]. Scaffolds were taxonomically classified using Kaiju (http://kaiju.binf.ku.dk/) [117] and PhyloPythiaS+ (http://phylopythias.bifo.helmholtz-hzi.de/) [118] web servers. Those not assigned to Cyanobacteria were manually checked using a BLAST search [119], and contaminating scaffolds were removed. Completeness and contamination of the assemblies were assessed with CheckM v1.0.7 [120] (Additional file 2: Table S2).

### Genome annotation

Draft genome assemblies were annotated using the NCBI Prokaryotic Genome Annotation Pipeline [121]. For complete genomes ORFs were predicted with Prodigal [122], followed by manual correction in Artemis [123] using the gene prediction improvement pipeline GenePRIMP [124]. All encoded proteins were assigned functions by combining results from InterProScan [125], CDD [126] and BLAST searches [119] against the NCBI nonredundant (nr) database. Transfer RNA genes were identified with tRNAScan-SE-1.23 [127] and ribosomal RNA genes (5S, 16S, 23S) were predicted using RNAmmer [128]. Other non-coding RNAs were identified with Infernal (v.1.1) [129] using RFAM convariance models (http://ftp.ebi.ac.uk/pub/databases/Rfam). Identification of CRISPR elements was performed using CRISPRfinder [130] and PILER-CR [131]. Pseudogenes were annotated using the GenePRIMP pipeline and rechecked manually in Artemis. Single in-frame stop codons and frameshifts were confirmed in the original assemblies. Ribosomal slippage was annotated according to standard operating procedures (SOP) at the GenePRIMP website (http://studylib.net/doc/7260119). Finally, short ORFs (encoding < 100 aa) without any significant homology (E-value >10^−2^) to the nr database, and ORFs represented solely by low-complexity sequences (e.g. spanning micro- and minisatellite regions) were removed from the annotation. Intein-containing proteins were identified by the presence of an intein/homing endonuclease domain (COG1372). Excision of *nifD* and *fdxN* excision elements in *Nostoc* sp. N6 was confirmed by previously generated RNA-Seq data [36] mapped with Bowtie 2 [132]. Origins of replication (*oriC*) were identified by locating DnaA boxes (TT ^A^/ _T_TNCACA) [133]. The location of a cluster of DnaA boxes, especially adjacent to *dnaA* and/or *dnaN* genes, is considered an indicator for the location of *oriC*. Transposases were classified into IS families using ISfinder (https://www-is.biotoul.fr/; [134]).

### Phylogenomic analysis

Available genomes of Nostocales strains along with *Arthrospira platensis* NIES-39, *Lyngbya* sp. PCC 8106 and *Planktothrix agardhii* NIVA-CYA 126/8 (order Oscillatoriales) as an outgroup were retrieved from GenBank and the Joint Genome Institute’s Integrated Microbial Genomes database (JGI-IMG) in January 2018. Thirty-one marker proteins that are universally conserved across the bacterial domain (*dnaG*, *frr*, *infC*, *nusA*, *pgk*, *pyrG*, *rplA*, *rplB*, *rplC*, *rplD*, *rplE*, *rplF*, *rplK*, *rplL*, *rplM*, *rplN*, *rplP*, *rplS*, *rplT*, *rpmA*, *rpoB*, *rpsB*, *rpsC*, *rpsE*, *rpsI*, *rpsJ*, *rpsK*, *rpsM*, *rpsS*, *smpB* and *tsf*) were extracted from the genomes using the AMPHORA2 pipeline [135] and aligned with MUSCLE [136]. An alignment mask was generated using Zorro [137]. The marker alignments were further concatenated into a single partitioned alignment and the best protein substitution model for each of the markers was predicted using the *concat_align.pl* script of phylogenomics-tools (https://github.com/kbseah/phylogenomics-tools; 10.5281/zenodo.46122). A maximum-likelihood phylogeny was derived using the PROTCATWAG model for tree search in RAxML v8.2.4 [138] automated by the *tree_calculations.pl* script of phylogenomics-tools. Branch support was assessed using the approximate likelihood ratio test for branches (SH-like aLRT) [139] with 100 replicates.

### Genome and proteome comparison

Whole genome comparisons were performed using PROmer (MUMmer 3.0 package; [140]) and Mauve [141]. To identify orthologous groups specific to different clades (Fig. 4) an all-by-all BLASTP search was performed on proteomes of 56 strains belonging to a) *Nostoc* I clade (16 strains), b) *Nostoc* II clade (27 strains), c) sister clade to *Nostoc* II clade (13 strains) with soft masking and thresholds: E-value < 10^−10^, percentage identity $\geqslant $50% and percentage match $\geqslant $50%. The resulting hits were clustered into orthologous groups using OrthoMCL [142, 143]. Orthologous groups specific to different clades were extracted as shown in Additional file 2: Figure S21. For COG category distribution comparison proteins encoded in the genomes of selected *Nostoc* and *Anabaena* strains were classified into COG functional categories [144] using RPS-BLAST against PSSMs (Position-Specific Scoring Matrices) from the updated COG database [145] with an E-value < 10^−2^ and the top hit retained.

## Additional files

Additional file 1 Nostocales strains used in this study. (XLS 44 kb)

Additional file 2 Supporting Information (SI) Appendix. (PDF 8156 kb)

Additional file 3 Proteins encoded in 10 most prominent locally collinear blocks in *Nostoc punctiforme* PCC 73102, *Nostoc* sp. N6 and Nostoc sp. ‘*Lobaria pulmonaria* cyanobiont’, identified by Mauve. (XLS 84 kb)

Additional file 4 Comparison of the minimal bacterial gene set to *Nostoc* sp. N6 and *Nostoc* sp. ‘*Lobaria pulmonaria* cyanobiont’. (XLS 61 kb)

Additional file 5 Comparison of the cyanobacterial core and shell gene set to *Nostoc* sp. N6 and *Nostoc* sp. ‘*Lobaria pulmonaria* cyanobiont’. (XLS 172 kb)

Additional file 6 Orthologous groups enriched in different clades. (XLS 756 kb)

## Abbreviations

AAG: Alkyl-acylglycerol
ATP: Adenosine triphosphate
CAAD: Cyanobacterial Aminoacyl-tRNA synthetase domain
CDP: Cytidine Diphosphate
CDP-APT: CDP-alcohol Phosphatidyltransferase
CMP: Cytidine monophosphate
COG: Clusters of Orthologous group
CPT: Chloramphenicol Phosphotransferase
CTP: Cytidine triphosphate
DAG: Ddiacylglycerol
dCMP: Deoxycytidine monophosphate
dCTP: Deoxycytidine triphosphate
dNTP: Nucleoside triphosphate
dTTP: Thymidine triphosphate
dUMP: Deoxyuridine monophosphate
dUTP: Uridine diphosphate
ED: Entner–Doudoroff
FAD: Flavin adenine dinucleotide
Gnd: 6-Gluconophosphate dehydrogenase
HAEP: 1-hydroxy-2-aminoethylphosphonate
HIF: Hormogonium inducing factor
HRF: Hormogonium repressing factor
IS: Insertion sequence
KEGG: Kyoto encyclopedia of genes and genomes
LAM: Lysine 2,3-Aminomutase
NADP: Nicotinamide adenine dinucleotide phosphate
NNI: Nearest neighbor interchange
NRPS: Non-ribosomal peptide synthetase
OPP: Oxidative pentose pathway
ORF: Open reading frame
PEP: Phosphoenolpyruvate
PepM: Phosphoenolpyruvate mutase
PKS: Polyketide synthase
Ppd: Phosphonopyruvate decarboxylase
PSSM: Position-specific scoring matrix
RNR: Ribonucleoside reductase
SET: Lysine Methyltransferase
UDP: Uridine diphosphate
UV: Ultraviolet
Zwf: Glucose-6-phosphate 1-Dehydrogenase

## References

[1] Nash TH (2008). Lichen Biology.

[2] Rikkinen J, Rai AK (2002). Cyanolichens: an evolutionary overview. Cyanobacteria in Symbiosis.

[3] Gargas A, DePriest PT, Grube M, Tehler A (1995). Multiple origins of lichen symbioses in fungi suggested by ssu rdna phylogeny. Science.

[4] Aptroot A (1998). Aspects of the integration of the taxonomy of lichenized and non-lichenized pyrenocarpous ascomycetes. The Lichenologist.

[5] Lutzoni F, Pagel M, Reeb V (2001). Major fungal lineages are derived from lichen symbiotic ancestors. Nature.

[6] Dal Grande F, Widmer I, Wagner HH, Scheidegger C (2012). Vertical and horizontal photobiont transmission within populations of a lichen symbiosis. Molecular Ecology.

[7] O’Brien HE, Miadlikowska J, Lutzoni F (2009). Assessing reproductive isolation in highly diverse communities of the lichen-forming fungal genus *Peltigera*. Evolution.

[8] O’Brien HE, Miadlikowska J, Lutzoni F (2013). Assessing population structure and host specialization in lichenized cyanobacteria. New Phytologist.

[9] Crittenden P, David J, Hawksworth D, Campbell F (1995). Attempted isolation and success in the culturing of a broad spectrum of lichen-forming and lichenicolous fungi. New Phytologist.

[10] Stocker-Wörgötter E, Hager A, Nash TH (2008). Culture methods for lichens and lichen symbionts. Lichen Biology.

[11] Ahmadjian V (1993). The Lichen Symbiosis.

[12] Rikkinen J (2013). Molecular studies on cyanobacterial diversity in lichen symbioses. MycoKeys.

[13] Waterbury J. B, Dworkin M, Falkow S (2006). The cyanobacteria—isolation, purification and identification. The Prokaryotes: Vol. 4: Bacteria: Firmicutes, Cyanobacteria.

[14] Rippka R, Deruelles J, Waterbury JB, Herdman M, Stanier RY (1979). Generic assignments, strain histories and properties of pure cultures of cyanobacteria. J Gen Microbiol.

[15] Rajaniemi P, Hrouzek P, Kaštovska K, Willame R, Rantala A, Hoffmann L, Komárek J, Sivonen K (2005). Phylogenetic and morphological evaluation of the genera *Anabaena*, *Aphanizomenon*, *Trichormus* and *Nostoc* (nostocales, cyanobacteria). Int J Syst Evol Microbiol.

[16] Lachance M-A (1981). Genetic relatedness of heterocystous cyanobacteria by deoxyribonucleic acid-deoxyribonucleic acid reassociation. Int J Syst Bacteriol.

[17] Rippka R (1988). Recognition and identification of cyanobacteria. Methods Enzymol.

[18] Dvořák P, Casamatta DA, Hašler P, Jahodářová E, Norwich AR, Poulíčková A (2017). Diversity of the cyanobacteria. Modern Topics in the Phototrophic Prokaryotes.

[19] Bergman B, Hällbom L (1982). *Nostoc* of *Peltigera canina* when lichenized and isolated. Can J Botany.

[20] Tschermak-Woess E, Galun M (1988). The algal partner. CRC Handbook of Lichenology.

[21] Koriem A, Ahmadjian V (1986). An ultrastructural-study of lichenized and cultured nostoc photobionts of *Peltigera canina*, *Peltigera rufescens*, and *Peltigera spuria*. Endocytobiosis and Cell Research.

[22] Boissière M-C (1987). Ultrastructural evidence for polyglucosidic reserves in *Nostoc* cells in *Peltigera* and *Collema* and the effect of thallus hydratation. Bibliotheca Lichenologica.

[23] Bergman B, Rai AN, Rasmussen U. Cyanobaterial associations In: Elmerich C, William Edward Newton WE, editors. Associative and Endophytic Nitrogen-fixing Bacteria and Cyanobacterial Associations. Springer: 2007. p. 257–301.

[24] Paulsrud P, Rikkinen J, Lindblad P (2000). Spatial patterns of photobiont diversity in some *Nostoc*-containing lichens. New Phytologist.

[25] Paulsrud P, Rikkinen J, Lindblad P (1998). Cyanobiont specificity in some *Nostoc*-containing lichens and in a *Peltigera aphthosa* photosymbiodeme. New Phytologist.

[26] Paulsrud P, Lindblad P (1998). Sequence variation of the trnaleu intron as a marker for genetic diversity and specificity of symbiotic cyanobacteria in some lichens. Appl Environ Microbiol.

[27] Rikkinen J, Oksanen I, Lohtander K (2002). Lichen guilds share related cyanobacterial symbionts. Science.

[28] Lohtander K, Oksanen I, Rikkinen J (2003). Genetic diversity of green algal and cyanobacterial photobionts in *Nephroma* (Peltigerales). Lichenologist.

[29] O’Brien H, Miadlikowska J, Lutzoni F (2005). Assessing host specialization in symbiotic cyanobacteria associated with four closely related species of the lichen fungus *Peltigera*. European J Phycology.

[30] Warshan D. Cyanobacteria in symbiosis with boreal forest feathermosses: from genome evolution and gene regulation to impact on the ecosystem. PhD thesis. 2017.

[31] Meeks JC. The genome of the filamentous cyanobacterium *Nostoc punctiforme*, what can we learn from it about free-living and symbiotic nitrogen fixation? In: Palacios R, Newton WE, editors. Genomes and Genomics of Nitrogen-fixing Organisms. Nitrogen Fixation: Origins, Applications, and Research Progress. Springer: 2005. p. 27–70.

[32] Cohen MF, Wallis JG, Campbell EL, Meeks JC (1994). Transposon mutagenesis of *Nostoc* sp. strain ATCC 29133, a filamentous cyanobacterium with multiple cellular differentiation alternatives. Microbiology.

[33] Cohen MF, Meeks JC, Cai YA, Wolk CP (1998). Transposon mutagenesis of heterocyst-forming filamentous cyanobacteria. Methods Enzymol.

[34] Cai Y, Wolk C. P (1990). Use of a conditionally lethal gene in *Anabaena* sp. strain pcc 7120 to select for double recombinants and to entrap insertion sequences. J Bacteriol.

[35] Adams D. G, Duggan P. S (2008). Cyanobacteria–bryophyte symbioses. J Exp Botany.

[36] Kampa A, Gagunashvili AN, Gulder TAM, Morinaka BI, Daolio C, Godejohann M, Miao VPW, Piel J, Andresson OS (2013). Metagenomic natural product discovery in lichen provides evidence for a family of biosynthetic pathways in diverse symbioses. Proc Nat Acad Sci USA.

[37] Meinhardt F, Schaffrath R, Larsen M (1997). Microbial linear plasmids. Appl Microbiol Biotechnol.

[38] Welsh EA, Liberton M, Stoeckel J, Loh T, Elvitigala T, Wang C, Wollam A, Fulton RS, Clifton SW, Jacobs JM, Aurora R, Ghosh BK, Sherman LA, Smith RD, Wilson RK, Pakrasi HB (2008). The genome of *Cyanothece* 51142, a unicellular diazotrophic cyanobacterium important in the marine nitrogen cycle. Proc Natl Acad Sci USA.

[39] Thiel T, Pratte BS, Zhong J, Goodwin L, Copeland A, Lucas S, Han C, Pitluck S, Land ML, Kyrpides NC, Woyke T (2014). Complete genome sequence of *Anabaena variabilis* ATCC 29413. Standards Genomic Sci.

[40] Crick F (1965). Codon-anticodon pairing: the wobble hypothesis. J Mole Biol.

[41] Bailly-Bechet M, Vergassola M, Rocha E (2007). Causes for the intriguing presence of tRNAs in phages. Genome Research.

[42] Enav H, Beja O, Mandel-Gutfreund Y (2012). Cyanophage tRNAs may have a role in cross-infectivity of oceanic *Prochlorococcus* and *Synechococcus* hosts. ISME J.

[43] Ravin V, Ravin N, Casjens S, Ford M, Hatfull G, Hendrix R (2000). Genomic sequence and analysis of the atypical temperate bacteriophage N15. J Mol Biol.

[44] Hertwig S, Klein I, Lurz R, Lanka E, Appel B (2003). PY54, a linear plasmid prophage of *Yersinia enterocolitica* with covalently closed ends. Molecular Microbiology.

[45] Casjens S, Gilcrease E, Huang W, Bunny K, Pedulla M, Ford M, Houtz J, Hatfull G, Hendrix R (2004). The pKO2 linear plasmid prophage of *Klebsiella oxytoca*. J Bacteriol.

[46] Bignell C, Thomas C (2001). The bacterial ParA-ParB partitioning proteins. J Biotechnol.

[47] Moller-Jensen J, Borch J, Dam M, Jensen R, Roepstorff P, Gerdes K (2003). Bacterial mitosis: ParM of plasmid R1 moves plasmid DNA by an actin-like insertional polymerization mechanism. Molecular Cell.

[48] Schirrmeister BE, Dalquen DA, Anisimova M, Bagheri H. C. Gene copy number variation and its significance in cyanobacterial phylogeny. BMC Microbiology. 2012; 12. 10.1186/1471-2180-12-17. 10.1186/1471-2180-12-177 PMC3552681 22894826

[49] Mackiewicz P, Zakrzewska-Czerwinska J, Zawilak A, Dudek M, Cebrat S (2004). Where does bacterial replication start? Rules for predicting the *oriC* region. Nucleic Acids Res.

[50] Gao F, Zhang C-T (2008). Origins of replication in *Cyanothece* 51142. Proc Natl Acad Sci USA.

[51] Zhou Y, Chen W-L, Wang L, Zhang C-C (2011). Identification of the *oriC* region and its influence on heterocyst development in the filamentous cyanobacterium *Anabaena* sp. strain PCC 71 20. Microbiology.

[52] Liaimer A, Jensen JB, Dittmann E (2016). A genetic and chemical perspective on symbiotic recruitment of cyanobacteria of the genus *Nostoc* into the host plant *Blasia pusilla* L. Front Microbiol.

[53] Svenning MM, Eriksson T, Rasmussen U (2005). Phylogeny of symbiotic cyanobacteria within the genus *Nostoc* based on 16S rDNA sequence analyses. Arch Microbiol.

[54] Ran L, Larsson J, Vigil-Stenman T, Nylander JAA, Ininbergs K, Zheng W-W, Lapidus A, Lowry S, Haselkorn R, Bergman B. Genome erosion in a nitrogen-fixing vertically transmitted endosymbiotic multicellular cyanobacterium. PLOS ONE. 2010; 5(7). 10.1371/journal.pone.001148. 10.1371/journal.pone.0011486 PMC2900214 20628610

[55] Wang H, Sivonen K, Rouhiainen L, Fewer DP, Lyra C, Rantala-Ylinen A, Vestola J, Jokela J, Rantasarkka K, Li Z, Liu B. Genome-derived insights into the biology of the hepatotoxic bloom-forming cyanobacterium *Anabaena* sp. strain 90. BMC Genomics. 2012; 13. 10.1186/1471-2164-13-61. 10.1186/1471-2164-13-613 PMC3542288 23148582

[56] Haselwandter K, Winkelmann G, Varma A, Chincholkar SB (2007). Siderophores of symbiotic fungi. Microbial Siderophores.

[57] Singh SP, Häder D-P, Sinha RP (2010). Cyanobacteria and ultraviolet radiation (uvr) stress: mitigation strategies. Ageing Res Rev.

[58] Soule T, Stout V, Swingley WD, Meeks JC, Garcia-Pichel F (2007). Molecular genetics and genomic analysis of scytonemin biosynthesis in *Nostoc punctiforme* ATCC 29133. J Bacteriol.

[59] Soule T, Garcia-Pichel F, Stout V (2009). Gene expression patterns associated with the biosynthesis of the sunscreen scytonemin in *Nostoc punctiforme* ATCC 29133 in response to UVA radiation. J Bacteriol.

[60] Soule T, Palmer K, Gao Q, Potrafka RM, Stout V, Garcia-Pichel F. A comparative genomics approach to understanding the biosynthesis of the sunscreen scytonemin in cyanobacteria. BMC Genomics. 2009a; 10(336). 10.1186/1471-2164-10-33. 10.1186/1471-2164-10-336 PMC2726228 19630972

[61] Malla S, Sommer MOA (2014). A sustainable route to produce the scytonemin precursor using *Escherichia coli*. Green Chemistry.

[62] Gogarten J, Senejani A, Zhaxybayeva O, Olendzenski L, Hilario E (2002). Inteins: structure, function, and evolution. Annu Rev Microbiol.

[63] Hodkinson BP, Allen JL, Forrest LL, Goffinet B, Sérusiaux E, Andrésson ÓS, Miao V, Bellenger J-P, Lutzoni F (2014). Lichen-symbiotic cyanobacteria associated with *Peltigera* have an alternative vanadium-dependent nitrogen fixation system. European J Phycol.

[64] Miller R, Eady R (1988). Molybdenum and vanadium nitrogenases of *Azotobacter chroococcum*, low temperature favours n _2_ reduction by vanadium nitrogenase. Biochem J.

[65] Luque I, Olmedo-Verd E, Santamaría-Gómez J, de Alda JAO, de Pouplana LR (2011). Membrane anchoring of aminoacyl-trna synthetases by convergent acquisition of a novel protein domain. J Biol Chem.

[66] Gil R, Silva F, Pereto J, Moya A (2004). Determination of the core of a minimal bacterial gene set. Microbiol Mole Biol Rev.

[67] Shi T, Falkowski PG. Genome evolution in cyanobacteria: the stable core and the variable shell, Vol. 105; 2008. pp. 2510–5. 10.1073/pnas.071116510. 10.1073/pnas.0711165105 PMC2268167 18268351

[68] Meeks JC, Elhai J (2002). Regulation of cellular differentiation in filamentous cyanobacteria in free-living and plant-associated symbiotic growth states. Microbiol Mole Biol Rev.

[69] Enderlin C, Meeks J (1983). Pure culture and reconstitution of the *Anthoceros-Nostoc* symbiotic association. Planta.

[70] Johansson C, Bergman B. Reconstitution of the symbiosis of *Gunnera manicata* Linden: cyanobacterial specificity. New Phytologist. 1994;:643–52.

[71] Cohen M, Meeks J (1997). A hormogonium regulating locus, *hrmUA*, of the cyanobacterium *Nostoc punctiforme* strain ATCC 29133 and its response to an extract of a symbiotic plant partner *Anthoceros punctatus*. Molecular Plant-Microbe Interactions.

[72] Campbell E, Wong F, Meeks J (2003). DNA binding properties of the HrmR protein of *Nostoc punctiforme* responsible for transcriptional regulation of genes involved in the differentiation of hormogonia. Mole Microbiol.

[73] Ekman M, Picossi S, Campbell E. L, Meeks J. C, Flores E (2013). A *Nostoc punctiforme* sugar transporter necessary to establish a cyanobacterium-plant symbiosis. Plant Physiology.

[74] Ow SY, Noirel J, Cardona T, Taton A, Lindblad P, Stensjö K, Wright PC (2008). Quantitative overview of N _2_ fixation in *Nostoc punctiforme* ATCC 29133 through cellular enrichments and iTRAQ shotgun proteomics. J Proteome Res.

[75] Yu X, Doroghazi JR, Janga SC, Zhang JK, Circello B, Griffin BM, Labeda DP, Metcalf WW (2013). Diversity and abundance of phosphonate biosynthetic genes in nature. Proc Natl Acad Sci USA.

[76] Vining L, Stuttard C (1995). Chloramphenicol. Biotechnology.

[77] Vining L, Westlake D, Vandamme E (1984). Chloramphenicol: properties, biosynthesis, and fermentation. Biotechnology of Industrial Antibiotics.

[78] Mosher RH, Camp DJ, Yang K, Brown MP, Shaw WV, Vining LC (1995). Inactivation of chloramphenicol by *O*-phosphorylation a novel resistance mechanism in *Streptomyces venezuelae* ISP5230, a chloramphenicol producer. J Biol Chem.

[79] Campbell EL, Christman H, Meeks JC (2008). Dna microarray comparisons of plant factor-and nitrogen deprivation-induced hormogonia reveal decision-making transcriptional regulation patterns in *Nostoc punctiforme*. J Bacteriol.

[80] Risser DD, Chew WG, Meeks JC (2014). Genetic characterization of the hmp locus, a chemotaxis-like gene cluster that regulates hormogonium development and motility in *Nostoc punctiforme*. Mole Microbiol.

[81] Warshan D, Espinoza JL, Stuart RK, Richter RA, Kim S-Y, Shapiro N, Woyke T, Kyrpides NC, Barry K, Singan V (2017). Feathermoss and epiphytic *Nostoc* cooperate differently: expanding the spectrum of plant–cyanobacteria symbiosis. ISME J.

[82] Warshan D, Liaimer A, Pederson E, Kim S-Y, Shapiro N, Woyke T, Altermark B, Pawlowski K, Weyman PD, Dupont CL, Rasmussen U. Genomic changes associated with the evolutionary transitions of *Nostoc* to a plant symbiont. Mole Biol Evol. 2018; 029. 10.1093/molbev/msy029. 10.1093/molbev/msy029 PMC5913679 29554291

[83] Schuergers N, Mullineaux CW, Wilde A (2017). Cyanobacteria in motion. Current Opinion Plant Biol.

[84] Joneson S, O’Brien H (2017). A molecular investigation of free-living and lichenized *Nostoc* sp. and symbiotic lifestyle determination. Bryologist.

[85] Dittmann E, Gugger M, Sivonen K, Fewer D. P (2015). Natural product biosynthetic diversity and comparative genomics of the cyanobacteria. Trends Microbiol.

[86] Pearson LA, Dittmann E, Mazmouz R, Ongley SE, D’Agostino PM, Neilan BA (2016). The genetics, biosynthesis and regulation of toxic specialized metabolites of cyanobacteria. Harmful Algae.

[87] Kaasalainen U, Jokela J, Fewer DP, Sivonen K, Rikkinen J (2009). Microcystin production in the tripartite cyanolichen *Peltigera leucophlebia*. Molecular Plant-Microbe Interactions.

[88] Kaasalainen U, Fewer DP, Jokela J, Wahlsten M, Sivonen K, Rikkinen J (2012). Cyanobacteria produce a high variety of hepatotoxic peptides in lichen symbiosis. Proc Natl Acad Sci USA.

[89] Hoffmann D, Hevel JM, Moore RE, Moore BS (2003). Sequence analysis and biochemical characterization of the nostopeptolide A biosynthetic gene cluster from *Nostoc* sp. GSV224. Gene.

[90] Becker JE, Moore RE, Moore BS (2004). Cloning, sequencing, and biochemical characterization of the nostocyclopeptide biosynthetic gene cluster: molecular basis for imine macrocyclization. Gene.

[91] Liaimer A, Helfrich EJ, Hinrichs K, Guljamow A, Ishida K, Hertweck C, Dittmann E (2015). Nostopeptolide plays a governing role during cellular differentiation of the symbiotic cyanobacterium *Nostoc punctiforme*. Proc Natl Acad Sci USA.

[92] Golakoti T, Yoshida WY, Chaganty S, Moore RE (2001). Isolation and structure determination of nostocyclopeptides A1 and A2 from the terrestrial cyanobacterium *Nostoc* sp. ATCC53789. J Nat Prod.

[93] Mason C, Edwards K, Carlson R, Pignatello J, Gleason F, Wood J (1982). Isolation of chlorine-containing antibiotic from the freshwater cyanobacterium *Scytonema hofmanni*. Science.

[94] Pignatello JJ, Porwoll J, Carlson RE, Xavier A, Gleason FK, Wood JM (1983). Structure of the antibiotic cyanobacterin, a chlorine-containing *γ*-lactone from the freshwater cyanobacterium *Scytonema hofmanni*. J Org Chem.

[95] Gleason FK, Baxa CA (1986). Activity of the natural algicide, cyanobacterin, on eukaryotic microorganisms. FEMS Microbiol Letters.

[96] Gleason FK, Case DE (1986). Activity of the natural algicide, cyanobacterin, on angiosperms. Plant Physiology.

[97] Chain P, Grafham D, Fulton R, Fitzgerald M, Hostetler J, Muzny D, Ali J, Birren B, Bruce D, Buhay C (2009). Genome project standards in a new era of sequencing. Science.

[98] Ahmadjian V (1989). Studies on the isolation and synthesis of bionts of the cyanolichen *Peltigera canina* (*Peltigeraceae*). Plant Systematics and Evolution.

[99] Stocker-Wörgötter E, Türk R (1991). Artificial resynthesis of thalli of the cyanobacterial lichen *Peltigera praetextata* under laboratory conditions. Lichenologist.

[100] Yoshimura I, Kurokawa T, Yamamoto Y, Kinoshita Y (1993). Development of lichen thalli in vitro. Bryologist.

[101] Stocker-Worgötter E, Türk R (1994). Artificial resynthesis of the photosymbiodeme *Peltigera leucophlebia* under laboratory conditions. Cryptogamic Botany.

[102] Yoshimura I, Kurokawa T, Yamamoto Y, Kinoshita Y (1994). In vitro development of the lichen thallus of some species of *Peltigera*. Cryptogamic Botany.

[103] Stocker-Wörgötter E (1995). Experimental cultivation of lichens and lichen symbionts. Can J Botany.

[104] Kluge M, Mollenhauer D, Mollenhauer R. *Geosiphon pyriforme* (kützing) von wettstein, a promising system for studying endocyanoses. In: Progress in Botany. Springer: 1994. p. 130–41.

[105] Rai AN (1990). Handbook of Symbiotic Cyanobacteria.

[106] Friedl T, Büdel B, Nash TH (2008). Photobionts. Lichen Biology.

[107] Yoshimura I, Yamamoto Y, Nakano T, Finnie J, Kranner I, Beckett R (2002). Isolation and culture of lichen photobionts and mycobionts. Protocols in Lichenology—Culturing, Biochemistry, Physiology and Use in Biomonitoring.

[108] Nilsson M, Rasmussen U, Bergman B (2006). Cyanobacterial chemotaxis to extracts of host and nonhost plants. FEMS Microbiol Ecol.

[109] Xavier BB, Miao VPW, Jonsson ZO, Andresson OS (2012). Mitochondrial genomes from the lichenized fungi *Peltigera membranacea* and *Peltigera malacea*: Features and phylogeny. Fungal Biol.

[110] Bonfield JK, Whitwham A (2010). Gap5 – editing the billion fragment sequence assembly. Bioinformatics.

[111] Bankevich A, Nurk S, Antipov D, Gurevich AA, Dvorkin M, Kulikov AS, Lesin VM, Nikolenko SI, Pham S, Prjibelski AD (2012). Spades: a new genome assembly algorithm and its applications to single-cell sequencing. J Comput Biol.

[112] Bolger AM, Lohse M, Usadel B (2014). Trimmomatic: a flexible trimmer for illumina sequence data. Bioinformatics.

[113] Wu Y-W, Tang Y-H, Tringe SG, Simmons BA, Singer SW (2014). Maxbin: an automated binning method to recover individual genomes from metagenomes using an expectation-maximization algorithm. Microbiome.

[114] Sahlin K, Vezzi F, Nystedt B, Lundeberg J, Arvestad L (2014). Besst – efficient scaffolding of large fragmented assemblies. BMC Bioinformatics.

[115] Sahlin K, Chikhi R, Arvestad L (2016). Assembly scaffolding with pe-contaminated mate-pair libraries. Bioinformatics.

[116] Walker BJ, Abeel T, Shea T, Priest M, Abouelliel A, Sakthikumar S, Cuomo CA, Zeng Q, Wortman J, Young SK (2014). Pilon: an integrated tool for comprehensive microbial variant detection and genome assembly improvement. PloS ONE.

[117] Menzel P, Ng K. L, Krogh A. Fast and sensitive taxonomic classification for metagenomics with kaiju. Nature Communications. 2016;7. 10.1038/ncomms11257 PMC4833860 27071849

[118] Patil K. R, Roune L, McHardy A. C (2012). The phylopythias web server for taxonomic assignment of metagenome sequences. PloS ONE.

[119] Altschul S, Madden T, Schaffer A, Zhang J, Zhang Z, Miller W, Lipman D (1997). Gapped BLAST and PSI-BLAST: a new generation of protein database search programs. Nucleic Acids Res.

[120] Parks DH, Imelfort M, Skennerton CT, Hugenholtz P, Tyson GW (2015). Checkm: assessing the quality of microbial genomes recovered from isolates, single cells, and metagenomes. Genome Res.

[121] Tatusova T, DiCuccio M, Badretdin A, Chetvernin V, Nawrocki EP, Zaslavsky L, Lomsadze A, Pruitt KD, Borodovsky M, Ostell J (2016). Ncbi prokaryotic genome annotation pipeline. Nucleic Acids Res.

[122] Hyatt D, Chen G-L, LoCascio PF, Land ML, Larimer FW, Hauser LJ. Prodigal: prokaryotic gene recognition and translation initiation site identification. BMC Bioinformatics. 2010; 11. 10.1186/1471-2105-11-11. 10.1186/1471-2105-11-119 PMC2848648 20211023

[123] Carver T, Harris SR, Berriman M, Parkhill J, McQuillan JA (2012). Artemis: an integrated platform for visualization and analysis of high-throughput sequence-based experimental data. Bioinformatics.

[124] Pati A, Ivanova NN, Mikhailova N, Ovchinnikova G, Hooper SD, Lykidis A, Kyrpides NC (2010). GenePRIMP: a gene prediction improvement pipeline for prokaryotic genomes. Nature Methods.

[125] Jones P, Binns D, Chang H-Y, Fraser M, Li W, McAnulla C, McWilliam H, Maslen J, Mitchell A, Nuka G, Pesseat S, Quinn AF, Sangrador-Vegas A, Scheremetjew M, Yong S-Y, Lopez R, Hunter S (2014). InterProScan 5: genome-scale protein function classification. Bioinformatics.

[126] Marchler-Bauer A, Zheng C, Chitsaz F, Derbyshire MK, Geer LY, Geer RC, Gonzales NR, Gwadz M, Hurwitz DI, Lanczycki CJ, Lu F, Lu S, Marchler GH, Song JS, Thanki N, Yamashita RA, Zhang D, Bryant SH (2013). CDD: conserved domains and protein three-dimensional structure. Nucleic Acids Res.

[127] Lowe T, Eddy S (1997). tRNAscan-SE: A program for improved detection of transfer RNA genes in genomic sequence. Nucleic Acids Res.

[128] Lagesen K, Hallin P, Rodland EA, Staerfeldt H-H, Rognes T, Ussery DW (2007). RNAmmer: consistent and rapid annotation of ribosomal RNA genes. Nucleic Acids Res.

[129] Nawrocki EP, Eddy SR (2013). Infernal 1.1: 100-fold faster RNA homology searches. Bioinformatics.

[130] Grissa I, Vergnaud G, Pourcel C (2007). CRISPRFinder: a web tool to identify clustered regularly interspaced short palindromic repeats. Nucleic Acids Research.

[131] Edgar RC. PILER-CR: Fast and accurate identification of CRISPR repeats. BMC Bioinformatics. 2007; 8. 10.1186/1471-2105-8-1. 10.1186/1471-2105-8-18 PMC1790904 17239253

[132] Langmead B, Salzberg SL (2012). Fast gapped-read alignment with Bowtie 2. Nature Methods.

[133] Schaper S, Messer W (1995). interaction of the initiator protein DnaA of *Escherichia coli* with its DNA target. J Biol Chem.

[134] Siguier P, Perochon J, Lestrade L, Mahillon J, Chandler M (2006). ISfinder: the reference centre for bacterial insertion sequences. Nucleic Acids Res.

[135] Wu M, Scott AJ (2012). Phylogenomic analysis of bacterial and archaeal sequences with amphora2. Bioinformatics.

[136] Edgar RC (2004). Muscle: multiple sequence alignment with high accuracy and high throughput. Nucleic Acids Res.

[137] Wu M, Chatterji S, Eisen JA (2012). Accounting for alignment uncertainty in phylogenomics. PloS ONE.

[138] Stamatakis A (2014). Raxml version 8: a tool for phylogenetic analysis and post-analysis of large phylogenies. Bioinformatics.

[139] Anisimova M, Gascuel O (2006). Approximate likelihood-ratio test for branches: A fast, accurate, and powerful alternative. Systematic Biol.

[140] Kurtz S, Phillippy A, Delcher A, Smoot M, Shumway M, Antonescu C, Salzberg S. Versatile and open software for comparing large genomes. Genome Biology. 2004; 5(2). 10.1186/gb-2004-5-2-r1. 10.1186/gb-2004-5-2-r12 PMC395750 14759262

[141] Darling A, Mau B, Blattner F, Perna N (2004). Mauve: Multiple alignment of conserved genomic sequence with rearrangements. Genome Research.

[142] Li L, Stoeckert CJ, Roos DS (2003). Orthomcl: identification of ortholog groups for eukaryotic genomes. Genome Res.

[143] Fischer S, Brunk BP, Chen F, Gao X, Harb OS, Iodice JB, Shanmugam D, Roos DS, Stoeckert CJ. Using orthomcl to assign proteins to orthomcl-db groups or to cluster proteomes into new ortholog groups. Current Protocols Bioinforma. 2011;CHAPTER:Unit–6.1219. 10.1002/0471250953.bi0612s35. 10.1002/0471250953.bi0612s35 PMC3196566 21901743

[144] Tatusov R, Koonin E, Lipman D (1997). A genomic perspective on protein families. Science.

[145] Galperin MY, Makarova KS, Wolf YI, Koonin EV (2015). Expanded microbial genome coverage and improved protein family annotation in the COG database. Nucleic Acids Res.

