# Supplementary material for: Distinctive characters of Nostoc genomes in cyanolichens
Source: BMC Genomics. 2018 Jun 5;19:434. doi: 10.1186/s12864-018-4743-5 (PMC5987646; doi:10.1186/s12864-018-4743-5)
Supplement: Supplementary file 2 — Supporting Information (SI) Appendix. (PDF 8156 kb) [file 12864_2018_4743_MOESM2_ESM.pdf]

Distinctive characters of *Nostoc* genomes in cyanolichens

Andrey N. Gagunashvili[1] & Ólafur S. Andrésson[1]

Supporting Information (SI) Appendix

[1] *Faculty of Life and Environmental Sciences, University of Iceland, Sturlugata 7, 101 Reykjavik, Iceland*

Table of Contents

**Supplementary text S1**	Genome comparison	4
**Supplementary text S2**	Comparison to minimal bacterial and cyanobacterial gene sets	6
**Supplementary text S3**	Identification of genes specific to symbiotic *Nostoc* strains	9
**Supplementary text S4**	Secondary metabolites	14
**Supplementary text S5**	Summary discussion	16
**Figure S1**	Nucleotide sequence alignment of the ends of the linear replicons from *Nostoc* sp. N6	19
**Figure S2**	Codon usage of genes encoded on the *Nostoc* sp. N6 chromosome and linear replicon pNPM8	20
**Figure S3**	Origins of replication of the *Nostoc* sp. N6 and *Nostoc* sp. '*Lobaria pulmonaria* cyanobiont' chromosomes	21
**Figure S4**	Dot plot comparisons of lichen-associated *Nostoc* strains and *N. punctiforme*	22
**Figure S5**	Genome sequence alignment of lichen-associated *Nostoc* strains and *N. punctiforme*	23
**Figure S6**	Multiple alignment of $tRNA^{fMet}$ genes from symbiotic *Nostoc* strains	24
**Figure S7**	Reactions catalyzed by ThyA and ThyX thymidylate synthases	25
**Figure S8**	Simplified scheme of pyrimidine metabolism (KEGG pathway 00240)	26
**Figure S9**	Arrangement of *nif* clusters in the *Nostoc* sp. N6 and *Nostoc* sp. '*Lobaria pulmonaria* cyanobiont' genomes	27
**Figure S10**	Arrangement of *vnf* clusters in *Nostoc* genomes	28
**Figure S11**	Multiple nucleotide sequence alignment of introns intervening the ribonucleoside-diphosphate reductase *nrdJ* genes in selected *Nostoc* and *Anabaena* strains	29
**Figure S12**	Multiple protein sequence alignment of NrdJ ribonucleoside-diphosphate reductases from selected *Nostoc* and *Anabaena* strains with *in silico* excised intervening sequences	30
**Figure S13**	Simplified scheme of murein biosynthesis	32
**Figure S14**	Neighbor-joining phylogenetic tree based on the protein sequences of selected D-Ala-D-Ala ATP grasp ligases	33
**Figure S15**	D-Ala-D-Ala ligase 3 gene clusters of lichen-associated *Nostoc* strains	34
**Figure S16**	Phosphorylation of chloramphenicol by chloramphenicol phosphotransferase (CPT)	35
**Figure S17**	Protein sequence alignment of chloramphenicol phosphotransferases	35
**Figure S18**	Gene clusters encoding glycolipid synthases, polyketide synthases and non-ribosomal peptide synthases in the *Nostoc* sp. N6 and *Nostoc* sp. '*Lobaria pulmonaria* cyanobiont' genome	36
**Figure S19**	Nosperin gene cluster in *Nostoc* sp. N6 and its remnants in the *L. pulmonaria* cyanobiont	49
**Figure S20**	Structures and putative biosynthetic gene clusters of cyanobacterin and nostoclides	50
**Figure S21**	Workflow for extracting orthologous groups specific to different clades	51
**Table S1**	Assemblies of *Nostoc* genomes	52
**Table S2**	Completeness and contamination of *Nostoc* genome assemblies assessed with CheckM	53

Table S3 COG category assignment of the proteins encoded in the genome of *Nostoc* sp. N6 . . . 54
Table S4 COG category assignment of the proteins encoded in the genome of *Nostoc* sp. '*Lobaria pulmonaria* cyanobiont' . . . 55
Table S5 COG category assignment of the proteins encoded in the genomes of *Nostoc* and *Anabaena* strains . . . 56
Table S6 Scytonemin biosynthesis genes of *Nostoc punctiforme* and their presence in lichen-associated *Nostoc* strains . . . 57
Table S7 Number of transposase genes in the *Nostoc* sp. N6 and *Nostoc* sp. '*Lobaria pulmonaria* cyanobiont' genomes . . . 58
Table S8 IS family composition of the *Nostoc* sp. N6 and *Nostoc* sp. '*Lobaria pulmonaria* cyanobiont' genomes . . . 59
Table S9 Intein-containing proteins encoded in the genome of *Nostoc* sp. N6 . . . 60
Table S10 Distribution of thymidilate synthase genes among Cyanobacteria . . . 61
Table S11 Intervening sequences in ribonucleoside-diphosphate reductase *nrdJ* genes from selected *Nostoc* and *Anabaena* strains . . . 62
Table S12 Expression of phospholipases C and D by the *Peltigera membranacea* mycobiont . . . 63
Table S13 Gene clusters encoding glycolipid synthases, polyketide synthases and non-ribosomal peptide synthases in the *Nostoc* sp. N6 and *Nostoc* sp. '*Lobaria pulmonaria* cyanobiont' genomes . . . 64
Table S14 Analysis of adenylation domains present in the non-ribosomal peptide synthases encoded in the the *Nostoc* sp. N6 and *Nostoc* sp. '*Lobaria pulmonaria* cyanobiont' genomes . . . 65
References . . . 67

Supplementary Text S1.

Genome comparison

Transposons and inteins. Large scale genome comparisons of lichen-associated strains with *N. punctiforme* PCC 73102 reveal a low level of synteny between them (Figure S4) indicating high genome plasticity and genome shuffling in these strains (Figure S5). This may be largely due to the activity of mobile elements. The number of genes belonging to the mobilome category (X; transposase and phage genes) is nearly 2.5 fold higher in *Nostoc* sp. N6 than in the cyanobiont of *L. pulmonaria* and one of the highest among *Nostoc* and *Anabaena* strains (Tables S3-S5), suggesting that *Nostoc* sp. N6 has a higher degree of genome plasticity. Genome plasticity has been suggested as an adaptive strategy allowing prokaryotes to diversify in a way similar to sexual reproduction in eukaryotes (Filee et al., 2007; Lin et al., 2011). It has been pointed out that a high frequency of mobile elements located in genomes would facilitate this adaptive strategy (Frangeul et al., 2008). Careful manual annotation of the *Nostoc* sp. N6 genome taking into account translation frameshifting, common in open reading frames (ORFs) encoding transposases (Chandler and Fayet, 1993), allowed identification of 97 intact full-length transposase genes and 90 transposase pseudogenes. In the genome of the *L. pulmonaria* cyanobiont most transposase genes have undergone pseudogenezation with only 36 intact genes versus 117 pseudogenes (Table S7). It should be noted that some transposase genes in other Nostocales genomes were found to be incorrectly annotated, e.g. as two adjacent ORFs rather than a single ORF with translation frameshifting (Chandler and Fayet, 1993), resulting in an overestimate for the number of transposase genes.

Insertion sequence (IS) elements, often in association with transposase genes, are particularly abundant in the genomes of mutualistic symbionts and some pathogens that have recently transitioned or are transitioning to an obligate, host-associated lifestyle (Moran and Plague, 2004; Newton and Bordenstein, 2011; Schmitz-Esser et al., 2011; Kleiner et al., 2013). In contrast, they are absent in the reduced genomes of most obligate, host-restricted, clonally propagating bacteria that have been associated with their hosts over long evolutionary time periods. For example, ISs are absent in the majority of the genomes of symbionts classified as obligately intracellular, including chloroplasts (Newton and Bordenstein, 2011; Bordenstein and Reznikoff, 2005).

The IS family composition of the two genomes differed markedly (Table S8). For example, the *ISNCY* family, only found in cyanobacteria (Lin et al., 2011), is represented by 4 copies in the genome of the *L. pulmonaria* cyanobiont and none in the *Nostoc* sp. N6 genome. IS numbers therefore do not reflect the close phylogenetic relationship between these two strains nor their mode of propagation.

Nostoc sp. N6 has a high number of transposable elements and inteins (intervening protein sequences that are excised in the process of protein splicing (Gogarten et al., 2002) (Tables S3 and S5). The best studied case of inteins in Cyanobacteria is in DnaE (the α subunit of DNA polymerase III) encoded by two different ORFs and assembled by trans-splicing (Wu et al., 1998). The genome of *Nostoc* sp. N6 encodes 5 proteins with inteins (locus tags NPM_0997, NPM_1656, NPM_2566, NPM_2586 and NPM_5024; Table S9) whereas the genome of the *L. pulmonaria* cyanobiont contains none. While some of the intein-containing proteins have functional protein homologs encoded in the genome of *Nostoc* sp. N6 there is little sequence identity between the homologs, except for DnaB helicase (Table S9). All the intein-containing proteins, except for the ribonucleoside-triphosphate reductase (NPM_2566) and superfamily II DNA/RNA helicase (NPM_5024) have homologs with the same proposed function without inteins in the *Nostoc* sp. N6 genome (Table S9).

An intron in the tRNA^{fMet} gene. In addition to the group I intron of the tRNA^{Leu}(UAA) gene conserved throughout Cyanobacteria (including plastids), another group I self-splicing intron was found in the anticodon loop of the initiator methionine tRNA (tRNA^{fMet}) gene in *Nostoc* sp. N6. The absence of this intron in the tRNA^{fMet} gene of the *L. pulmonaria* cyanobiont is consistent with its sporadic distribution in Cyanobacteria (Paquin et al., 1997). An identical tRNA^{fMet} intron was found in Nostoc sp. 213 and the metagenome of *P. membranacea*, but not in any other lichen-associated *Nostoc* strains sequenced in this study nor in *N. punctiforme* (Figure S6).

Supplementary Text S2.

Comparison to minimal bacterial and cyanobacterial gene sets

Pyrimidine metabolism. The most prominent differences were observed for the proteins involved in pyrimidine metabolism (Kyoto Encyclopedia of Genes and Genomes (KEGG) pathway 00240). For example, *Nostoc* sp. N6 and *Nostoc* sp. '*L. pulmonaria* cyanobiont' differ in the nature of their thymidylate synthases. All cellular organisms need thymidine as thymidine triphosphate (dTTP) is essential for DNA replication. Cells can produce thymidylate either by phosphorylation of thymidine using thymidine kinases (EC:2.7.1.21) or via thymidylate synthase from deoxyuridine monophosphate (dUMP) by methylation of the pyrimidine at position 5. There are two distinct thymidylate synthase enzymes: ThyA (EC:2.1.1.45) and ThyX (EC:2.1.1.148) (Myllykallio et al., 2002; Leduc et al., 2004), both using 5,10- methylene tetrahydrofolate as a methyl donor. There is no sequence homology between the two enzymes, and their mechanisms of action differ in that ThyX has tightly associated FAD as a cofactor mediating electron transfer from NADPH (Leduc et al., 2004) (Figure S7). *Nostoc* sp. N6 was found to encode ThyX, while the genome of *Nostoc* sp. '*L. pulmonaria* cyanobiont' encodes ThyA. ThyX is more common than ThyA in Cyanobacteria (Table S10) and *thyX* genes can be found in many photosynthetic, anaerobic or microaerophilic bacteria, suggesting that the ThyX pathway is preferentially used in species where the NADP/NADPH pool is kept relatively reduced (Leduc et al., 2004). In agreement with this proposal, the *thyX* gene from microaerophilic *Campylobacter jejuni* has been found to complement an *Escherichia coli thyA* mutant only under oxygen-limiting conditions (Giladi et al., 2002).

It is not clear whether the occurence of *thyA* and *thyX* genes in lichen-associated *Nostoc* strains reflects the differences of thallus morphology of bipartite and tripartite lichens. Since the bulk of the lichen thallus consists of the heterotrophic mycobiont, the thallus interior is microaerobic (Millbank, 1977). In contrast, cyanobacteria in cephalodia of tripartite lichens, such as *L. pulmonaria*, are exposed to a more aerobic environment (Millbank, 1977). It has also been suggested that ThyA proteins are preferred for the replication of large bacterial genomes since they are catalytically more efficient (Escartin et al., 2008). However, genomes of most *Nostoc* strains, despite being relatively large, encode ThyX. In culture the cyanobiont of *L. pulmonaria* exhibited much slower growth than *Nostoc* sp. N6, despite encoding the ThyA thymidylate synthase. It is possible that each thymidylate synthase is advantageous under certain conditions. Occurrence of both thymidilate synthase genes in *Nostoc* sp. 210A, with the *thyX* being a pseudogene (Table S10), is consistent with this idea.

The homolog of dCTP deaminase (Dcd; EC:3.5.4.13) was initially not found in the *Nostoc* sp. '*L. pulmonaria*' genome.This enzyme hydrolyzes deoxycytidine triphosphate (dCTP) yielding deoxyuridine triphosphate (dUTP). dUTP is a natural precursor of dTTP and can be incorporated directly into DNA to create A:U base pairs, as the replicative polymerase does not discriminate between dUTP and dTTP (Figure S8). Independent of dUTP production, dTTP can be synthesized through deoxycytidylate (dCMP) deaminase (Dctd; EC:3.5.4.12). dCMP deaminase is an enzyme that participates in dTTP biosynthesis throughout the cell cycle via conversion of dCMP to dUMP, with capacity to produce adequate quantities of dTTP to sustain DNA replication (McIntosh and Haynes, 1984). dCMP deaminase has been shown to be expressed at a constant level during the cell cycle in the budding yeast *Saccharomyces cerevisiae*, enabling the enzyme to expand the dUMP pool both during and between successive S phases (McIntosh and Haynes, 1984; McIntosh et al., 1986). Although the gene encoding dCMP deaminase was found in the genome of *Nostoc* sp. '*L. pulmonaria* cyanobiont', it does not carry any genes for dCTP diphosphatase (EC:3.6.12) or deoxycytidine kinase

(EC:2.7.1.14) to produce dCMP. A more thorough bioinformatic search uncovered a protein candidate with low homology to conserved dCTP deaminase domains in the *Nostoc* sp. '*L. pulmonaria*' genome (locus tag NLP_1863; TIGR02274, E-value 1.97e-03; COG0717, E-value 0.73). Interestingly, no dCTP deaminase gene was found in *Nostoc* sp. 210A.

Split ribonucleotide reductase enzymes. Apart from the route involving dCTP deaminase, dUTP can be synthesized directly from uridine diphosphate (UDP) by sequential action of ribonucleoside reductase (RNR; EC: 1.17.4.1) and nucleoside diphosphate kinase (Ndk; EC:2.7.4.6) (Figure S8). RNRs are universally essential, as they provide dNTPs needed for DNA replication and repair (Torrents, 2014). RNRs listed in the minimal bacterial set – NrdE and NrdF – belong to the class Ib aerobic RNRs, comprised of two major (α) subunits and two minor (β) subunits. Genomes of many cyanobacteria encode the B12-dependent, oxygen-indifferent (class II) ribonucleotide reductase (NrdJ) as well as the anaerobic (class III) ribonucleotide reductase (NrdD) together with an activating protein (NrdG). Typically NrdJ is encoded by a single *nrdJ* gene (Torrents, 2014). An exceptional NrdJ has been identified in *Pseudomonas aeruginosa*, and this enzyme differs from all so far known class II RNRs, as it is split and encoded by two adjacent ORFs, namely *nrdJa* and *nrdJb*, separated by 16 bp (Crona et al., 2015). The *Nostoc punctiforme nrdJ* gene has also been found to be split, namely by insertion of a 383 bp group I intron (Meng et al., 2007). Bioinformatic analysis of *nrdJ* coding regions in several *Nostoc* and *Anabaena* strains indicates that almost all of them are interrupted by different intervening sequences, with some genes incorrectly annotated as pseudo (Table S11). The majority of the analyzed *nrdJ* genes encode one or two inteins. The *nrdJ* gene in *Anabaena* sp. 90 is interrupted by both an intein sequence and a group II intron (based on the presence of the conserved sequences at the 5′ and 3′ splice sites ...↓GUGYG...AY↓...) with an encoded reverse transcriptase/maturase (ANA_C12699–ANA_C12701). In other strains, including four symbiotic ones, the *nrdJ* gene is interrupted by non-repetitive insertion sequences. For example, the *nrdJ* gene in *Nostoc* sp. '*L. pulmonaria* cyanobiont' was initially annotated as a pseudogene (NLP_3546) due to interruption by an nsertion sequence. The homologous gene in *N. punctiforme* is aslo annotated as a pseudogene (Npun_F2495) with a nearly identical (96% identity within aligned regions) intervening sequence. Conservation of both these sequences and their site of insertion (Figures S11 and S12) is suggestive of introns. The absence of the conserved sequences at the putative splice sites, typical of bacterial group II introns (see above), suggests that these introns belong to group I. Moreover, a guanosine nucleotide (so called ωG), highly conserved for group I introns (Hausner et al., 2014), is located at the 3′ end of all the intron sequences (Figure S11). Splicing of group I introns is processed by two sequential ester-transfer reactions.[3] The exogenous guanosine or guanosine nucleotide (exoG) first docks onto the active G-binding site located in P7, and its 3′-OH is aligned to attack the phosphodiester bond at the 5′ splice site located in P1, resulting in a free 3′-OH group at the upstream exon and the exoG being attached to the 5′ end of the intron. Then the terminal G (omega G) of the intron swaps the exoG and occupies the G-binding site to organize the second ester-transfer reaction: the 3′-OH group of the upstream exon in P1 is aligned to attack the 3′ splice site in P10, leading to the ligation of the adjacent upstream and downstream exons and release of the catalytic intron. Besides genomes of Cyanobacteria, phage genomes are known to contain self-splicing group I introns within genes involved in nucleotide metabolism, particularly RNR genes (Dwivedi et al., 2013). In contrast to introns, inteins are relatively rare in phage RNR genes. The role and origin of intervening sequences in *nrdJ* genes are not clear. The presence of an intron or intein is a rare evolutionary event, particularly in prokaryotes, and is unlikely to be due to chance, especially

when multiple introns and inteins are found within the same gene and at the same sites of insertion (Dwivedi et al., 2013). Strict control of RNR activity is required as dNTP pool imbalances increase mutation rates, replication anomalies, and genome instability (Torrents, 2014). Therefore, intervening sequences may provide a way for effective regulation of RNR expression on both transcriptional (group I and II introns) and/or translational (inteins) levels.

Carbohydrate catabolism. The gene encoding 6-phosphofructokinase (*pfkA*), a key enzyme in the glycolytic pathway, was found to be a pseudogene in *Nostoc* sp. N6 (NPM_0944) suggesting that carbohydrates are catabolized via the oxidative pentose phosphate (OPP) pathway, the primary route of carbon catabolism in cyanobacteria (Pearce and Carr, 1969), or the Entner–Doudoroff (ED) pathway (Chen et al., 2016) rather than the classical Embden-Meyerhof-Parnas pathway for glycolysis. A *pfkA* pseudogene was also found in the metagenome of *P. membranacea* and no *pfkA* was found in the genome of *Nostoc* sp. 232 while *Nostoc* sp. 210A and the cyanobionts of *P. malacea* and *L. pulmonaria* carry intact copies of the *pfkA* gene. It should be noted that the genomes of all symbiotic *Nostoc* strains examined, including that of *N. punctiforme*, encode a phosphoenolpyruvate-protein phosphotransferase (FruB; EC:2.7.1.202), an enzyme that has a scattered distribution in Cyanobacteria and is capable of fructose 6-phosphorylation. The fructose-6-phosphate can enter the pentose phosphate cycle directly through the action of transketolase (Tkt; EC:2.2.1.1) or indirectly via glucose-6-phosphate by the action of phosphoglucose isomerase (Pgi; EC:5.3.1.9), glucose-6-phosphate dehydrogenase (Zwf; EC:1.1.1.49) and 6-phosphogluconate dehydrogenase (Gnd; EC:1.1.1.44).A second phosphorylation step of fructose can be carried out by a 1-phosphofructokinase (FruK; EC:2.7.1.56) yielding β-D-fructose 1,6-bisphosphate, bypassing the PfkA step to enter the glycolytic pathway. However, no *fruK* genes were found in the *Nostoc* genomes. Interestingly, the symbiotic *Nostoc* strains contain a short homolog of 6-gluconophosphate dehydrogenase (Gnd) (295 vs. 476 aa) only sporadically found outside clade II. Similarly, an alternative version of glucose-6-phosphate dehydrogenase (Zwf), is found in many members of the Nostocales.

Potassium transport. In many Cyanobacteria genes for the core Trk potassium transport system are organized in a two-gene operon encoding membrane (TrkG/NtpJ) and NAD-binding (TrkA) components, respectively. A gene encoding TrkG was not found in the *L. pulmonaria* cyanobiont (ort237, Supplementary Excel Table 3). Although the same genome lacks an ortholog of TrkA (ort281, Supplementary Excel Table 3), it encodes another TrkA protein (NLP_5048) albeit with little sequence identity between the two proteins. Typically, Cyanobacteria have several systems for potassium uptake (Hagemann, 2011) and the loss of potassium transporters in the *L. pulmonaria* cyanobiont may have little impact.

Supplementary Text S3.

Identification of genes specific to symbiotic *Nostoc* strains

Hormogonium regulating locus. Hormogonia are relatively short motile filaments, lacking heterocysts, formed by cyanobacteria from the orders Nostocales and Stigonematales. Cyanobacteria differentiate into hormogonia when exposed to environmental stress, or when placed in new media or incubated under red light. Hormogonium differentiation has been found to be crucial for the development of plant-cyanobacteria symbioses (Meeks, 1998). A hormogonium-inducing factor (HIF) secreted by plant hosts induces symbiotic cyanobacteria to differentiate hormogonia and they then dedifferentiate back into nitrogen-fixing filaments after about 48 hours. Cells in the process of hormogonium formation undergo cell division without DNA replication or an increase in mass, leading to cell death if division continues without resumption of vegetative metabolism (Herdman and Rippka, 1988). The capacity of *Nostoc* strains to form hormogonia has been found to be necessary, but not singularly sufficient, for symbiotic competence (Enderlin and Meeks, 1983; Johansson and Bergman, 1994).

A mutant of *N. punctiforme* PCC 73102, characterized by increased hormogonium-dependent infection of the hornwort *Anthoceros punctatus* was recovered in transposon mutagenesis experiments (Cohen and Meeks, 1997). The transposon insertion was next to two ORFs, *hrmU* and *hrmA* (Figure 6), which showed markedly decreased expression. *hrmA* encodes a protein with similarity to a nickel-dependent lactate racemase (COG3875) and a protein domain of unknown function DUF2088 (*pfam09861*), whereas *hrmU* encodes a protein with homology to D-mannonate oxidoreductase. Expression of *hrmUA* is induced by an aqueous extract of *A. punctatus* but not by HIF. The aqueous extract appears to contain a hormogonium repressing factor (HRF) because it suppresses HIF-induced hormogonia formation in the wild type but not in the *hrmUA* mutant. Whereas HIF is released into the immediate vicinity, HRF is probably released into the symbiotic cavity, suppressing further hormogonium formation and permitting heterocyst differentiation. The *hrmUA* genes are preceded by 6 ORFs on the 5′ side – *hrmE*, *unk1*, *unk2*, *hrmK*, *hrmR*, and *hrmI* (Figure 6). HrmE shows similarity to an inositol oxygenase, HrmK to gluconate kinases, HrmR to the LacI/GalR family of transcriptional repressors, and HrmI to a uronate isomerase. The ORFs *unk1* and *unk2* encode proteins of unknown function with SnoaL-like (*pfam12680*) and DUF1815 (*pfam08844*) domains, respectively.

The gluconate kinase homolog encoded by *hrmK* is widespread among bacteria and well conserved among Cyanobacteria where it is thought to feed gluconate into the Entner-Doudoroff (ED) pathway (and possibly the oxidative pentose phosphate pathway, OPP) (Chen et al., 2016). When amino acid sequences of gluconate kinases from eight symbiotic *Nostoc* strains were aligned with sequences from non-symbiotic Nostocaceae, several amino acids appeared conserved in the first group but not in the second group and other bacteria. Interestingly, when fitted onto available structural models (Kraft et al., 2002), most of these amino acid replacements appear in the adaptable lid or outer part of the binding pocket for gluconate, e.g. a tyrosine for phenylalanine substitution between strongly conserved histidine and methionine residues. This may change substrate binding and catalytic properties, which should be amenable to biochemical analyses.

The HrmR protein is a DNA-binding transcriptional regulator that also binds to sugar ligands and represses transcription of its own gene (*hrmR*) and nearby *hrmE* (Campbell et al., 2003). Galacturonate abolishes binding of HrmR to DNA in vitro, suggesting that the in vivo inducer may be a sugar molecule similar to, or containing, galacturonate (Campbell et al., 2003). These observations led to proposal of the following model of HRF-dependent

modulation of HrmR transcriptional regulation (Cohen and Meeks, 1997). HRF enters the *Nostoc* cell and it, or a derivative similar to galacturonate, binds to HrmR, decreasing affinity for the *hrmR* and *hrmE* promoter regions. This derepresses transcription of these genes, leading to inhibition of hormogonia formation (Campbell et al., 2003). The *hrmEKRIUA* genes, as well as the gene encoding a DUF1815 domain, were found in all lichen-associated *Nostoc* strains, as well as *Nostoc* sp. KVJ20 isolated from *Blasia pusilla* (Liaimer et al., 2016) and *Nostoc calcicola* (Zhu et al., 2017), but not in clade I *Nostoc*, implicating this locus in establishment of symbiosis. In the *L. pulmonaria* cyanobiont the *hrmIUA* genes are pseudogenes and therefore are not functional. Moreover the *hrm* cluster is disrupted and found at two separate loci. This suggests that regulation of hormogonium formation is not necessary in the cyanobiont of *L. pulmonaria*, as *Nostoc* is not its primary photobiont and the lichen propagates predominantly clonally (Dal Grande et al., 2012). It should be noted that production of hormogonia by the *L. pulmonaria* cyanobiont was not observed in culture, whereas *Nostoc* strains isolated from *P. membranacea* (N6, 210A, 213 and 232) formed hormogonia and exhibited motility on agar plates.

In *N. punctiforme* PCC 73102 the hormogonium regulating locus is linked to genes involved in sugar transport (Figure 7) (Ekman et al., 2013). The locus contains genes implicated in glucose (*glcP*) and fructose (*frtA1A2BC*) uptake, a carbohydrate-selective porin (*oprB*), and a putative inositol-2-dehydrogenase (*mviM*), probably all transcribed as an operon (Campbell et al., 2003). Interestingly, the *mviM* gene, (pseudogene in *N. punctiforme* PCC 73102 and the *L. pulmonaria* cyanobiont) located between *oprB* and *glcP*, encodes a putative sugar oxidoreductase (COG0673) implying oxidation of an imported sugar (e.g. conversion of fructose to mannitol). The same arrangement of *hrm* genes was found in *Nostoc* sp. 210A and the *P. malacea* cyanobiont while in the genomes of *Nostoc* sp. N6 and sp. 232 the sugar uptake genes, together with *hrmE* and *mviM*, are located in another part of the chromosome than the *hrmKRIUA* genes. GlcP permease in *N. punctiforme* was found to be necessary for establishing the *Nostoc*-*Anthoceros* symbiosis, where the plant provides the cyanobacterium with sugar, supporting its initial growth (Ekman et al., 2013; Picossi et al., 2013). In contrast, in the bipartite cyanolichens the cyanobacterium is the main provider of fixed carbon to the mycobiont, but the fungal spores are thought to mobilize sugars upon germination and resumption of metabolism (Dijksterhuis et al., 2002). It has been hypothesized that the *hrm* locus is involved in HRF-induced synthesis of a metabolite inhibitor of hormogonium differentiation, rather than a carbon catabolic function (Campbell et al., 2003). This metabolite, probably similar to galacturonate (Campbell et al., 2003), binding to the HrmR protein, may act in a positive feedback loop alleviating repression of the *hrm* locus, leading to increased production of the metabolite and at the same time facilitating increased import of sugars such as glucose, fructose and sucrose. Since PfkA (6-phosphofructokinase) appears to be nonessential in symbiotic *Nostoc*, these sugars must be channeled through the OPP pathway or the ED pathway, both producing NADPH reducing equivalents facilitating biosynthesis and decreasing dependence on the non-oxidative pentose phosphate reactions (Calvin cycle). This catabolic shift may simultaneously induce development from hormogonia to vegetative cells and heterocysts. The shift from vegetative cells to heterocysts is accompanied by an increase of the OPP-specific Gnd and an even greater increase in Zwf (Ow et al., 2008), indicating increased carbon flow via the ED pathway. The *hrm* locus appears to be restricted to the *Nostoc* II clade and its sister clade (Figure 4).

D-alanine-D-alanine ligase operon. D-alanine-D-alanine (D-Ala-D-Ala) ligases belong to the group of ATP-grasp enzymes that possess ATP-dependent carboxylate-amine ligase activ-

ity (Iyer et al., 2009). Canonical D-Ala-D-Ala ligases (DdlA and DdlB) participate in peptidoglycan (murein) biosynthesis and catalyze the formation of the D-Ala dipeptide (Figure S13) (Healy et al., 2000). The *N*-terminal region of the D-Ala-D-Ala ligases (*pfam01820*) is thought to be involved in substrate (D-alanine) binding, while the *C*-terminus (*pfam07478*) is thought to be a catalytic domain. Biosynthesis of peptidoglycan requires crosslinking of peptidyl moieties on adjacent glycan strands. The D-Ala-D-Ala transpeptidase, which catalyzes this crosslinking, is the target of β-lactam antibiotics, e.g. penicillin. Glycopeptide antibiotics, such as vancomycin, in contrast, do not inhibit an enzyme, but bind directly to D-alanine-D-alanine moieties and prevent subsequent crosslinking by the transpeptidase. Resistance of certain bacteria to vancomycin has been traced to altered ligases producing D-alanine-D-lactate (VanA and VanB) and D-alanine-D-serine (VanC) instead of D-Ala-D-Ala (Healy et al., 2000).

We identified 3 different D-Ala-D-Ala ligases in the genomes of lichen-associated *Nostoc* strains. One of the enzymes groups with other DdlA proteins on the phylogenetic tree suggesting its involvement in murein D-Ala dipeptide biosynthesis (Figure S14). This is supported by isolation of UDP-*N*-acetylmuramyl-pentapeptide containing D-Ala-D-Ala from *Anabaena cylindrica* (Kodani et al., 1999) and high sensitivity of *A. variabilis* to vancomycin (Matsuhashi et al., 1969).

The other two D-Ala-D-Ala ligases did not have any recognizable *N*-terminal region involved in substrate binding but contain the *C*-terminal domain. One of these enzymes, as deduced by homology analysis and gene neighborhood, is involved in the biosynthesis of a cyanobacterial UV sunscreen, shinorine (Balskus and Walsh, 2010; Gao and Garcia-Pichel, 2011) and ligates serine to the mycosporine-glycine intermediate. The third D-Ala-D-Ala ligase with accompanying enzymes was found only in lichen-associated Nostocs and neither in *N. punctiforme* PCC 73102 nor in free-living strains (Figure S15). Sparse occurrence of homologous proteins in only some genera of Nostocales and Oscillatoriales (Figure S14) suggests its acquisition via horizontal gene transfer. Two lysine modifying enzymes – lysine 2,3-aminomutase (LAM) and lysine methyltransferase (SET) are encoded upstream in the same operon, indicating that lysine may be the substrate for this enzyme. However, as ATP-grasp ligases are involved in a vast range of pathways, it is not clear what molecule lysine is transfered to (Iyer et al., 2009). We suggest that the alternative D-Ala-D-Ala ligase catalyzes transfer of a methylated β-lysine moiety (produced by LAM and SET) onto a yet unknown acceptor substrate. Close proximity of the gene encoding a MATE (NorM) family efflux pump in *Nostoc* sp. N6 and 232 suggests that a synthesized compound may be excreted (Figure S15). Of all β-amino acids, β-lysine seems to be one of the most common in bacteria (Spiteller and von Nussbaum, 2005; von Nussbaum and Spiteller, 2006; Kudo et al., 2014), e.g. as a solubilizing side chain of aminoglycoside antibiotics (streptothricin, albothricin, lysinomycin, myomycin B), or within the periphery of cyclopeptides (capreomycins, tuberactinomycins). Other examples include β-lysine-derived dipeptides, e.g. TAN-1057 A and D, and some other antibiotics, e.g. negamycin, bellenamine, sperabillins A and D. However, the location of the gene encoding a soluble lytic murein transglycosylase (MtlE, COG0741) (Scheurwater et al., 2008) next to the efflux pump gene suggests participation of D-Ala-D-Ala ligase 3 in peptidoglycan biogenesis. Bacterial peptidoglycan biosynthesis begins within the cytoplasm with the synthesis of UDP-MurNAc-L-Ala-D-Glu-X-D-Ala-D-Ala, the so-called Park nucleotide (Figure S13) (Park, 1952).In Gram-negative bacteria, X is usually *meso*-diaminopimelate (*m*-DAP) and in Gram-positive bacteria, X is usually L-lysine (Bugg and Walsh, 1992). The structural appearance of the cyanobacterial cell wall is similar to that of Gram-negative bacteria, but the cyanobacterial peptidoglycan layer is thicker (Golecki, 1988).Furthermore the chemical composition of cyanobacterial peptidoglycan is closer to that of Gram-positive bacteria (Ju-

rgens and Weckesser, 1985). A Park nucleotide containing lysine has been obtained from *A. cylindrica* (Kodani et al., 1999) indicating that Nostocales have a peptidoglycan chemical composition similar to Gram-positive bacteria.This data suggests that D-Ala-D-Ala ligase 3, like the D-Ala-D-Ala ligase 1 (DdlA), may be involved in modification of peptide moieties in peptidoglycans. This gene cluster is characteristic of the lichen associated *Nostoc* strains (Figure 4).

Phosphonate biosynthetic genes. Phosphonates are organophosphorus compounds containing direct carbon-phosphorus bonds and represent a structurally diverse class of natural products, including several bioactive phosphonates, as well as those needed for the synthesis of certain phosphonolipids and phosphonoglycans. Virtually all known phosphonates are derived from phosphoenolpyruvate (PEP) by isomerization to phosphonopyruvate in a thermodynamically unfavorable reaction catalyzed by the enzyme PEP mutase (PepM). This reaction is followed by a thermodynamically favorable decarboxylation step catalyzed by phosphonopyruvate decarboxylase (Ppd). A wide range of C–P biomolecules are then created by diverse biosynthetic steps. The genes encoding these downstream enzymes are usually clustered with the *pepM* and *ppd* genes, a feature that has greatly facilitated genetic and biochemical studies of phosphonate biosynthesis and allowed prediction of the types of C–P molecules produced by the metabolic pathways encoded by these linked genes (Yu et al., 2013). A phylogenetic study of *pepM* from many bacterial genomes with surrounding upstream and downstream genes showed scant correlation between the PEP mutase phylogeny and organismal phylogenies (Yu et al., 2013).

In contrast, a strong correlation between PEP mutase phylogeny and the *pepM* gene neighborhood was observed, represented by 12 distinct groups of nearly identical gene neighborhoods. All of the PEP mutase sequences from each group were found to be monophyletic, suggesting that similarity of PEP mutase is a good predictor of the other genes in the phosphonate biosynthetic pathway (Yu et al., 2013). Strongly conserved phosphonate biosynthetic gene clusters occur in all lichen-associated *Nostoc* strains used in this study (Figure 7a). These clusters are not typical for Cyanobacteria with the sole example being a split pathway found in *Chlorogloeopsis fritschii*, suggesting their acquisition through horizontal transfer. Homologous gene clusters found mostly in *Burkholderia* share almost the same set of genes as well as their order (Figure 7a). These clusters were proposed to be involved in biosynthesis of phosphonolipids with 1-hydroxy-2-aminoethylphosphonate (HAEP) as the head group (Figure 7b) (Yu et al., 2013). PepM, Ppd, AEP transaminase and 2OG-Fe(II) oxygenase are responsible for the formation of HAEP. Phosphocholine cytidyltransferase most likely acts as a CDP-diglyceride synthetase catalyzing the synthesis of CDP-diacylglycerol from CTP and phosphatide. CTP:phospho-ethanolamine cytidylyltransferase uses phospho-ethanolamine and CTP to form the high-energy donor CMP-phosphonate intermediate with release of pyrophosphate. CDP-alcohol phosphatidyltransferase (CDP-APT; *pfam01066*), an essential enzyme in phospholipid biosynthesis, then catalyzes the formation of phosphonolipid with a HAEP head group using CMP-HAEP and a lipid anchor such as diacylglycerol (DAG) or alkyl-acylglycerol (AAG) (Figure 7b).

The bacterial members of the CDP-APT family are known to have different specificities adding different polar head groups on phospholipids. A phylogenomic survey has shown that the different CDP-APTs group in phylogenetic trees according to their predicted substrates (Daiyasu et al., 2005). In addition, the groups of bacterial sequences were in agreement with the main bacterial taxonomic groups. Interestingly, only *Nostoc* sp. 232 has a common cyanobacterial homolog of CDP-APT, while other lichen associated *Nostoc* strains have only

phosphonate-related CDP-APT. HAEP was found to be a head group of sphingophosphonolipids previously characterized in the predatory bacterium *Bacteriovorax stolpii* (Jayasimhulu et al., 2007; Watanabe et al., 2001). Sphingophosphonolipids were not crucial for the viability and predatory behavior of *B. stolpii*, but cell locomotory behavior was altered and fewer and smaller bdelloplasts (interperiplasmic parasitic stage) were produced (Kaneshiro et al., 2008). It is apparent that although not being vital for cell growth, phosphonolipids as constituents of biomembranes may affect cell wall permeability, cell motility and recognition.

Phosphonolipids are known to resist the action of some phospholipases (Metcalf and van der Donk, 2009). In the lichen symbiosis cyanobionts undergo certain structural changes, including less sheath and thinner cell walls (Bergman and Hällbom, 1982; Tschermak-Woess, 1988; Koriem and Ahmadjian, 1986; Boissière, 1987; Bergman et al., 2007), making them potentially more exposed and susceptible in the intrathalline environment. Therefore, phosphonolipids may have a role in protecting lichen cyanobionts from attack by extracellular phospholipases produced and secreted into the intrathalline space by the mycobiont (Ghannoum, 2000). Various intrathalline lichen-associated bacteria have also been shown to have the capacity to produce phosphatases (Sigurbjörnsdóttir et al., 2015) as well as phospholipases (Gagunashvili, personal observations). RNA-Seq mapping revealed expression of genes encoding phospholipases C and D by the *P. membranacea* mycobiont (Table S12). A chemical analysis would be required to verify the presence of phosphonolipids in lichen cyanobionts. A PCR survey with degenerate PCR primers designed from conserved PepM amino acids motifs can also be done to assess the occurrence and distribution of phosphonate gene clusters in cyanolichens. This cluster is characteristic of the *Nostoc* II clade.

Chloramphenicol phosphotransferase. Chloramphenicol is an antibiotic produced by *Streptomyces venezuelae* ATCC 10712 and several other actinomycetes (Vining and Stuttard, 1995). The bacteriostatic activity of chloramphenicol results from its binding to the 50S subunit of the bacterial ribosome blocking peptidyl transferase (Vining and Westlake, 1984). *S. venezuelae* escapes the toxicity of its own lethal secondary metabolite by expressing a chloramphenicol phosphotransferase (CPT) that phosphorylates the primary (C-3) hydroxyl of chloramphenicol (Figure S16) (Mosher et al., 1995). The 3-*O*-phosphoryl-chloramphenicol product is biologically inactive as an antibiotic. Phosphorylation has been observed as a common way to develop resistance to other antibiotics (Cundliffe, 1992). Genes encoding CPT were found in all the lichen-associated *Nostoc* strains (Figure S17), as well as in *Nostoc* sp. KVJ20 and *Nostoc calcicola*. A BLASTP search of the *Nostoc* CPTs against the GenBank database returned only one homolog from other Cyanobacteria, (*Tolypothrix* sp. PCC 7601) followed by hits to proteins from Firmicutes and Proteobacteria, suggesting that CPT genes have very limited distribution in Cyanobacteria and have been acquired via horizontal gene transfer. It is not clear what drives positive selection of CPT genes in lichen-associated *Nostoc* strains. It is possible that they confer resistance to some chloramphenicol-like metabolites produced by the mycobiont or intrathalline bacteria. Many actinomycete strains, including *Streptomyces* spp., have been isolated from lichens and shown to have a broad spectrum of antimicrobial activity (González et al., 2005; da Silva et al., 2011; Saeng-in et al., 2015). It should be noted that the use of vectors conferring resistance to chloramphenicol, e.g. pRL271, commonly used for genetic manipulation of cyanobacteria (Black et al., 1993), may be problematic with lichen-associated *Nostoc* strains due to their innate resistance to chloramphenicol. Genes encoding CPT were almost exclusively found in the *Nostoc* II clade.

Supplementary Text S4.

Secondary metabolites

Cyanobacteria produce a multitude of secondary metabolites, many of them toxic (Dittmann et al., 2015; Pearson et al., 2016). In a recent study, Liaimer et al. (Liaimer et al., 2016) found that *Nostoc* symbionts of the liverwort *Blasia pusilla* more frequently produce nodularin and microcystin type compounds antagonistic to other *Nostoc* strains than free living *Nostoc* from the same locality. Most types of secondary compounds were detected in only 1-4 out of the 20 strains examined. The occurence of the main secondary metabolite pathways in *Nostoc punctiforme*, the *Nostoc* strains from the *Blasia* habitats (Liaimer et al., 2016) and the lichen-derived strains of this study show little overlap.

One of the secondary compounds detected by Liaimer *et al.* (Liaimer et al., 2016) is the polyketide synthase plus non-ribosomal peptide synthase (PKS-NRPS) product nosperin (Kampa et al., 2013). We previously suggested that nosperin might have cytotoxic properties analogous to cyanobiont microcystins (Kaasalainen et al., 2009, 2012) which can serve as protective compounds in cyanolichens, e.g. against grazers. Interestingly, *Nostoc* sp. 232 was found to be devoid of *nsp* genes, but it contains a putative microcystin gene cluster not found in the *nsp* containing *Nostoc* sp. N6 strain. Similarly, the single *Blasia*-habitat *Nostoc* strain showing nosperin does not exhibit any of the other metabolites under study (Liaimer et al., 2016).

Remnants of the *nsp* gene cluster were found on the chromosome of the *L. pulmonaria* cyanobiont (Figure S19), where almost the entire cluster has been deleted, probably due to the absence of selective pressure. This indicates that *nsp* genes are not essential for this lichen symbiosis although nosperin may confer advantage under some conditions, e.g. in terrestrial lichens as opposed to epiphytic ones. Its role may be less crucial for clonally propagating lichens or in tripartite lichens, such as *L. pulmonaria*, where the main provider of photosynthate is a green alga but not the cyanobiont. Nevertheless the *nsp* genes have been found in the tripartite terrestrial *Peltigera leucophlebia* (Gagunashvili and Miao, unpublished).

Whole genome sequencing of the nosperin producer *Nostoc* sp. N6 revealed that the *nsp* gene cluster is located on the chromosome. The abundance of insertion sequences surrounding the cluster and the apparent mixed gene origin suggests that it has been acquired as a genomic island through horizontal transfer and undergone several intragenomic recombination events (Kampa et al., 2013).

The genome of *Nostoc* sp. N6 was also found to encode pathways for the biosythesis of nostopeptolide- (Hoffmann et al., 2003) and banyaside/suomilide-like (Liaimer et al., 2016) compounds as well as nostocyclopeptide (Becker et al., 2004) (Table S13). The nostopeptolide-like cluster seems to be inactive in *Nostoc* sp. N6 due to multiple frameshifts and interruption by ISs, despite being intact in the *P. membranacea* metagenome (GenBank accession GU591312) (Kampa et al., 2013). Nostopeptolide in *Nostoc punctiforme* has been found to be a major hormogonium-repressing factor and therefore considered responsible for cellular differentiation of *Nostoc* (Liaimer et al., 2015). An additional role of nostopeptolide as a chemoattractant of *Nostoc* filaments has also been proposed.

Nostocyclopeptides are cyclic heptapeptides with a unique imino linkage in the macrocyclic ring, isolated from the lichen cyanobiont *Nostoc* sp. ATCC 53789 (Golakoti et al., 2001). The molecular basis for nostocyclopeptide biosynthesis in *Nostoc* sp. ATCC 53789 has been described and involves two NRPS genes (*ncpA* and *ncpB*) as well as genes for biosynthesis of the rare nonproteinogenic amino acid 4-methylproline (4-mPro) (Becker et al., 2004). Two homologous NRPS genes (locus tags NPM_1843 and NPM_1844) were found in the genome

of *Nostoc* sp. N6. Although the 4-mPro cassette was absent in the nostocyclopeptide cluster in N6, there is a set of genes for 4-mPro biosynthesis in a nostopeptolide-like gene cluster (locus tags NPM_0715 and NPM_0717). Interestingly, the order of adenylation domains in NPM_1844, confirmed by mate-pair read remapping and reassembly, is different from NcpB of *Nostoc* sp. ATCC 53789 (mPro-Ser-Ile-Phe vs. Ile-Ser-mPro-Phe), representing a natural example of NRPS domain shuffling (Table S14). The function of nostocyclopeptides in *Nostoc* sp. ATCC 53789 is unknown (Becker et al., 2004).

A *Nostoc* sp. strain isolated from the bipartite lichen *Peltigera canina*, belonging to the same group as *P. membranacea*, was shown to produce the chlorine-containing nostoclides I and II (Figure S20a) (Yang et al., 1993). Nostoclides exhibited moderate cytotoxicity against the mouse neuroblastoma cell lines Neuro-2a CCL and KB CCL 17 (Yang et al., 1993) and their analogs possess phytogrowth- and photosynthesis-inhibiting properties suggesting the potential use of nostoclides as herbicides (Barbosa et al., 2006). Since *P. canina* thalli are usually not substantially contaminated with microorganisms, it has been suggested that nostoclides may be allelopathic agents in the lichen symbiosis. Moreover, a compound with a very similar structure, cyanobacterin, produced by the cyanobacterium *Tolypothrix* sp. PCC 9009 (*Scytonema hofmanni* UTEX 2349) (Mason et al., 1982; Pignatello et al., 1983), was found to inhibit the growth of many cyanobacteria, as well as green algae and angiospems (Gleason and Baxa, 1986; Gleason and Case, 1986) by specifically disrupting the electron transport chain in photosystem II (Gleason and Paulson, 1984; Gleason, 1990). Nothing is known about the molecular basis of nostoclide and cyanobacterin biosynthesis but they are most likely synthesized via the shikimate pathway (Kacan, 2007). Based on the homology with *Tolypothrix* sp. PCC 9009, we identified putative gene clusters for biosynthesis of nostoclide-like compounds in the genomes of *Nostoc* spp. 210A and 232 (Figure S20b).

Supplementary Text S5.

Summary discussion

Draft genome sequences of four more lichen-associated *Nostoc* were included in the analysis to increase the ability to identify possible symbiosis associated genes and gene networks. This set of lichen-associated *Nostoc* genomes was compared to those of symbiotically competent *N. punctiforme* and free-living Nostocs, as well as *Anabaena* strains with complete genomes (Table 1 and Additional file 6). The genome sizes of the symbiotic *Nostoc* strains range from 7.34 Mb to 8.9 Mb and are larger than those of the free-living *Nostoc* and *Anabaena* strains, which range from 5.31 Mb to 7.21 Mb. This size difference may reflect the presence of additional genes necessary for symbiotic competence. The genome of the *L. pulmonaria* cyanobiont exhibits a clear reduction of genome size, also a feature of *Trichormus azollae* ('*Nostoc azollae*' 0708), the cyanobacterial symbiont of the water fern *Azolla filicoides* (Ran et al., 2010). In contrast to *T. azollae*, which is not capable of propagation outside symbiosis (Tang et al., 1990), the cyanobiont of *L. pulmonaria* was able to grow in culture, albeit at a slow rate (this study). Interestingly, *Nostoc* strains isolated from the tripartite lichen *Peltigera aphthosa* also exhibited slow growth in pure culture (Paulsrud et al., 2001). Since slower growth is a common attribute of cyanobionts from tripartite lichens, it is possible this trait confers an advantage for the mycobiont, avoiding overgrowth of cyanobionts since they are "farmed" in cephalodia and their main role is to carry out nitrogen fixation. Nevertheless, it has been shown that the same *Nostoc* strain was present in both bipartite and tripartite lobes of the *P. aphthosa* photosymbiodeme, containing either cyanobacteria or green algae as primary photobionts in different parts of the same thallus (Paulsrud et al., 1998). It is therefore possible that a single *Nostoc* strain forms the physiologically different symbioses that are found in bipartite and tripartite lichens.

Comparative genome analysis of symbiotic and free-living cyanobacteria allowed the identification of several pathways that may contribute to symbiotic competence of *Nostoc* strains. One pathway, encoded by the hormogonium regulating (*hrm*) locus, was previously identified in symbiotically competent *N. punctiforme* and plays a central role in the repression of hormogonia formation. This pathway is similar to pathways of sugar uronate metabolism in heterotrophic non-cyanobacterial prokaryotes (Campbell et al., 2003; Cohen and Meeks, 1997). Although the *hrm* locus has been shown to be important in the *Nostoc*-plant symbiosis, its presence in all of the lichen-associated *Nostoc* strains from this study suggests it is also relevant to establishing *Nostoc*-mycobiont symbioses. Repression of *N. punctiforme* hormogonia is linked to the host release of an unidentified hormogonia-repressing factor and induction of the *hrmA* gene (Meeks, 2003). *hrmA* expression is induced by an aqueous extract of the hornwort *Anthoceros* tissue, leading to the suggestion that a factor in the extract prevents hormogonium formation (Cohen and Meeks, 1997). The flavonoids naringin and, to a lesser degree, neohesperidine and prunine have been found to induce expression of *hrmA* (Cohen and Yamasaki, 2000). *hrmA* expression was also induced by aqueous extracts of *Azolla* spp., and this induction was correlated with the amount of other flavonoids, deoxyanthocyanins (Cohen et al., 2002). In plants, the flavonoid backbone is synthesized by chalcone synthases belonging to type III polyketide synthases (Dao et al., 2011; Seigler, 2012). Chalcone synthase genes have been found in the genomes of ascomycete fungi (Seshime et al., 2005; Hashimoto et al., 2014), including *P. membranacea* and *P. malacea* mycobionts (Gagunashvili, personal observation). Therefore, production of flavonoid compounds with a similar hormogonium repressing activity can be expected in lichen mycobionts.

The *hrm* pathway seems to be inactive in the cyanobiont of *L. pulmonaria* due to pseu-

dogenezation and disruption, and the strain did not show hormogonium production in culture (this study). Therefore, the ability to regulate the formation of hormogonia is likely to be less crucial for *Nostoc* strains from tripartite lichens with cephalodia and/or strains from lichens that reproduce predominantly clonally. It is worth noting that two *Nostoc* isolates from the tripartite *Peltigera aphthosa* did not develop hormogonia under any conditions tested (Paulsrud et al., 2001).

Sugars have been suggested to have a role as chemoattractants for cyanobacteria during during establishment of the plant symbiosis (Rasmussen et al., 1996; Nilsson et al., 2006; Khamar et al., 2010). It has also been suggested that various sugars, including glucose and fructose, repress the formation of hormogonia after the symbiosis has been established (Khamar et al., 2010). Conservation and colocalization of the *hrm* and sugar uptake genes in the *Nostoc* cyanobionts suggests related roles in signaling and regulatory mechanisms important in establishing and maintaining the lichen symbiosis.

Trehalose is a non-reducing disaccharide composed of two α-glucose units. It is a carbohydrate storage compound and an important stress protectant involved in metabolic signaling and regulation of carbohydrate metabolism in fungi (Hounsa et al., 1998; Argüelles, 2000; Dijksterhuis et al., 2002; Doehlemann et al., 2006; Svanström and Melin, 2013). Trehalose often accumulates in resting cells of fungi, e.g. ascospores. The first major step of the transition from dormant ascospores to metabolizing, growing fungal cells is the trehalase-catalyzed breakdown of trehalose to glucose monomers. During early germination in the ascomycete *Talaromyces macrosporus*, the intracellular concentration of glucose peaked briefly and a large amount of glucose (10% of the cell weight or more) was released from the cell into the microenvironment (Dijksterhuis et al., 2002). Similar series of processes may be expected for lichen symbionts. Glucose released upon germination of lichen ascospores can serve as both an attractant and a carbon source for *Nostoc* hormogonia, since hormogonia formation is accompanied by a decrease in photosynthetic CO_2 fixation and a shift to heterotrophy. Consistent with this idea, trehalase genes were found in the genomes of *P. membranacea* and *P. malacea* mycobionts (Gagunashvili, personal observation).

Pathways that may be involved in cell wall biogenesis of lichen cyanobionts were also identified. These pathways may lead to cell wall modifications that are better "tailored" to the intrathalline environment, as well as being recognized as compatible by a mycobiont during establishment of symbiosis. Despite being sheltered by a mycobiont, lichen cyanobionts are subjected to extracellular enzymes and metabolites produced by both the mycobiont and intrathalline bacteria. Therefore, an ability to withstand some unfavorable aspects of this cohabilitation is expected from lichen-associated *Nostoc* strains. Differences in cell wall composition may explain why *N. punctiforme*, originally isolated from a plant symbiosis, as well as the free-living *Nostoc* sp. PCC 7120, were both unable to enter into symbiosis with the *P. aphthosa* mycobiont in thallus incorporation experiments (Paulsrud et al., 2001).

Symbiotically associated *Nostoc* cells show several morphological differences relative to cells in a free-living state (Meeks, 2005). The vegetative cells of symbiotic *Nostoc* spp. are usually larger with a more irregular shape, relatively weak connections between individual cells, and with an aseriate appearance. Moreover, the size difference between vegetative cells and heterocysts is minimal in the symbiotic setting. Physically isolated symbiotic *Nostoc* spp. tend to appear microscopically as unicellular, bicellular or very short filaments. Since the peptidoglycan layer defines the shape of bacterial cells, the peptidoglycan of symbiotic *Nostoc* cells appears to have some structural modifications. Consistent with this idea, we found several genes that are presumably involved in peptidoglycan modification, such as a D-Ala-D-Ala ligase, to be specific for symbiotic *Nostoc* strains.

Lectins produced by mycobionts have long been proposed to be involved in recognition and binding of compatible cyanobacterial and algal partners (Singh and Walia, 2014). Several lectins from a number of both bipartite and tripartite *Peltigera* species have been characterized (Lockhart et al., 1978; Petit, 1982; Petit et al., 1983; Lehr et al., 1995, 2000; Díaz et al., 2009; Feoktistov et al., 2009). These lectins discriminate between preferred and other symbionts. They have been found to bind to the cell wall of lichen-derived, cultured *Nostoc*, as well as to other cultured *Nostoc* strains isolated from different *Peltigera* species, whereas no binding was observed with free-living *Nostoc* cells nor with a *Scytonema* cyanobiont or a green algal phycobiont (Petit, 1982; Petit et al., 1983). These lectins neither bound to the *Nostoc* symbiont of *Anthoceros* nor to a number of free-living cyanobacteria from other genera (Petit, 1982; Petit et al., 1983). Since lectins can be highly specific, it has repeatedly been suggested that they may be key factors in the recognition and selection of compatible symbionts and may induce formation of symbiotic structures by the mycobiont. Selective properties of lectins are associated with reversible binding to carbohydrates exposed on cellular surfaces of photobionts. Some carbohydrate-modifying enzymes in a cyanobiont may therefore be responsible for production of symbiotically specific carbohydrates recognized by a mycobiont. For example, genomes of all symbiotic *Nostoc* strains in this study encode a polysaccharide deacetylase (locus tags NPM_0822 and NLP_5237). The family of polysaccharide deacetylases includes NodB, the nodulation protein B from rhizobia, which acts as a chitooligosaccharide deacetylase and is involved in establishing the *Rhizobium*-legume symbiosis (Freiberg et al., 1997).

```
pNPM8 Left      1 AAATTAACAGACGTAACAGGTTAGATAAGCTTCATGTCTGCAATGAG 47
pNPM8 Right     1 AAATTAACAGACGTAACAGGTTAGATAAGCTTCATGTCTGCAATGAG 47
pNPM10 Left     1 AAATTAACGGACACACCTG------------TTATATTTGCGTTAAC 35
pNPM10 Right    1 AAATTAACGGACACACCTG------------TTATATTTGCGTTAAC 35
pNPM9 Left      1 AAATTAACGGACACACCTA------------TTATGTCTGCGTTAAC 35
pNPM9 Right     1 AAATTAACGGACACACCTG------------TTATGTCTGCATTAAC 35

pNPM8 Left     48 CTTTTAGATAACAGGTTTATGTATGCGTTAACCAGTTAATCAGAACC 94
pNPM8 Right    48 CTTTTAGATAACAGGTTTATGTATGCGTTAACCAGTTAATCAGAACC 94
pNPM10 Left    36 CTGTTAAGCATCTGG-TTATGTCTGCGTTAACTGGTTA--------- 72
pNPM10 Right   36 CTGTTAAGCATCTGG-TTATGTCTGCGTTAACTGGTTA--------- 72
pNPM9 Left     36 CTGTTAAGCATCTGG-TTATGTCTGCGTTAACTGGTTA--------- 72
pNPM9 Right    36 CTGTTAAGCATCTGG-TTATGTCTGCGTTAACTGGTTA--------- 72

pNPM8 Left     95 TGTTCATAGCGTATATATCCACAATCATTGTATAATAAGCGTTTCAG 141
pNPM8 Right    95 TGTTCATAGCGTATATATCCACAATCATTGTATAATAAGCGTTTCAG 141
pNPM10 Left    73 ----------GTATCTCTGCGTTAACAAGTTATC------------- 96
pNPM10 Right   73 ----------GTATCTCTGCGTTAACAAGTTATC------------- 96
pNPM9 Left     73 ----------GCATCTCTGCGTTAACAAGTTATCATGTTTGCAT-AA 108
pNPM9 Right    73 ----------GCATCTCTGCATTAACAAGTTATC---TCTGCATTAA 106

pNPM8 Left    142 CTAATGGTTAGATTTGTAGATGAGTTTATAACAGTATATATGCATTA 188
pNPM8 Right   142 CTAATGGTTAGATTTGTAGATGAGTTTATAACAGTATATATGCATTA 188
pNPM10 Left    97 --------------------------------------CCTGCATTA 105
pNPM10 Right   97 --------------------------------------CCTGCATTA 105
pNPM9 Left    109 CGGGTTAACATCTCTGCATAACTGTT------ATCATGTTTGCA-TA 148
pNPM9 Right   107 CTAGTTAGCATATCTGCATAACTGTT------ATCATGTTTGCA-TA 146

pNPM8 Left    189 TCCTGTTACTACC-TTAGCTAATATGTTATAGATTTA--ATTTTAAA 232
pNPM8 Right   189 TCCTGTTACTACC-TTAGCTAATATGTTATAGATTTA--ATTTTAAA 232
pNPM10 Left   106 ACTGGTTATTGTCGTCATCTAACAGTTTGCCGTCAAAGCATTCTCAA 152
pNPM10 Right  106 ACTGGTTATTGTCGTCATCTAACAGTTTGCCGTCAAAGCATTCTCAA 152
pNPM9 Left    149 ACTGGTTATCATCGTCATCTAACAGTTTGCCGTCAAAGCATTCTCAA 195
pNPM9 Right   147 ACTAGTTAGTATAGTTGCATAACGGGTTATCATGTTTGCA------- 186

pNPM8 Left    233 TATTTAACAGGTT--GTAAACATTGGCTGAGAGATTGTAACA----- 272
pNPM8 Right   233 TATTTAACAGGTT--GTAAACATTGGCTGAGAGATTGTAACA----- 272
pNPM10 Left   153 ATTTTCCCAGGTT-CCCAACCTTTCATAAAATCGTCGTCATC----- 193
pNPM10 Right  153 ATTTTCCCAGGTT-CCCAACCTTTCATAAAATCGTCGTCATC----- 193
pNPM9 Left    196 ATTTTCCCAGGTTCCCCAACCTTTCATAAAATCGTCGTCATC----- 237
pNPM9 Right   187 ----TAACTGGTT-----------------ATCGTCGTCATCTAACA 212

pNPM8 Left    273 -GTTCTATGTAATAACAAGTTATTGATTTTGCCGCGTTCTCGGT--- 315
pNPM8 Right   273 -GTTCTATGTAATAACAAGTTATTGATTTTGCCGCGTTCTCGGT--- 315
pNPM10 Left   194 -TTTTAAGGCGATCGCCG----------TCAAAGCGTTCTCAGACTT 229
pNPM10 Right  194 -TTTTAAGGCGATCGCCG----------TCAAAGCGTTCTCAGACTT 229
pNPM9 Left    238 -TTTAAAGGCGATCGCCGCCAAAGCATCTCAAAGCGTTCTCAA---- 279
pNPM9 Right   213 GTTTGCCAGCGATCGCCC----------TCAAAGCGTTCTCAATCTT 249

pNPM8 Left    316 -----GGTTTTTGA--CTGGCAACTGTGTTGCCGCGTCTCCCACGTC 355
pNPM8 Right   316 -----GGTTTTTGA--CTGGCAACTGTGTTGCCGCGTCTCCCACGTC 355
pNPM10 Left   230 TCCCAAGGTTCCCA---TAGTTA-TGCTGTTTTATTTGCTTCTGGCA 272
pNPM10 Right  230 TCCCAAGGTTCCCA---TAGTTA-TGCTGTTTTATTTGCTTCTGGCA 272
pNPM9 Left    280 -----AGTTCCCCA----GGTTA-TGCCTTTTTATTTTGCCCTGGCA 316
pNPM9 Right   250 TCC--GGTTTCCTAAGTTGGTTA-TGCCTTTTATTTTTTCGCTGGCA 293
```

Figure S1. Nucleotide sequence alignment of the ends of the linear replicons from *Nostoc* sp. N6. The alignment was generated using MUSCLE (Edgar, 2004) and visualized in JalView (Waterhouse et al., 2009).

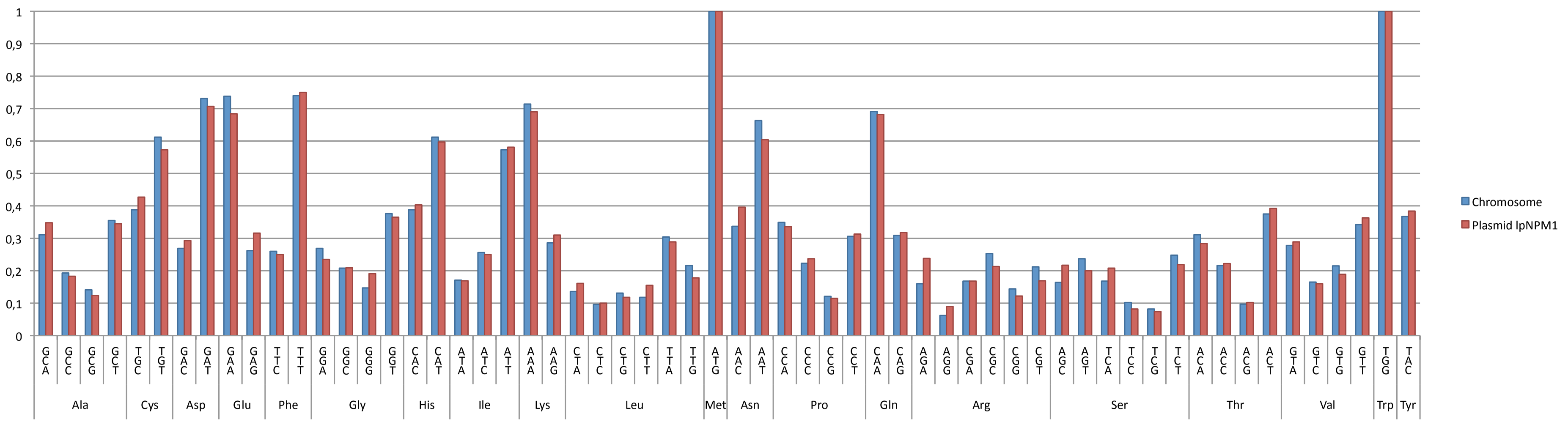

Figure S2. Codon usage of genes encoded on the *Nostoc* sp. N6 chromosome and linear replicon pNPM8. The ordinate axis shows the abundance of codon relative to sum of codons for that particular amino acid.

```
                                           dnaA                       |      oriC →
Nostoc sp. N6                AATCTCACTAGCCGTTCTCAAAAACCATCCTGAAGATGTATTTTCAGTTTTCCACAACCTGCACTGCGAT
Nostoc sp. 'L. pulmonaria'   AATATGAACAGCCGTTCTCAAAAACCATCCTGAAGATGTCTTTTAAGTTTTCCACAACCAGCAGTGCGAT
                              N  L  T  S  R  S  Q  K  P  S  *
                                 M

Nostoc sp. N6                CGCTAGGGTTGACGGCTAAATAATTAGTCGGAAATATTAAGTAACGTCTAAAAATTGATTAAATTTAACT
Nostoc sp. 'L. pulmonaria'   CGTTAGCCTTGACGGCTGAATAATTAGTCAAAAATATTAAGTAACGTATAAAATTTGATTAAATCTAACT

Nostoc sp. N6                TAAAGTGGGGAAAAAATCTTCCTTTCTGTGGATAAATCTGGAAAAAACTTGATATTTTGCTGATAAAACT
Nostoc sp. 'L. pulmonaria'   TAGATTGTGGAAAAA-TCTAACTTTTTGGGGAAAAATCTCTGAAAAACTTGAGATTTTGCTGACAAAACT

Nostoc sp. N6                TAATTAAGTATAATTACCTTGTGGAAAACTACATAAGTTTTTCCACAAGTTTTCCACAAG--GTAAACTT
Nostoc sp. 'L. pulmonaria'   TAATTAAGTATAATTACTTTGTGGAAAAGTACATAAGTTTTTCCACAAGTTTTCCACAGGTAGTTAACTT
```

Figure S3. Origins of replication of the *Nostoc* sp. N6 and *Nostoc* sp. '*Lobaria pulmonaria* cyanobiont' chromosomes. *dnaA* coding regions are highlighted with green, DnaA boxes are highlighted with yellow.

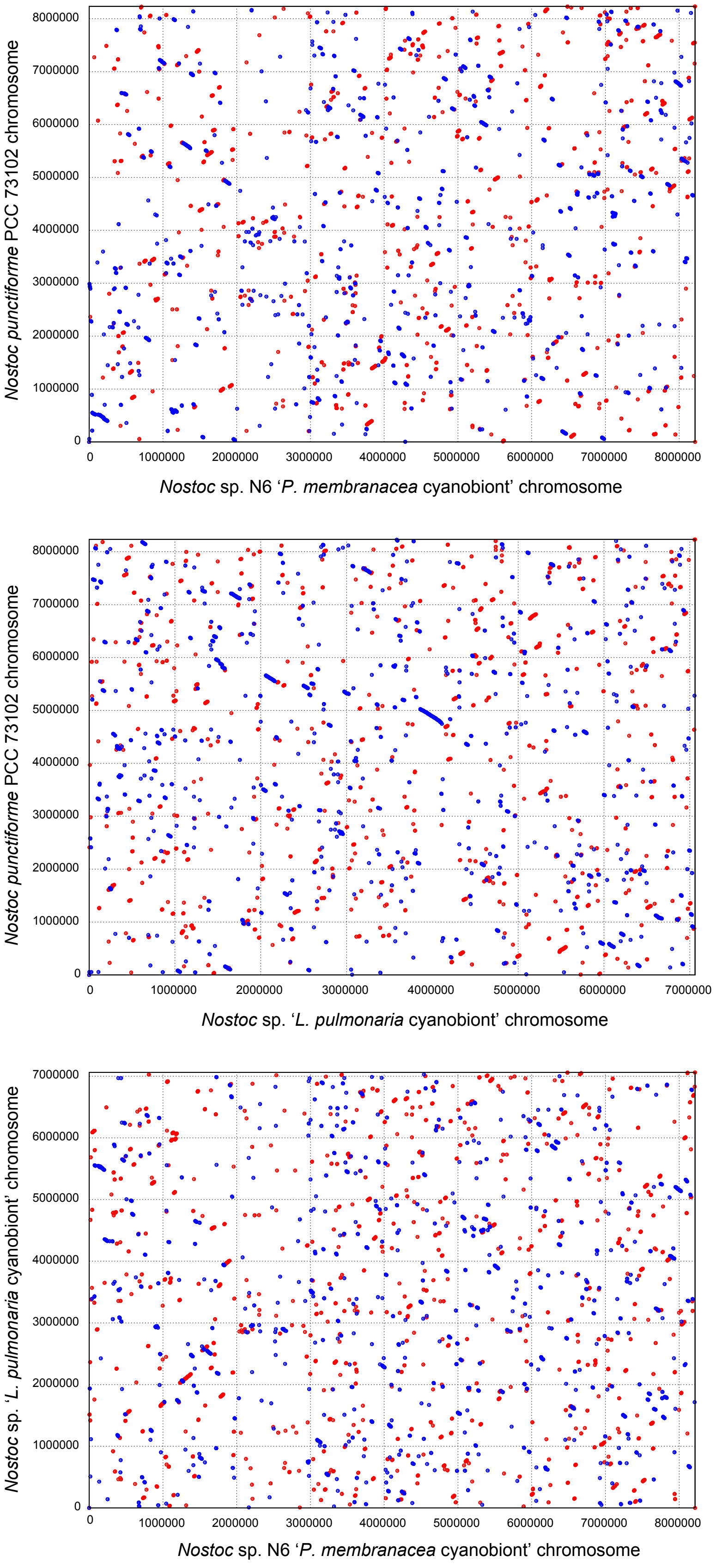

Figure S4. Dot plot comparisons of lichen-associated *Nostoc* strains and *N. punctiforme*. Dot plots were generated with PROmer (MUMmer 3.0 package; Kurtz et al., 2004). Only alignments that represent the best homology hit to any particular region on either sequence, i.e. a one-to-one mapping of reference and query subsequences, are displayed. Forward matches are shown in red and reverse matches are shown in blue.

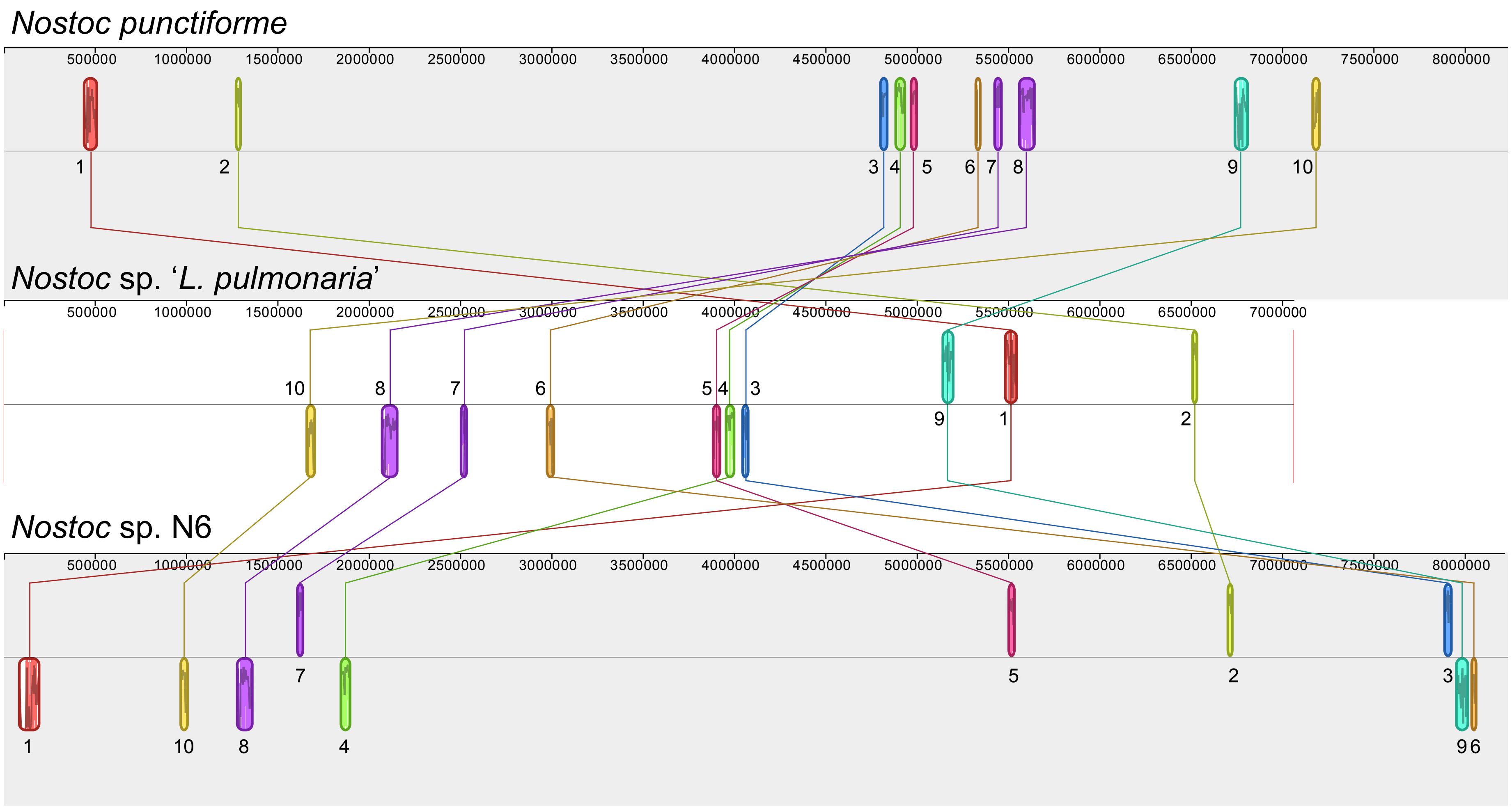

Figure S5. Genome sequence alignment of lichen-associated *Nostoc* strains and *N. punctiforme* (as a reference). Alignment was generated using Mauve (Darling et al., 2004). Only 10 most prominent locally collinear blocks (LCBs) are shown. For details on the proteins encoded in the LCBs see Supplementary Excel Table 1.

```
Nostoc sp. N6                      CGCGGGATAGAGCAGCCTGGTAGCTCGTCGGGCTCAACGGGCAAAGTTAACTACACGCTTATTAGTTAAGTTTGCTATGGGCGGCACGTAAAGAAACTTA
Nostoc sp. 210A                    CGCGGGATAGAGCAGCCTGGTAGCTCGTCGGGCTCA----------------------------------------------------------------
Nostoc sp. 213                     CGCGGGATAGAGCAGCCTGGTAGCTCGTCGGGCTCAACGGGCAAAGTTAACTACACGCTTATTAGTTAAGTTTGCTATGGGCGGCACGTAAAGAAACTTA
Nostoc sp. 232                     CGCGGGATAGAGCAGCCTGGTAGCTCGTCGGGCTCA----------------------------------------------------------------
Nostoc sp. 'Lobaria pulmonaria'    CGCGGGATAGAGCAGCCTGGTAGCTCGTCGGGCTCA----------------------------------------------------------------
Nostoc sp. 'Peltigera malacea'     CGCGGGATAGAGCAGCCTGGTAGCTCGTCGGGCTCA----------------------------------------------------------------
'P.membranacea' metagenome         CGCGGGATAGAGCAGCCTGGTAGCTCGTCGGGCTCAACGGGCAAAGTTAACTACACGCTTATTAGTTAAGTTTGCTATGGGCGGCACGTAAAGAAACTTA
Nostoc punctiforme                 CGCGGGATAGAGCAGCCTGGTAGCTCGTCGGGCTCA----------------------------------------------------------------
                                   ************************************

Nostoc sp. N6                      TGTGCGTTTATCTGTCAAACTCGGGGAAGCCAATAGCGCGGTAATCCCGAACCAAGCCTCAGAAATGAGGAAGGTGTAGAGACTGGAAGGCAGACACCCT
Nostoc sp. 210A                    ----------------------------------------------------------------------------------------------------
Nostoc sp. 213                     TGTGCGTTTATCTGTCAAACTCGGGGAAGCCAATAGCGCGGTAATCCCGAACCAAGCCTCAGAAATGAGGAAGGTGTAGAGACTGGAAGGCAGACACCCT
Nostoc sp. 232                     ----------------------------------------------------------------------------------------------------
Nostoc sp. 'Lobaria pulmonaria'    ----------------------------------------------------------------------------------------------------
Nostoc sp. 'Peltigera malacea'     ----------------------------------------------------------------------------------------------------
'P.membranacea' metagenome         TGTGCGTTTATCTGTCAAACTCGGGGAAGCCAATAGCGCGGTAATCCCGAACCAAGCCTCAGAAATGAGGAAGGTGTAGAGACTGGAAGGCAGACACCCT
Nostoc punctiforme                 ----------------------------------------------------------------------------------------------------

Nostoc sp. N6                      AACGATAATGACGAGGGTGAAGGGACAGTCCAGACCACAAACCAGTATAACTGGGCAACGAAAGTTGTAGTTGGTAAGCATAACCCGAAGGTCAGTGGTT
Nostoc sp. 210A                    --------------------------------------------------------------------------------TAACCCGAAGGTCAGTGGTT
Nostoc sp. 213                     AACGATAATGACGAGGGTGAAGGGACAGTCCAGACCACAAACCAGTATAACTGGGCAACGAAAGTTGTAGTTGGTAAGCATAACCCGAAGGTCAGTGGTT
Nostoc sp. 232                     --------------------------------------------------------------------------------TAACCCGAAGGTCAGTGGTT
Nostoc sp. 'Lobaria pulmonaria'    --------------------------------------------------------------------------------TAACCCGAAGGTCAGTGGTT
Nostoc sp. 'Peltigera malacea'     --------------------------------------------------------------------------------TAACCCGAAGGTCAGTGGTT
'P.membranacea' metagenome         AACGATAATGACGAGGGTGAAGGGACAGTCCAGACCACAAACCAGTATAACTGGGCAACGAAAGTTGTAGTTGGTAAGCATAACCCGAAGGTCAGTGGTT
Nostoc punctiforme                 --------------------------------------------------------------------------------TAACCCGAAGGTCAGTGGTT
                                                                                                                   ********************

Nostoc sp. N6                      CAAATCCACTTCCCGCCACCA
Nostoc sp. 210A                    CAAATCCACTTCCCGCCACCA
Nostoc sp. 213                     CAAATCCACTTCCCGCCACCA
Nostoc sp. 232                     CAAATCCACTTCCCGCCACCA
Nostoc sp. 'Lobaria pulmonaria'    CAAATCCACTTCCCGCCACCA
Nostoc sp. 'Peltigera malacea'     CAAATCCACTTCCCGCCACCA
'P.membranacea' metagenome         CAAATCCACTTCCCGCCACCA
Nostoc punctiforme                 CAAATCCACTTCCCGCCACCA
                                   *********************
```

Figure S6. Multiple alignment of $tRNA^{fMet}$ genes from symbiotic *Nostoc* strains. Group I introns are highlighted with green, anticodons are highlighted with yellow.

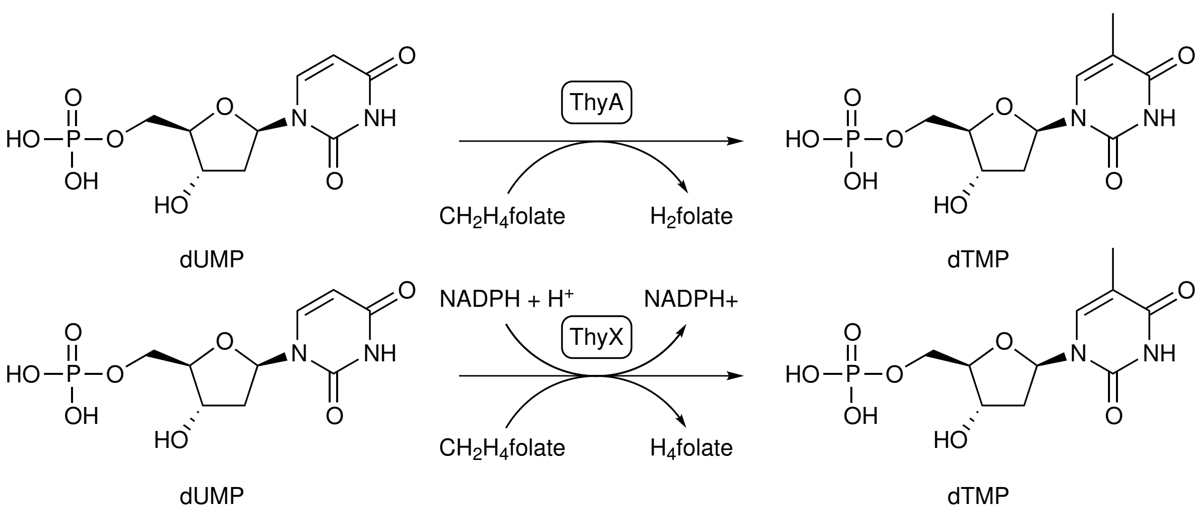

Figure S7. Reactions catalyzed by ThyA and ThyX thymidylate synthases. dUMP, deoxyuridine monophosphate; dTMP, thymidine monophosphate; CH_2H_4folate, 5,10-methylene tetrahydrofolate; H_2folate, dihydrofolate; H_4folate, tetrahydrofolate.

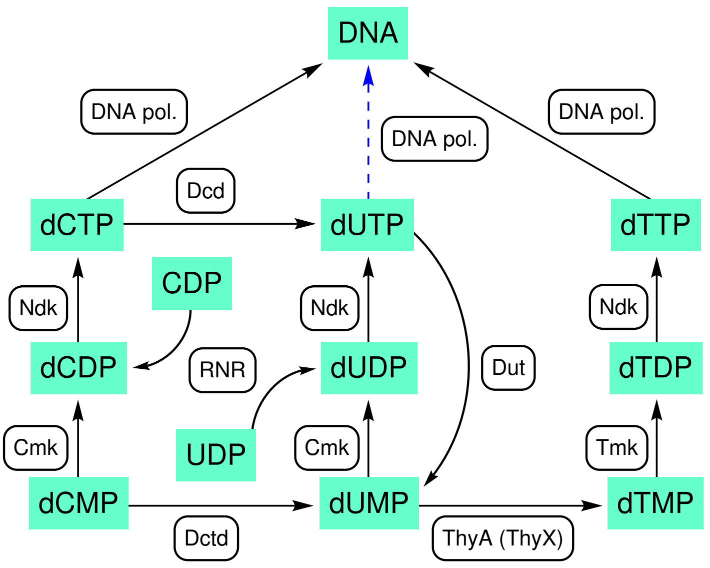

Figure S8. Simplified scheme of pyrimidine metabolism (KEGG pathway 00240). DNA pol., DNA polymerase; Dcd, dCTP deaminase; Ndk, nucleoside diphosphate kinase; Cmk, UMP/CMP kinase; Tmk, dTMP kinase; Dctd, dCMP deaminase; Dut, dUTP diphosphatase; ThyA (ThyX), thymidilate synthase; RNR, ribonucleoside-diphosphate reductase.

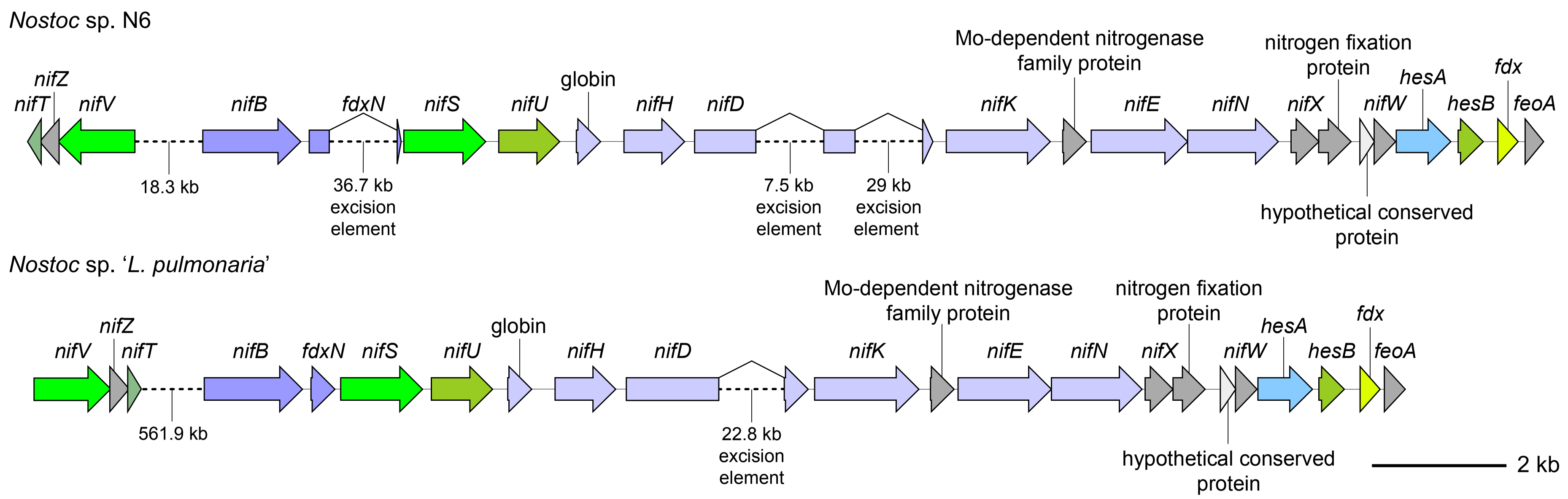

Figure S9. Arrangement of *nif* clusters in the *Nostoc* sp. N6 and *Nostoc* sp. '*Lobaria pulmonaria* cyanobiont' genomes. Insertion of excision elements in *fdxN* and *nifD* genes are indicated by dashed and connecting lines.

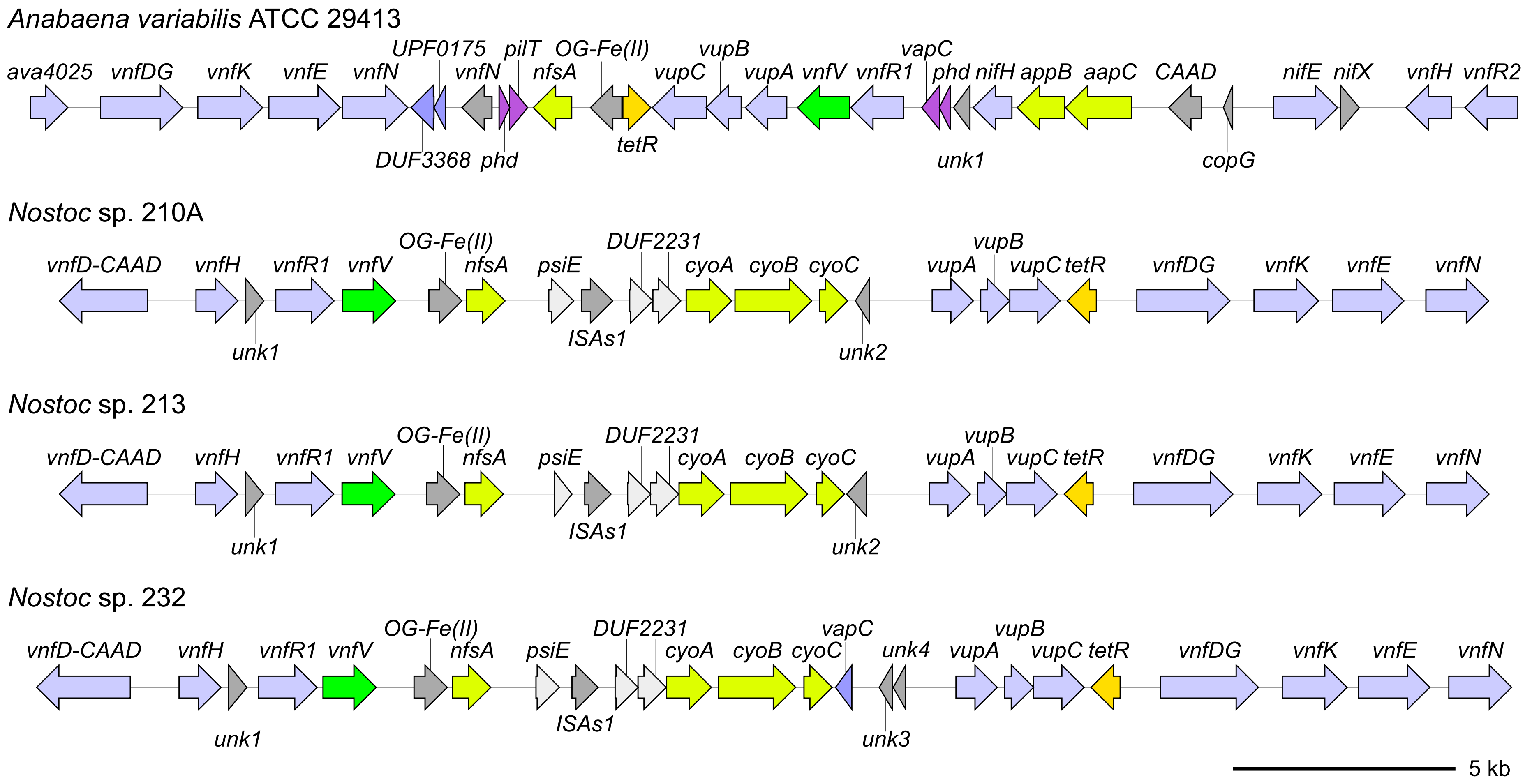

Figure S10. Arrangement of *vnf* clusters in *Nostoc* genomes. The *vnf* cluster from *Anabaena variabilis* is shown at the top for comparison. *unk*, conserved hypothetical proteins; UPF, uncharacterized protein family; DUF, domain of unknown function; OG-Fe(II), 2-oxoglutarate non-heme Fe(II) dependent oxidase; CAAD, cyanobacterial aminoacyl-tRNA synthetases appended domain (Ava_4049); *ISAs1*, ISAs1 family transposase. Homologous conserved hypothetical proteins are labeled with the same number, e.g. *unk1*.

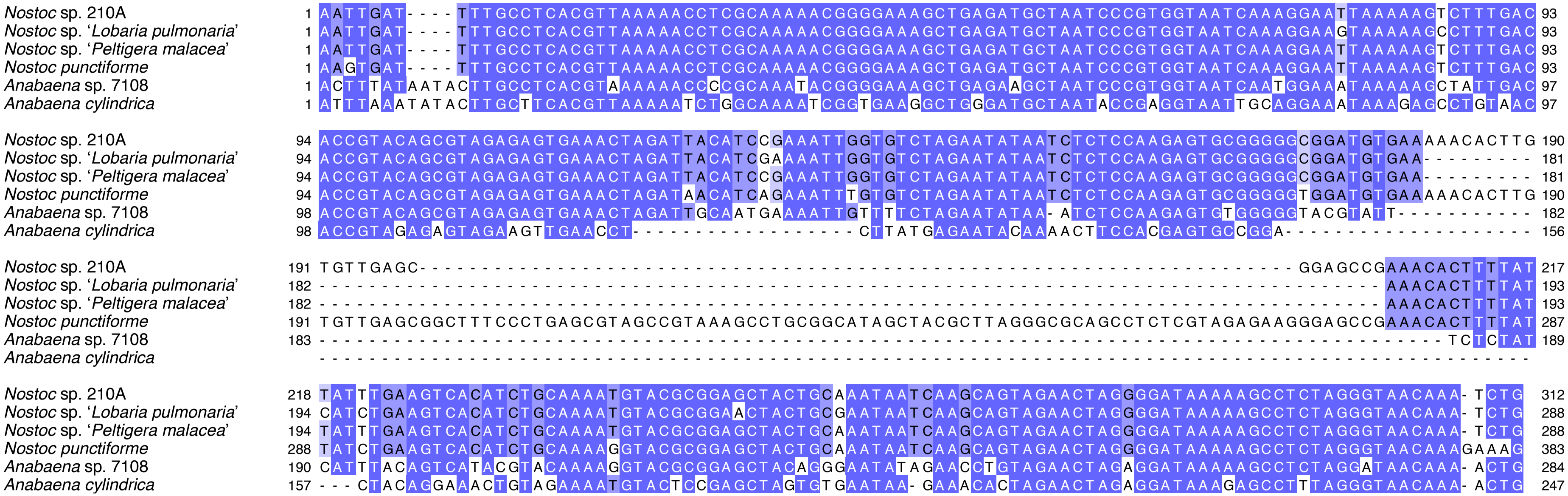

Figure S11. Multiple nucleotide sequence alignment of introns intervening the ribonucleoside-diphosphate reductase *nrdJ* genes in selected *Nostoc* and *Anabaena* strains. The alignment was generated using MUSCLE (Edgar, 2004) and visualized in JalView (Waterhouse et al., 2009).

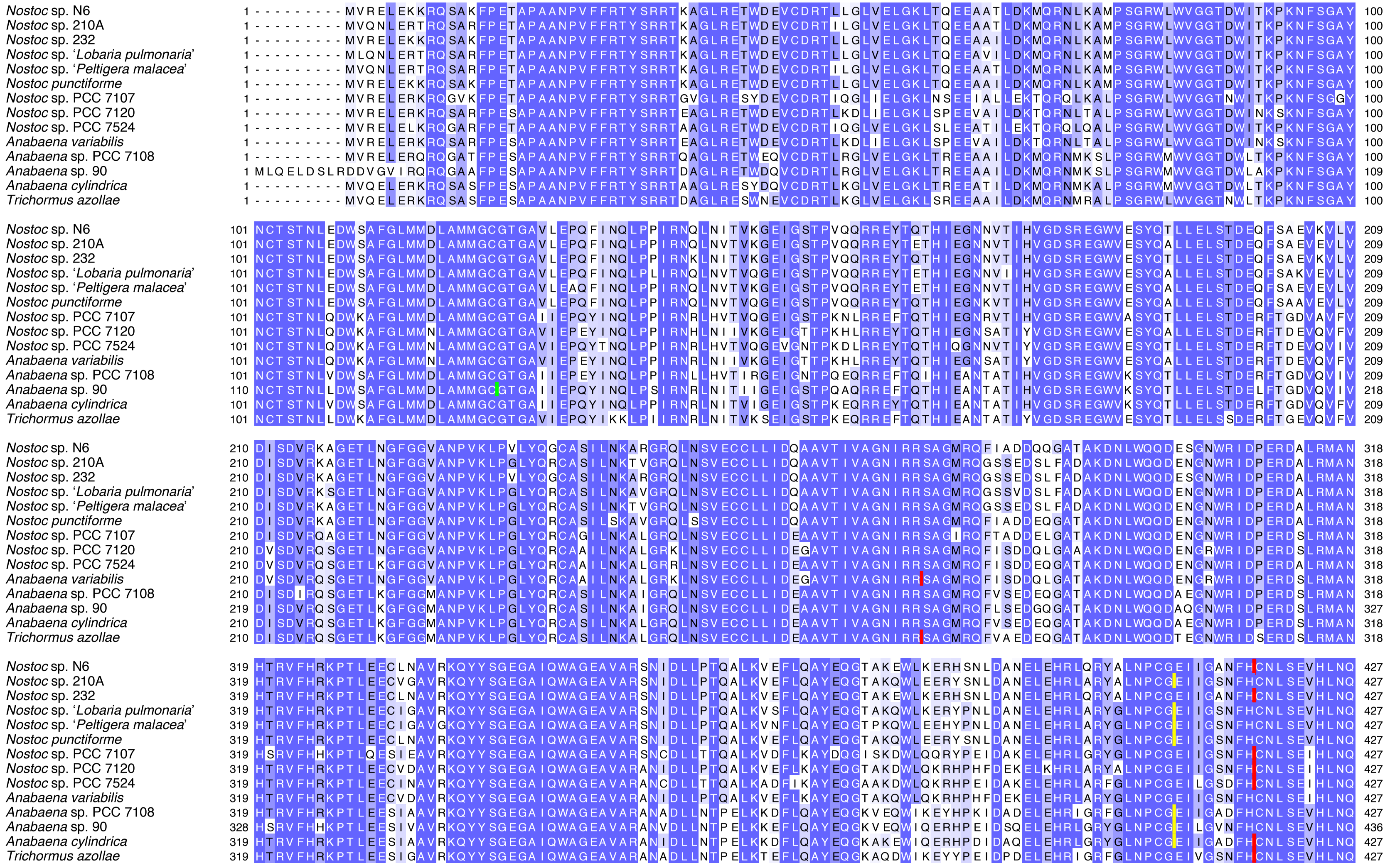

Figure S12. Multiple protein sequence alignment of NrdJ ribonucleoside-diphosphate reductases from selected ected *Nostoc* and *Anabaena* strains with *in silico* excised intervening sequences. Conserved sites of insertions are marked with red (inteins), yellow (group I introns) and green (group II intron). The alignment was generated using ClustalOmega (Sievers et al., 2011) and visualized in JalView (Waterhouse et al., 2009).

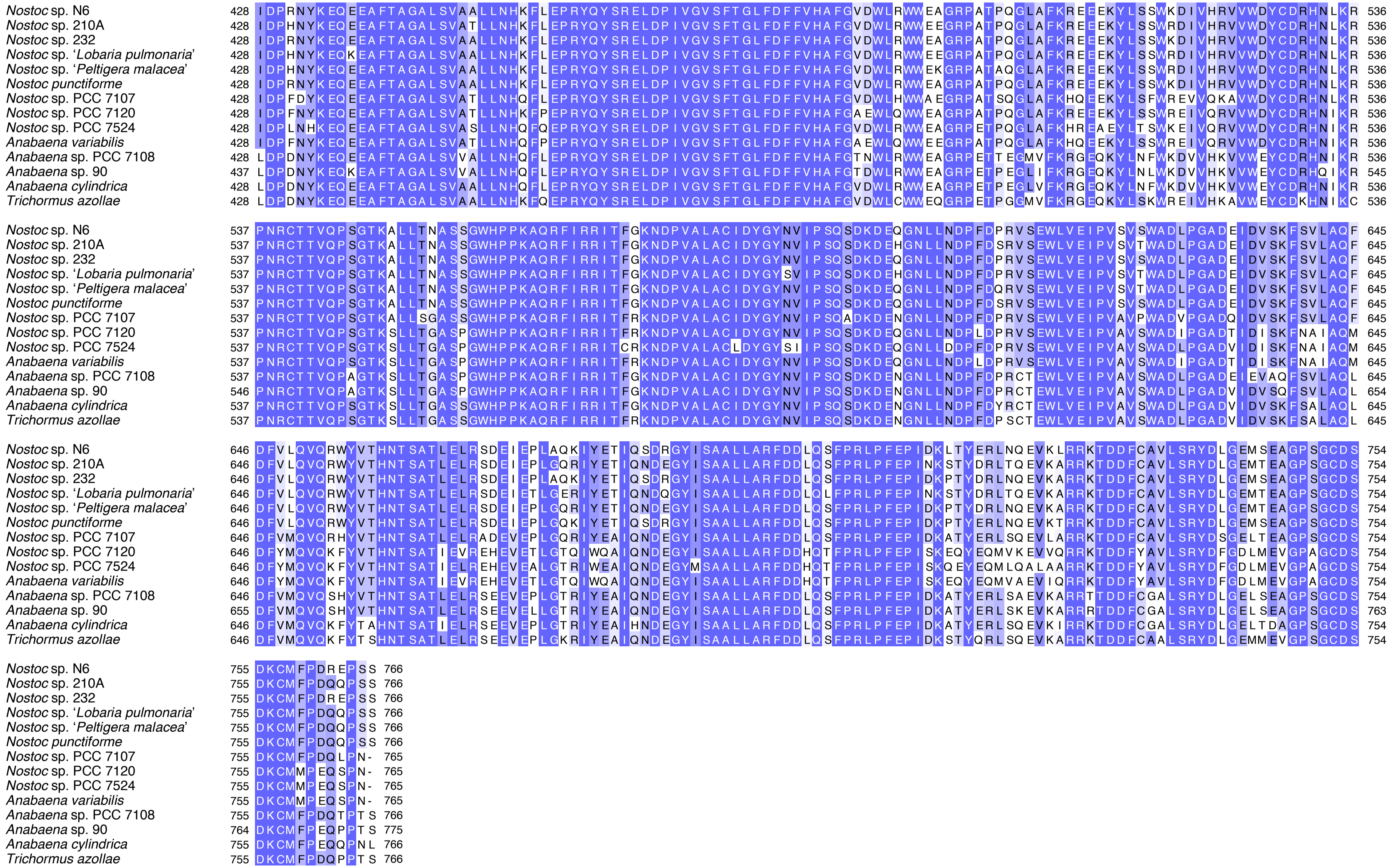

Figure S12. *Continued.*

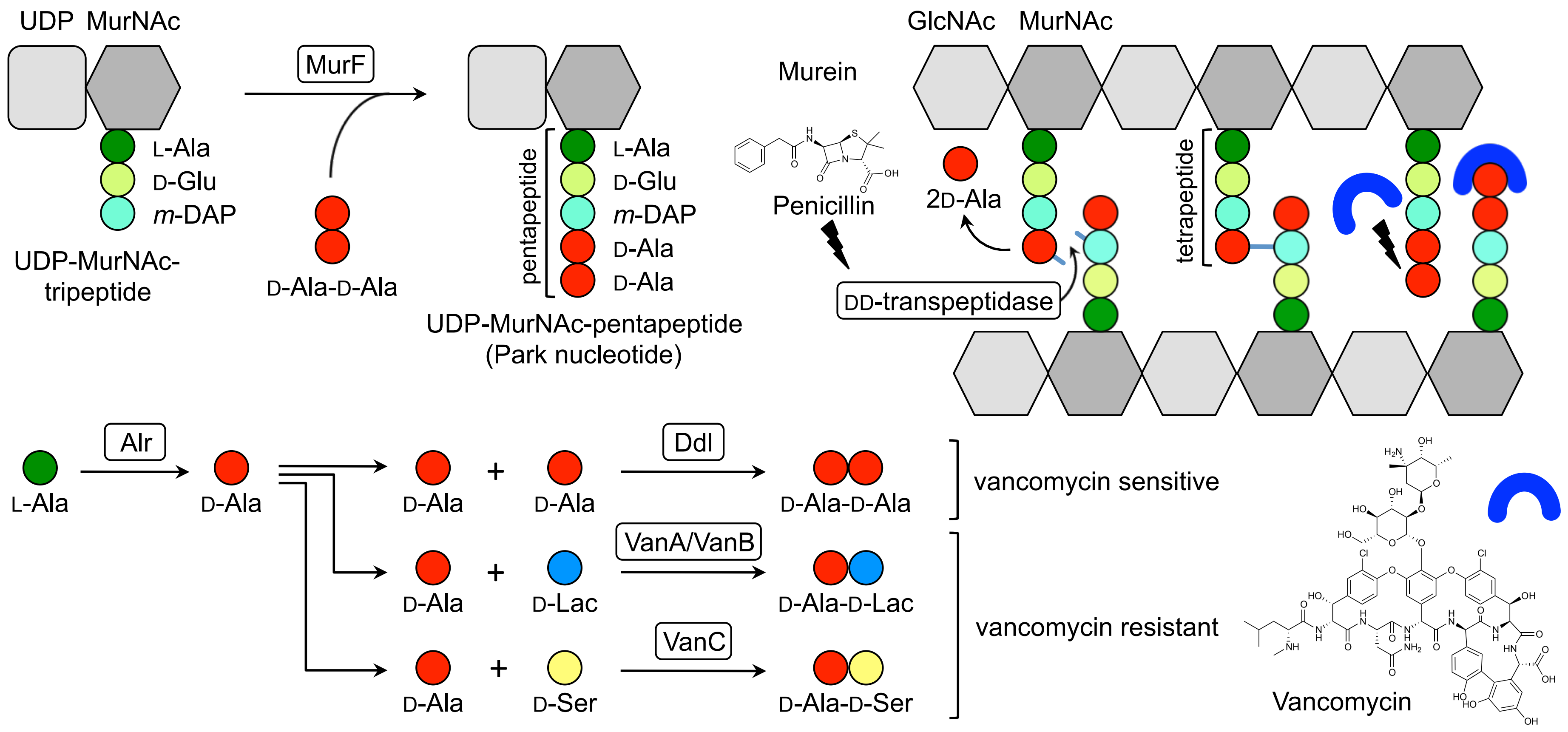

Figure S13. Simplified scheme of murein (DAP-type) biosynthesis. UDP, undecaprenyl moiety; MurNAc, *N*-acetylmuramoyl moiety; GlcNAc, *N*-acetylglucosamine moiety; *m*-DAP, *meso*-diaminopimelic acid. Key enzymes are shown boxed. MurF, UDP-*N*-acetylmuramoyl-tripeptide–D-alanyl-D-alanine ligase; DD-transpeptidase, D-Ala-D-Ala transpeptidase; Alr, alanine racemase; Ddl, D-alanine-D-alanine ligase; VanA/VanB, D-alanine-D-lactate ligase; VanC, D-alanine-D-serine ligase.

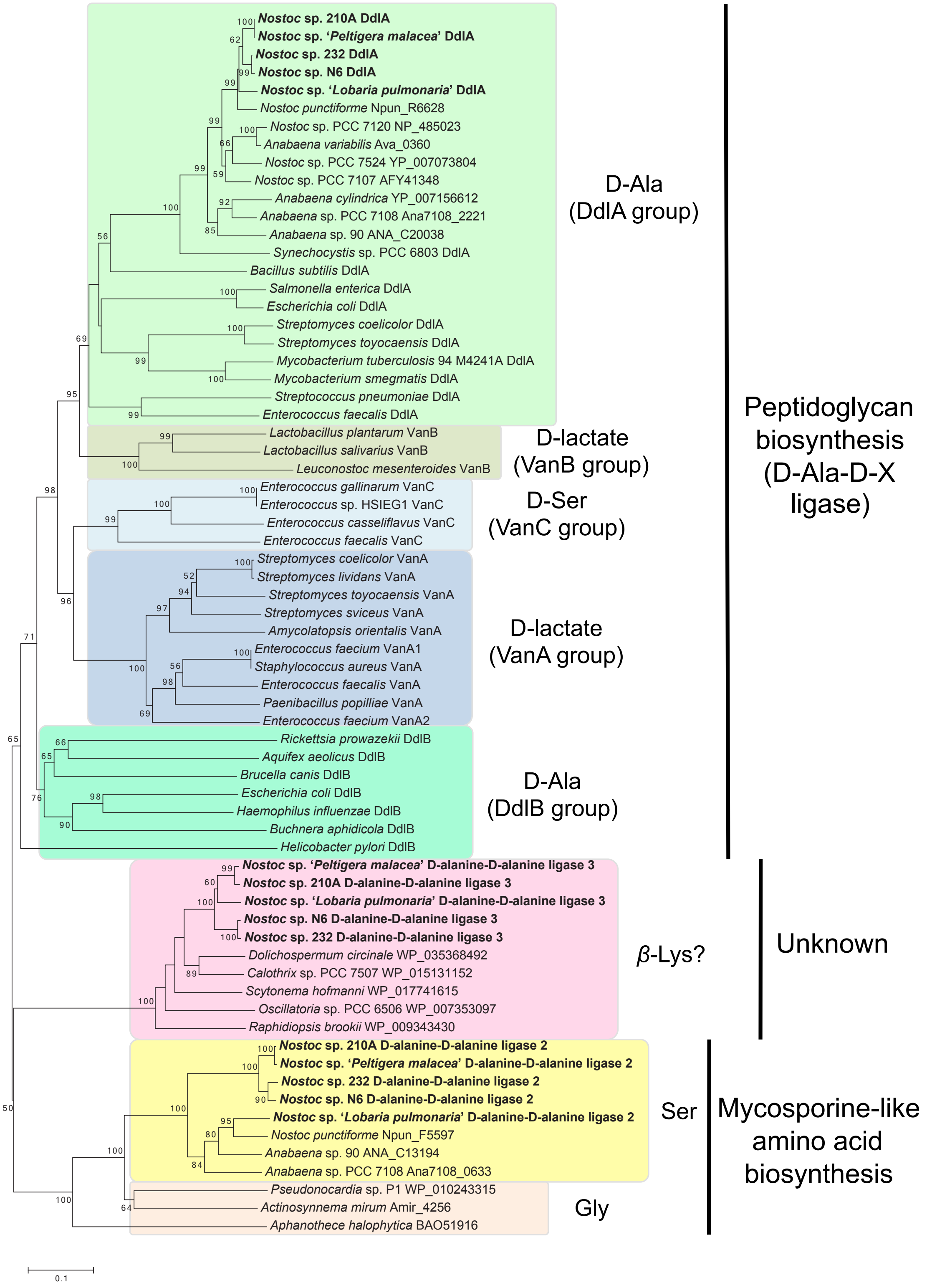

Figure S14. Neighbor-joining phylogenetic tree based on the protein sequences of selected D-Ala-D-Ala ATP grasp ligases. The enzymes found in lichen-associated *Nostoc* strains are shown in bold). Numbers at branch nodes are bootstrap percentages (based on 1000 resamplings). Substrates and proposed functions are shown on the right. Bar, 10% sequence divergence.

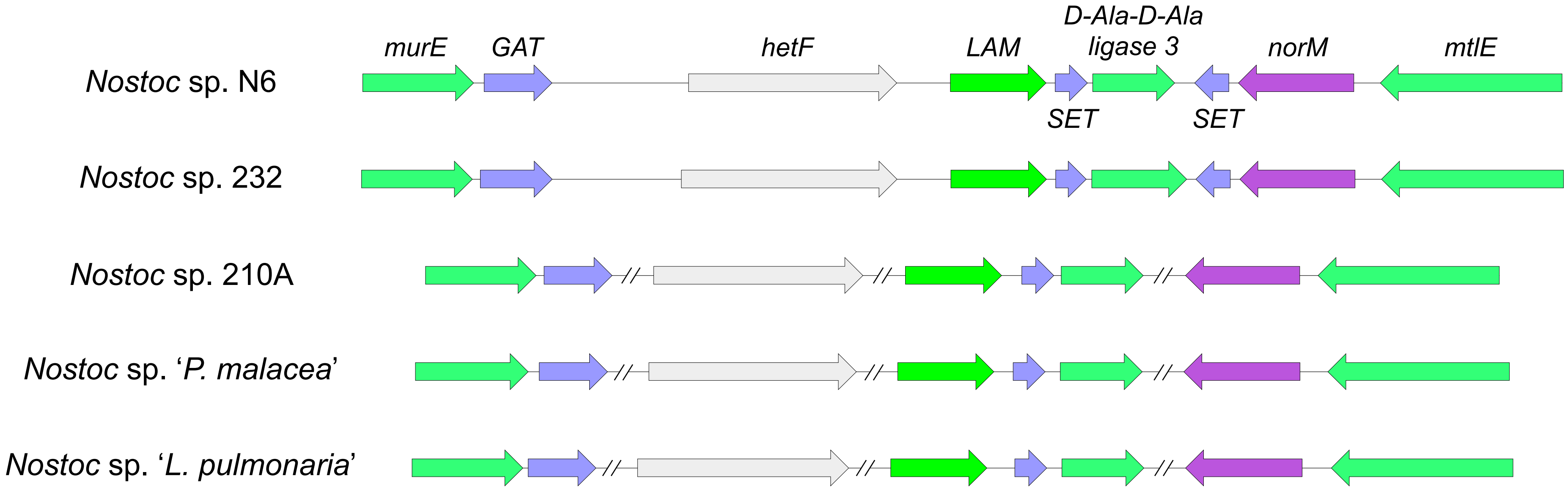

Figure S15. D-Ala-D-Ala ligase 3 gene clusters of lichen-associated *Nostoc* strains. Gene abbreviations: *murF*, UDP-*N*-acetylmuramoylalanyl-D-glutamate–2,6-diaminopimelate ligase; *GAT*, CobQ-like glutamine amidotransferase; *hetF*, heterocyst differentiation protein; *LAM*, lysine 2,3-aminomutase; *SET*, lysine methyltransferase; *norM*, MATE family efflux pump; *mtlE*, soluble lytic murein transglycosylase. A broken genome line indicates separate loci.

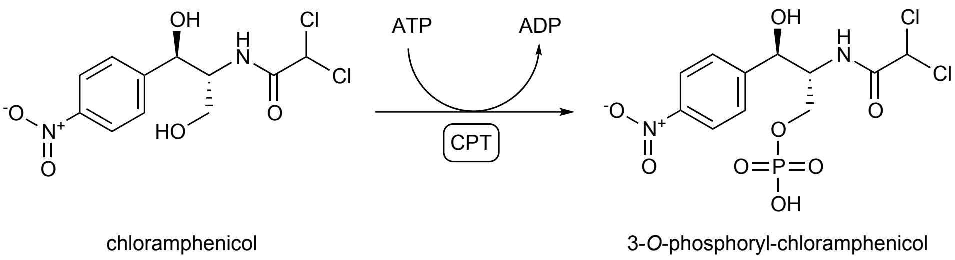

Figure S16. Phosphorylation of chloramphenicol by chloramphenicol phosphotransferase (CPT).

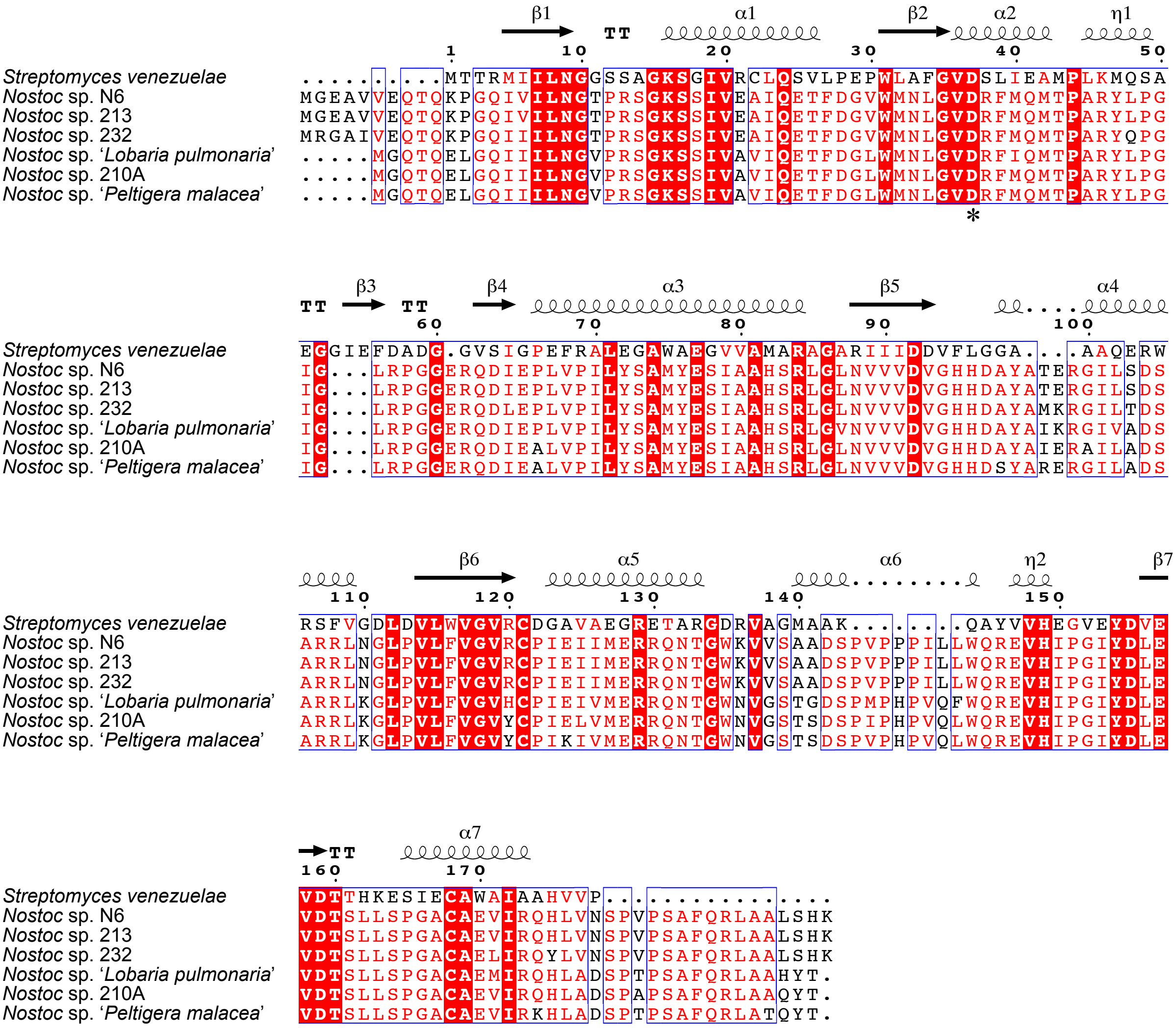

Figure S17. Protein sequence alignment of chloramphenicol phosphotransferases (CPTs) from the chloramphenicol producer *Streptomyces venezuelae* and lichen-associated *Nostoc* strains. The secondary structure information and numbering are displayed for the *S. venezuelae* CPT (PDB accession 1QHY; Izard and Ellis, 2000). The conserved catalytic aspartic acid residue (Asp37) is indicated with an asterisk. The alignment was generated with ESPript 3 (Robert and Gouet, 2014).

Nostoc sp. N6 *nos*

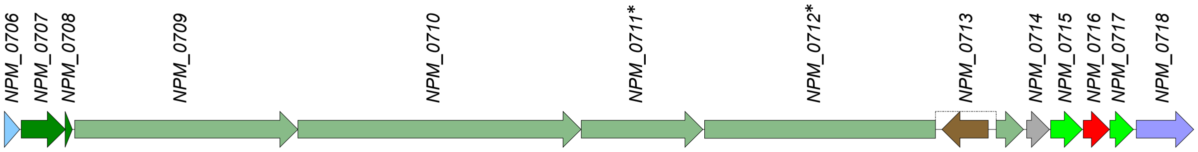

ORF	Protein size, aa	Proposed function	Closest homolog (origin)	% identity	GenBank accession number
NPM_0706	179	4-hydroxybenzoate synthetase	*Nostoc* sp. NIES-3756	85	BAT53516
NPM_0707	513	AMP-dependent synthetase/ligase	*Nostoc* sp. NIES-3756	84	BAT53517
NPM_0708	83	acyl carrier protein	*Nostoc* sp. NIES-3756	78	BAT53518
NPM_0709	2603	NRPS[a]	*Nostoc* sp. '*Peltigera membranacea* cyanobiont'	99	ADL59761
NPM_0710	3302	NRPS[b]	*Nostoc* sp. '*P. membranacea* cyanobiont'	99	ADL59762
NPM_0711*	1418[c]	PKS[d]	*Nostoc* sp. '*P. membranacea* cyanobiont'	99	ADL59763
NPM_0712*	3474[e]	NRPS[f]	*Nostoc* sp. '*P. membranacea* cyanobiont'	99	ADL59764
NPM_0713	541	*IS1634* transposase	*Calothrix* sp. PCC 6303	78	AFZ00424
NPM_0714	265	hypothetical protein	*Nostoc* sp. '*P. membranacea* cyanobiont'	99	ADL59765
NPM_0715	367	alcohol dehydrogenase	*Nostoc* sp. '*P. membranacea* cyanobiont'	100	ADL59766
NPM_0716	303	JmjC domain protein	*Nostoc* sp. '*P. membranacea* cyanobiont'	99	ADL59767
NPM_0717	273	pyrroline-5-carboxylate reductase	*Nostoc* sp. '*P. membranacea* cyanobiont'	99	ADL59768
NPM_0718	669	ABC transporter	*Nostoc* sp. '*P. membranacea* cyanobiont'	99	ADL59769

[a] Deduced architecture of the encoded NRPS protein is C–A–PCP–C–A–MeT–PCP.
[b] Deduced architecture of the encoded NRPS protein is C–A–PCP–C–A–PCP–C–A–PCP.
[c] The size of the closest homolog (ADL59763).
[d] Deduced architecture of the encoded PKS protein is KS–AT–MeT–ACP.
[e] The size of the closest homolog (ADL59764).
[f] Deduced architecture of the encoded NRPS protein is C–A–PCP–C–A–PCP–E–C–PCP–TE.

Figure S18. Gene clusters encoding glycolipid synthases (Hgl), polyketide synthases (PKSs) and non-ribosomal peptide synthases (NRPSs) in the *Nostoc* sp. N6 and *Nostoc* sp. '*Lobaria pulmonaria* cyanobiont' genomes. KS, β-ketoacyl synthase; KS^0, nonelongating KS; AT, acyltransferase; DH, dehydratase; ER, enoyl reductase; KR, ketoreductase; ACP, acyl carrier protein; TE, thioesterase; MeT, methyltransferase; CR, crotonase (enoyl-CoA hydratase); C, condensation domain; A, adenylation domain; PCP, peptidyl carrier protein; AS, acyl-CoA synthetase (fatty acyl-AMP ligase); E, epimerization domain; GA, glyceric acid tranferase; S, sulfotransferase; GNAT, GCN5-related *N*-acetyltransferase family; R, *C*-terminal reductase; ?, unknown. Pseudogenes are denoted with an asterisk (*).

Nostoc sp. N6 *ncp*

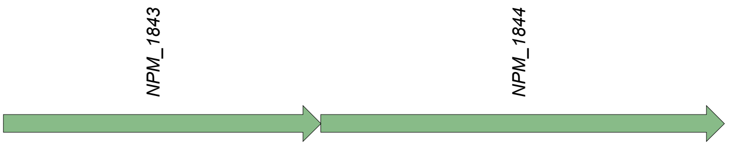

ORF	Protein size, aa	Proposed function	Closest homolog (origin)	% identity	GenBank accession number
NPM_1843	3705	NRPS[a]	*Nostoc* sp. ATCC 53789	94	AAO23333
NPM_1844	4710	NRPS[b]	*Nostoc* sp. ATCC 53789	81	AAO23334

[a] Deduced architecture of the encoded NRPS protein is C–A–PCP–C–A–PCP–C–A–PCP–E.
[b] Deduced architecture of the encoded NRPS protein is C–A–PCP–C–A–PCP–C–A–PCP–C–A–PCP–R.

Nostoc sp. N6 *nsp*

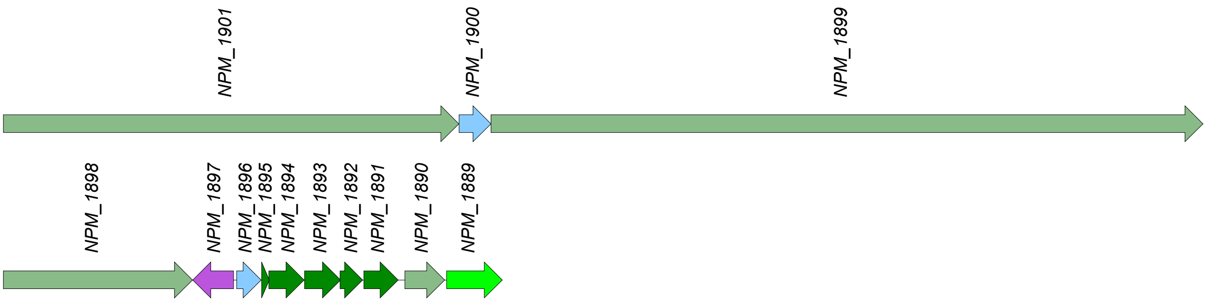

ORF	Protein size, aa	Proposed function	Closest homolog (origin)	% identity	GenBank accession number
NPM_1889 (*nspM*)	647	asparagine synthase	*Nostoc* sp. '*Peltigera membranacea* cyanobiont'	99	ADA69249
NPM_1890 (*nspL*)	464	cytochrome P450	*Nostoc* sp. '*P. membranacea* cyanobiont'	100	ADA69248
NPM_1891 (*nspK*)	400	acyltransferase	*Nostoc* sp. '*P. membranacea* cyanobiont'	99	ADA69247
NPM_1892 (*nspJ*)	262	enoyl-CoA hydratase	*Nostoc* sp. '*P. membranacea* cyanobiont'	100	ADA69246
NPM_1893 (*nspI*)	420	3-hydroxy-3-methyl-glutaryl-ACP synthase	*Nostoc* sp. '*P. membranacea* cyanobiont'	100	ADA69245
NPM_1894 (*nspH*)	411	β-ketoacyl synthase	*Nostoc* sp. '*P. membranacea* cyanobiont'	100	ADA69244
NPM_1895 (*nspG*)	90	acyl carrier protein	*Nostoc* sp. '*P. membranacea* cyanobiont'	100	ADA71314
NPM_1896 (*nspF*)	285	*O*-methyltrasferase	*Nostoc* sp. '*P. membranacea* cyanobiont'	99	ADA69243
NPM_1897 (*nspE*)	474	MATE family efflux transporter	*Nostoc* sp. '*P. membranacea* cyanobiont'	99	ADA69242
NPM_1898 (*nspD*)	2206	PKS[a]	*Nostoc* sp. '*P. membranacea* cyanobiont'	98	ADA69241
NPM_1899 (*nspC*)	8304	*trans*-AT PKS-NRPS[b]	*Nostoc* sp. '*P. membranacea* cyanobiont'	97	ADA69239
NPM_1900 (*nspB*)	371	luciferase-like monooxygenase	*Nostoc* sp. '*P. membranacea* cyanobiont'	99	ADA69238
NPM_1901 (*nspA*)	5320	*trans*-AT PKS[c]	*Nostoc* sp. '*P. membranacea* cyanobiont'	98	ADA69237

[a] Deduced architecture of the encoded PKS protein is KS–AT–KR–ER–ACP–TE.
[b] Deduced architecture of the encoded PKS-NRPS protein is KS^0–ACP–C–A–PCP–KS–DH–KR–MeT–ACP–KS^0–DH–ACP–KS–KR–MeT–ACP–KS^0–ACP–C–A–PCP.
[c] Deduced architecture of the encoded PKS protein is GNAT–ACP–KS–KR–MeT–ACP–KS–CR–CR–ACP–ACP–KS–?–KR–ACP–ACP.

Figure S18. *Continued.*

Nostoc sp. N6 *nrps1*

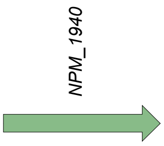

ORF	Protein size, aa	Proposed function	Closest homolog (origin)	% identity	GenBank accession number
NPM_1940	1826	NRPS[a]	*Nostoc* sp. ATCC 53789	82	AAO23333

[a] Deduced architecture of the encoded NRPS protein is C–A–PCP–C–A (partial).

Nostoc sp. N6 *nrps2*

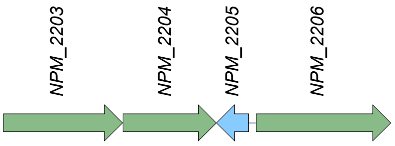

ORF	Protein size, aa	Proposed function	Closest homolog (origin)	% identity	GenBank accession number
NPM_2203	1390	NRPS[a]	*Scytonema hofmanni* UTEX 2349	88	WP_029631596
NPM_2204	1090	NRPS[b]	*S. hofmanni* UTEX 2349	91	WP_029631594
NPM_2205	375	α/β hydrolase	*S. hofmanni* UTEX 2349	94	WP_029631593
NPM_2206	1568	NRPS[c]	*S. hofmanni* PCC 7110	88	WP_051077115

[a] Deduced architecture of the encoded NRPS protein is C–A–PCP–TE.
[b] Deduced architecture of the encoded NRPS protein is C–A–PCP.
[c] Deduced architecture of the encoded NRPS protein is C–A–MeT–PCP.

Figure S18. *Continued.*

Nostoc sp. N6 *nrps3*

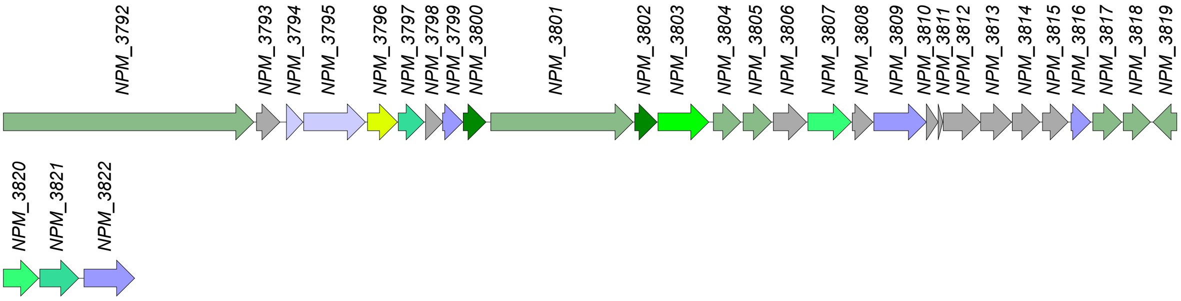

ORF	Protein size, aa	Proposed function	Closest homolog (origin)	% identity	GenBank accession number
NPM_3792	2922	NRPS[a]	*Planktothrix agardhii* NIES-205	67	ABW84363
NPM_3793	274	hypothetical protein	*P. agardhii* NIVA-CYA 126	67	CAM59602
NPM_3794	192	adenylyl-sulfate kinase	*Nostoc punctiforme* PCC 73102	77	ACC83034
NPM_3795	720	(2Fe-2S) ferredoxin	*P. prolifica* NIVA-CYA 98	57	CAQ48268
NPM_3796	351	isopentenyl pyrophosphate isomerase	*Nodularia spumigena* CCY9414	86	EAW46435
NPM_3797	301	permease	*Mastigocladus laminosus* UU774	52	KIY09608
NPM_3798	202	bacilysin biosynthesis protein BacA	*P. agardhii* NIVA-CYA 126	61	CAM59603
NPM_3799	235	cupin	*P. rubescens* NIVA-CYA 407	53	WP_026785726
NPM_3800	264	short-chain dehydrogenase/ reductase	*P. agardhii* NIVA-CYA 126	73	CAM59605
NPM_3801	1658	NRPS[b]	*Moorea producens* JHB	63	AKV71859
NPM_3802	258	short-chain dehydrogenase/ reductase	*M. producens* JHB	83	AKV71860
NPM_3803	595	peptide ABC transporter substrate-binding protein	*Crinalium epipsammum* PCC 9333	57	AFZ13263
NPM_3804	320	2OG-Fe(II) oxygenase	*P. prolifica* NIVA-CYA 98	40	CAQ48273
NPM_3805	329	2OG-Fe(II) oxygenase	*Myxosarcina* sp. GI1	39	WP_052055871
NPM_3806	385	hypothetical protein	*Dolichospermum circinale* AWQC310F	59	WP_035371543
NPM_3807	506	D-alanyl-lipoteichoic acid acyltransferase DltB	*M. producens* 3L	67	EGJ31355
NPM_3808	240	hypothetical protein	*Bacillus methanolicus* MGA3	50	EIJ77741
NPM_3809	612	carbamoyltransferase	*Aphanizomenon flos-aquae* NH-5	84	ACG63816
NPM_3810	135	hypothetical protein	*A. flos-aquae* NH-5	60	ACG63817
NPM_3811	54	hypothetical protein	*Arthrospira maxima* CS-328	69	EDZ96930
NPM_3812	430	GDSL-like lipase	*A. flos-aquae* NH-5	52	ACG63819
NPM_3813	362	GDSL-like lipase	candidate division TA06 bacterium DG_24	24	KPJ53805
NPM_3814	321	hypothetical protein	Oscillatoriales cyanobacterium MTP1	56	WP_058881380
NPM_3815	299		Oscillatoriales cyanobacterium MTP1	60	WP_058881380
NPM_3816	227	radical SAM protein	*Beggiatoa* sp. PS	50	EDN71334
NPM_3817	335	2OG-Fe(II) oxygenase	*Gloeocapsa* sp. PCC 7428	43	AFZ31754
NPM_3818	317	2OG-Fe(II) oxygenase	*Fischerella* sp. PCC 9605	41	WP_026731186
NPM_3819	277	aldo/keto reductase	*Leptolyngbya* sp. JSC-1	68	WP_036005646
NPM_3820	413	glycosyltransferase	*P. agardhii* NIVA-CYA 126	55	CAM59608
NPM_3821	454	MFS transporter	*D. circinale* AWQC310F	57	WP_051289538
NPM_3822	590	ABC transporter	*Nostoc punctiforme* PCC 73102	78	ACC81026

[a] Deduced architecture of the encoded NRPS protein is GA–ACP–S–C–A–PCP–E.
[b] Deduced architecture of the encoded NRPS protein is C–A–PCP–C–PCP.

Figure S18. *Continued.*

Nostoc sp. N6 *nrps4*

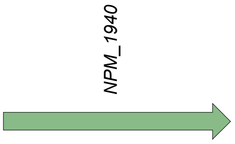

ORF	Protein size, aa	Proposed function	Closest homolog (origin)	% identity	GenBank accession number
NPM_4088	2652	NRPS[a]	*Geitlerinema* sp. PCC 7105	54	WP_017663026

[a] Deduced architecture of the encoded NRPS protein is C–A–PCP–C–A–PCP–R.

Nostoc sp. N6 *pks1*

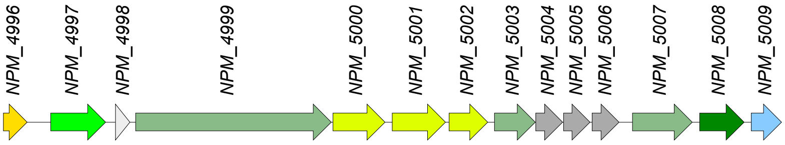

ORF	Protein size, aa	Proposed function	Closest homolog (origin)	% identity	GenBank accession number
NPM_4996	278	DNA-binding response regulator	*Tolypothrix campylonemoides* VB511288	69	KIJ79159
NPM_4997	641	asparagine synthetase B	*Microcoleus* sp. PCC 7113	67	AFZ22237
NPM_4998	166	protein of unknown function (DUF302)	*Fischerella muscicola* PCC 7414	60	WP_016860191
NPM_4999	2281	PKS[a]	*Nostoc* sp. '*Peltigera membranacea* cyanobiont'	99	AGK38335
NPM_5000	606	geranylgeranyl reductase family protein	*Nostoc* sp. '*P. membranacea* cyanobiont'	99	AGK38334
NPM_5001	623	geranylgeranyl reductase family protein	*Nostoc* sp. '*P. membranacea* cyanobiont'	99	AGK38336
NPM_5002	451	aldehyde dehydrogenase	*Nostoc* sp. '*P. membranacea* cyanobiont'	97	AGK38337
NPM_5003	475	9-*cis*-epoxycarotenoid dioxygenase	*Nostoc* sp. '*P. membranacea* cyanobiont'	99	AGK38338
NPM_5004	313	protein of unknown function (DUF1702)	*Nostoc* sp. '*P. membranacea* cyanobiont'	99	AGK38339
NPM_5005	307	protein of unknown function (DUF1702)	*Nostoc* sp. '*P. membranacea* cyanobiont'	97	AGK38340
NPM_5006	311	protein of unknown function (DUF1702)	*Nostoc* sp. '*P. membranacea* cyanobiont'	99	AGK38341
NPM_5007	695	hemolysin-type calcium-binding protein	*Nostoc* sp. '*P. membranacea* cyanobiont'	99	AGK38342
NPM_5008	517	cholesterol oxidase	*Nostoc* sp. '*P. membranacea* cyanobiont'	96	AGK38343
NPM_5009	352	prenyltransferase UbiA	*Nostoc* sp. '*P. membranacea* cyanobiont'	99	AGK38344

[a] Deduced architecture of the encoded PKS protein is KS–AT–ACP–ACP–KR–DH.

Figure S18. *Continued.*

Nostoc sp. N6 *nrps5*

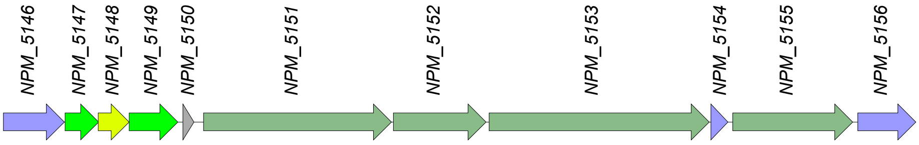

ORF	Protein size, aa	Proposed function	Closest homolog (origin)	% identity	GenBank accession number
NPM_5146	711	patatin	*Mastigocladus laminosus* UU774	68	KIY11706
NPM_5147	392	2-isopropylmalate synthase	*Nostoc punctiforme* PCC 73102	80	ACC81025
NPM_5148	360	3-isopropylmalate dehydrogenase	*N. punctiforme* PCC 73102	89	ACC81018
NPM_5149	569	3-isopropylmalate dehydratase large subunit	*N. punctiforme* PCC 73102	88	ACC81019
NPM_5150	130	hypothetical protein	*Nostoc* sp. '*Peltigera membranacea* cyanobiont'	74	AGH69799
NPM_5151	2191	NRPS[a]	*Scytonema hofmanni* PCC 7110	78	WP_017742642
NPM_5152	1079	NRPS[b]	*N. punctiforme* PCC 73102	81	ACC81022
NPM_5153	2566	NRPS[c]	*N. punctiforme* PCC 73102	79	ACC81023
NPM_5154	198	protein of unknown function (DUF820)	*Microcystis aeruginosa* PCC 9806	76	CCI16695
NPM_5155	1401	NRPS[d]	*N. punctiforme* PCC 73102	81	ACC81024
NPM_5156	677	ABC transporter	*N. punctiforme* PCC 73102	80	ACC81026

[a] Deduced architecture of the encoded NRPS protein is A–PCP–C–A–PCP–E.
[b] Deduced architecture of the encoded NRPS protein is C–A–PCP.
[c] Deduced architecture of the encoded NRPS protein is C–A–PCP–C–A–MeT–PCP.
[d] Deduced architecture of the encoded NRPS protein is C–A–PCP–TE.

Nostoc sp. N6 *hgl*

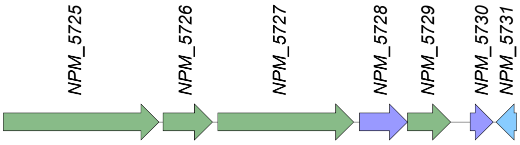

ORF	Protein size, aa	Proposed function	Closest homolog (origin)	% identity	GenBank accession number
NPM_5725	1814	heterocyst glycolipid synthase HglE[a]	*Nostoc* sp. '*Peltigera membranacea* cyanobiont'	99	AGJ76601
NPM_5726	575	heterocyst glycolipid ketoreductase HglG	*Nostoc* sp. '*P. membranacea* cyanobiont'	99	AGJ76602
NPM_5727	1582	heterocyst glycolipid synthase HglC[b]	*Nostoc* sp. '*P. membranacea* cyanobiont'	98	AGJ76603
NPM_5728	558	heterocyst glycolipid enoyl reductase PfaD (2-nitropropane dioxygenase)	*Nostoc* sp. '*P. membranacea* cyanobiont'	99	AGJ76604
NPM_5729	504	heterocyst glycolipid synthase HglB[c]	*Nostoc* sp. '*P. membranacea* cyanobiont'	100	AGJ76605
NPM_5730	267	short-chain dehydrogenase/reductase	*Nostoc* sp. '*P. membranacea* cyanobiont'	100	AGJ76606
NPM_5731	236	4′-phosphopantetheinyl transferase	*Nostoc* sp. '*P. membranacea* cyanobiont'	99	AGJ76607

[a] Deduced architecture of the encoded protein is KS–MAT–ACP–ACP–KR.
[b] Deduced architecture of the encoded protein is KS–CLF–AT.
[c] Deduced architecture of the encoded protein is ACP–TE.

Figure S18. *Continued.*

Nostoc sp. N6 *pks2*

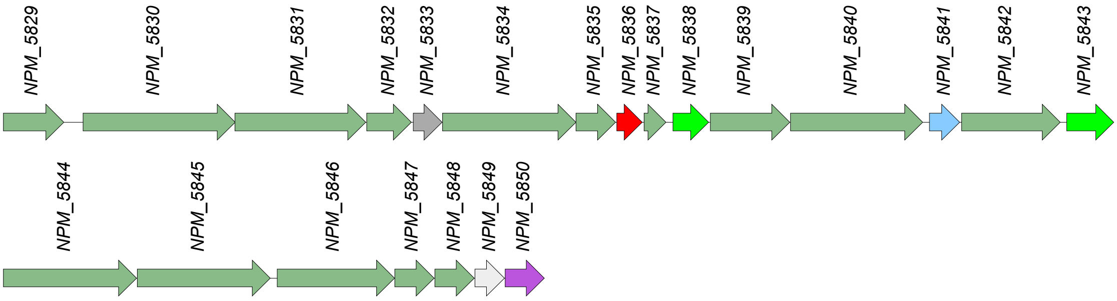

ORF	Protein size, aa	Proposed function	Closest homolog (origin)	% identity	GenBank accession number
NPM_5829	705	7-beta-(4-carbaxybutanamido) cephalosporanic acid acylase	*Fischerella muscicola* PCC 7414	86	WP_026086800
NPM_5830	1775	NRPS[a]	*Fischerella* sp. PCC 9339	72	WP_017307786
NPM_5831	1525	PKS[b]	*Nostoc* sp. '*Peltigera membranacea* cyanobiont'	92	AGH69811
NPM_5832	522	condensation domain protein	*Nostoc* sp. '*P. membranacea* cyanobiont'	99	AGH69812
NPM_5833	336	taurine catabolism dioxygenase TauD/TfdA	*Nostoc* sp. '*P. membranacea* cyanobiont'	99	AGH69813
NPM_5834	1557	NRPS[c]	*Nostoc* sp. '*P. membranacea* cyanobiont'	99	AGH69814
NPM_5835	462	condensation domain protein	*Nostoc* sp. '*P. membranacea* cyanobiont'	100	AGH69815
NPM_5836	294	JmjC domain protein	*Nostoc* sp. '*P. membranacea* cyanobiont'	100	AGH69816
NPM_5837	254	methyltransferase	*Nostoc* sp. '*P. membranacea* cyanobiont'	100	AGH69817
NPM_5838	418	diaminobutyrate-2-oxoglutarate transaminase	*Nostoc* sp. '*P. membranacea* cyanobiont'	99	AGH69818
NPM_5839	926	NRPS[d]	*Nostoc* sp. '*P. membranacea* cyanobiont'	98	AGH69819
NPM_5840	1542	PKS[b]	*Nostoc* sp. '*P. membranacea* cyanobiont'	99	AGH69820
NPM_5841	354	luciferase-like monooxygenase	*Nostoc* sp. '*P. membranacea* cyanobiont'	100	AGH69821
NPM_5842	1150	NRPS[e]	*Nostoc* sp. '*P. membranacea* cyanobiont'	99	AGH69822
NPM_5843	547	pyridoxal-dependent decarboxylase	*Nostoc* sp. '*P. membranacea* cyanobiont'	99	AGH69823
NPM_5844	1553	PKS[b]	*Scytonema hofmanni* UTEX 2349	74	WP_038296496
NPM_5845	1549	PKS[b]	*Nostoc* sp. '*P. membranacea* cyanobiont'	87	AGH69824
NPM_5846	1368	NRPS[f]	*Nostoc* sp. '*P. membranacea* cyanobiont'	99	AGH69825
NPM_5847	459	cytochrome P450	*Nostoc* sp. '*P. membranacea* cyanobiont'	100	AGH69826
NPM_5848	460	cytochrome P450	*Nostoc* sp. '*P. membranacea* cyanobiont'	99	AGH69827
NPM_5849	343	sucrase ferredoxin	*Nostoc* sp. '*P. membranacea* cyanobiont'	99	AGH69828
NPM_5850	457	MATE family efflux transporter	*Nostoc* sp. '*P. membranacea* cyanobiont'	99	AGH69829

[a] Deduced architecture of the encoded NRPS protein is AS–ACP–C–A–PCP.
[b] Deduced architecture of the encoded PKS protein is KS–AT–KR–ACP.
[c] Deduced architecture of the encoded NRPS protein is C–A–PCP–E.
[d] Deduced architecture of the encoded NRPS protein is C (partial)–A–PCP.
[e] Deduced architecture of the encoded NRPS protein is C–A–PCP.
[f] Deduced architecture of the encoded NRPS protein is C–A–PCP–TE.

Figure S18. *Continued.*

Nostoc sp. N6 *nrps6*

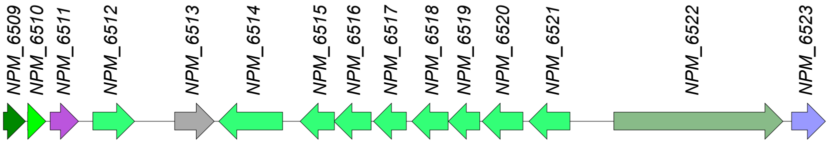

ORF	Protein size, aa	Proposed function	Closest homolog (origin)	% identity	GenBank accession number
NPM_6509	258	enoyl-ACP reductase	*Nostoc punctiforme* PCC 73102	99	ACC83816
NPM_6510	213	imidazoleglycerol-phosphate dehydratase	*N. punctiforme* PCC 73102	97	ACC83815
NPM_6511	329	multidrug ABC transporter ATP-binding protein	*N. punctiforme* PCC 73102	96	ACC83814
NPM_6512	487	polysaccharide export protein	*N. punctiforme* PCC 73102	93	ACC83813
NPM_6513	462	parallel beta-helix repeat-containing protein	*N. punctiforme* PCC 73102	65	ACC83812
NPM_6514	743	capsular exopolysaccharide biosynthesis protein	*N. punctiforme* PCC 73102	85	ACC83811
NPM_6515	399	glycosyltransferase WbuB	*N. punctiforme* PCC 73102	91	ACC83810
NPM_6516	433	GDP-mannose dehydrogenase	*N. punctiforme* PCC 73102	91	ACC83809
NPM_6517	384	glycosyltransferase	*N. punctiforme* PCC 73102	80	ACC83808
NPM_6518	421	O-antigen ligase	*Chlorogloeopsis* sp. PCC 7702	65	WP_017320729
NPM_6519	367	glycosyltransferase	*N. punctiforme* PCC 73102	83	ACC83806
NPM_6520	477	polysaccharide biosynthesis protein	*N. punctiforme* PCC 73102	85	ACC83805
NPM_6521	480	exopolysaccharide biosynthesis polyprenyl glycosylphosphotransferase	*N. punctiforme* PCC 73102	93	ACC83804
NPM_6522	1972	NRPS[a]	*Scytonema hofmanni* PCC 7110	65	WP_017748312
NPM_6523	393	aldo/keto reductase	*Nostoc punctiforme* PCC 73102	95	ACC83802

[a] Deduced architecture of the encoded NRPS protein is A–PCP–C–A–PCP–TE.

Figure S18. *Continued.*

Nostoc sp. '*Lobaria pulmonaria*' *nrps1*

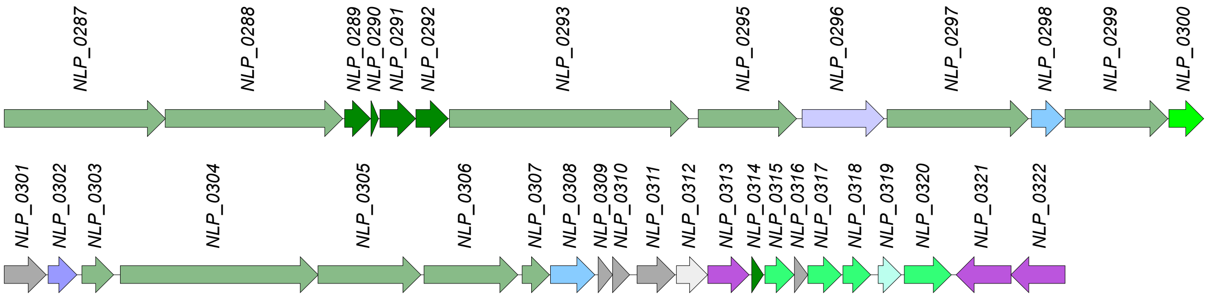

ORF	Protein size, aa	Proposed function	Closest homolog (origin)	% identity	GenBank accession number
NLP_0287	1776	NRPS[a]	*Calothrix* sp. PCC 7103	77	WP_019490343
NLP_0288	1953	PKS[b]	*Scytonema hofmanni* PCC 7110	62	WP_017744625
NLP_0289	284	3-hydroxyacyl-CoA dehydrogenase	*Nostoc* sp. PCC 7107	87	AFY44047
NLP_0290	82	D-alanyl carrier protein	*Fischerella muscicola* PCC 7414	88	WP_016862270
NLP_0291	394	acyl-CoA dehydrogenase	*S. hofmanni* UTEX 2349	94	WP_029634525
NLP_0292	356	FkbH-like protein	*Fischerella* sp. PCC 9605	88	WP_026736225
NLP_0293	2632	NRPS[c]	*Tolypothrix campylonemoides* VB511288	73	KIJ75066
NLP_0295	1082	NRPS[d]	*Mastigocladus laminosus* UU774	71	WP_044449763
NLP_0296	899	TonB-dependent receptor	*Gloeocapsa* sp. PCC 7428	74	AFZ30913
NLP_0297	1556	PKS[e]	*S. hofmanni* PCC 7110	71	WP_017744617
NLP_0298	356	luciferase-like monooxygenase	*S. hofmanni* PCC 7110	84	WP_017744616
NLP_0299	1134	NRPS[d]	*Fischerella* sp. PCC 9339	60	WP_017308724
NLP_0300	380	capreomycidine synthase	*Actinomadura oligospora* ATCC 43269	50	WP_034482709
NLP_0301	463	hypothetical protein	*Serratia marcescens* FGI94	26	AGB80661
NLP_0302	315	methyltransferase	*Calothrix* sp. PCC 7103	63	WP_019490341
NLP_0303	347	L-arginine β-hydroxylase	*Actinoalloteichus cyanogriseus* DSM 43889	46	WP_035276211
NLP_0304	2179	NRPS[f]	*Fischerella* sp. PCC 9339	66	WP_017307804
NLP_0305	1131	NRPS[d]	*Anabaena cylindrica* PCC 7122	71	AFZ60772
NLP_0306	1031	thioester reductase	*Limnoraphis robusta* CS-951	64	KKD39743
NLP_0307	301	methyltransferase	*A. cylindrica* PCC 7122	63	AFZ60771
NLP_0308	489	acetyl ornithine aminotransferase	*Acaryochloris marina MBIC11017*	68	ABW31361
NLP_0309	156	hypothetical protein	*A. cylindrica* PCC 7122	46	AFZ60769
NLP_0310	187	hypothetical protein	*A. cylindrica* PCC 7122	62	AFZ60768
NLP_0311	418	tyrosinase	*Gloeocapsa* sp. PCC 73106	63	ELR97043
NLP_0312	343	sucrase ferredoxin	*Mastigocoleus testarum* BC008	80	KST66001
NLP_0313	454	MATE family efflux transporter	*S. hofmanni* UTEX 2349	73	WP_029634515
NLP_0314	128	polysaccharide biosynthesis protein GtrA	*Microcystis panniformis* FACHB-1757	53	AKV67515
NLP_0315	323	glycosyltransferase	*Sulfuricella denitrificans* skB26	71	BAN36688
NLP_0316	147	WxcM-like protein	*Thioploca ingrica*	68	BAP57500
NLP_0317	369	AHBA synthase	*T. campylonemoides VB511288*	75	KIJ74864
NLP_0318	306	glucose-1-phosphate thymidylyltransferase	*Fischerella* sp. JSC-11	82	EHC10631
NLP_0319	253	methyltransferase	*Sulfurimonas gotlandica* GD1	42	EDZ62258
NLP_0320	517	glycosyltransferase	*Leptolyngbya* sp. NIES-2104	52	GAP94265
NLP_0321	604	ABC transporter	*Paenibacillus* sp. FJAT-27812	48	WP_053372800
NLP_0322	597	ABC transporter	*Nostoc* sp. PCC 7120	50	BAB74322

[a] Deduced architecture of the encoded NRPS protein is AS–ACP–C–A–PCP.
[b] Deduced architecture of the encoded PKS protein is KS–AT–MeT–KR–ACP.
[c] Deduced architecture of the encoded NRPS protein is C–A–PCP–C–A–PCP–C–E.
[d] Deduced architecture of the encoded NRPS protein is C–A–PCP.
[e] Deduced architecture of the encoded PKS protein is KS–AT–KR–ACP.
[f] Deduced architecture of the encoded NRPS protein is C–A–PCP–C–A–PCP.

Figure S18. *Continued.*

Nostoc sp. '*Lobaria pulmonaria*' *nrps2*

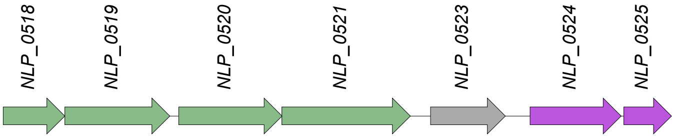

ORF	Protein size, aa	Proposed function	Closest homolog (origin)	% identity	GenBank accession number
NLP_0518	679	NRPS[a]	*Nostoc punctiforme* PCC 73102	65	ACC81528
NLP_0519	1156	PKS[b]	*N. punctiforme* PCC 73102	90	ACC84848
NLP_0520	1136	NRPS[c]	*N. punctiforme* PCC 73102	65	ACC84846
NLP_0521	1414	NRPS[d]	*Microcoleus vaginatus* FGP-2	60	EGK86184
NLP_0523	824	Ig family protein	*Aphanizomenon flos-aquae* NIES-81	58	WP_035081231
NLP_0524	1003	peptidase C39	*N. punctiforme* PCC 73102	95	ACC84843
NLP_0525	527	HlyD family secretion protein	*N. punctiforme* PCC 73102	88	ACC84842

[a] Deduced architecture of the encoded NRPS protein is A–PCP.
[b] Deduced architecture of the encoded PKS protein is KS–AT–ACP.
[c] Deduced architecture of the encoded NRPS protein is C–A–PCP.
[d] Deduced architecture of the encoded NRPS protein is C–A–PCP–TE.

Nostoc sp. '*Lobaria pulmonaria*' *hgl*

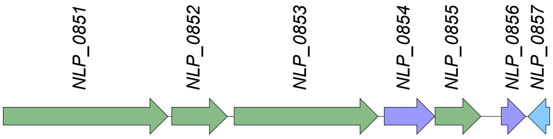

ORF	Protein size, aa	Proposed function	Closest homolog (origin)	% identity	GenBank accession number
NLP_0851	1816	heterocyst glycolipid synthase HglE[a]	*Nostoc punctiforme* PCC 73102	94	ACC78845
NLP_0852	572	heterocyst glycolipid ketoreductase HglG	*Nostoc* sp. '*Peltigera membranacea* cyanobiont'	96	AGJ76602
NLP_0853	1582	heterocyst glycolipid synthase HglC[b]	*Nostoc* sp. '*P. membranacea* cyanobiont'	94	AGJ76603
NLP_0854	558	heterocyst glycolipid enoyl reductase PfaD (2-nitropropane dioxygenase)	*Nostoc* sp. '*P. membranacea* cyanobiont'	98	AGJ76604
NLP_0855	504	heterocyst glycolipid synthase HglB[c]	*Nostoc* sp. '*P. membranacea* cyanobiont'	97	AGJ76605
NLP_0856	267	short-chain dehydrogenase/reductase	*Nostoc* sp. '*P. membranacea* cyanobiont'	95	AGJ76606
NLP_0857	239	4′-phosphopantetheinyl transferase HetI	*N. punctiforme* PCC 73102	87	ACC78839

[a] Deduced architecture of the encoded protein is KS–MAT–ACP–ACP–KR.
[b] Deduced architecture of the encoded protein is KS–CLF–AT.
[c] Deduced architecture of the encoded protein is ACP–TE.

Figure S18. *Continued.*

Nostoc sp. '*Lobaria pulmonaria*' *nrps3*

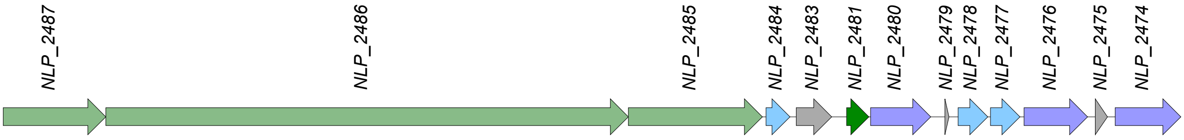

ORF	Protein size, aa	Proposed function	Closest homolog (origin)	% identity	GenBank accession number
NLP_2474	724	ABC transporter	*Nostoc punctiforme* PCC 73102	88	ACC80794
NLP_2475	134	hypothetical protein	*N. punctiforme* PCC 73102	96	ACC80793
NLP_2476	698	patatin	*N. punctiforme* PCC 73102	93	ACC80792
NLP_2477	325	MvdD family ATP-grasp ribosomal peptide maturase	*N. punctiforme* PCC 73102	95	ACC80791
NLP_2478	329	MvdD family ATP-grasp ribosomal peptide maturase	*N. punctiforme* PCC 73102	92	ACC80790
NLP_2479	44	microviridin precursor	*N. piscinale* CENA21	60	ALF56610
NLP_2480	663	ABC transporter	*N. punctiforme* PCC 73102	82	ACC80789
NLP_2481	245	short-chain dehydrogenase/ reductase	*Scytonema hofmanni* PCC 7110		WP_017742660
NLP_2483	389	McnG protein	*S. hofmanni* PCC 7110	81	WP_017742645
NLP_2484	264	methyltransferase	*S. hofmanni* PCC 7110	75	WP_017742659
NLP_2485	1477	NRPS[a]	*S. hofmanni* PCC 7110	77	WP_017742649
NLP_2486	5755	NRPS[b]	*S. hofmanni* PCC 7110	74	WP_017742656
NLP_2487	1132	NRPS[c]	*Microcystis* sp. NIVA-CYA 172/5	69	AAZ03550

[a] Deduced architecture of the encoded NRPS protein is C–A–PCP–TE.
[b] Deduced architecture of the encoded NRPS protein is C–A–PCP–C–A–PCP–C–A–PCP–C–A–PCP–C–A–MeT–PCP.
[c] Deduced architecture of the encoded NRPS protein is C–A–PCP.

Nostoc sp. '*Lobaria pulmonaria*' *nrps4*

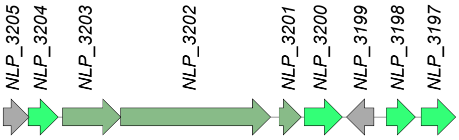

ORF	Protein size, aa	Proposed function	Closest homolog (origin)	% identity	GenBank accession number
NLP_3197	380	ADP-heptose:LPS heptosyltransferase	*Nostoc* sp. PCC 7107	68	AFY42227
NLP_3198	320	ADP-heptose:LPS heptosyltransferase	*Sulfurospirillum arsenophilum* NBRC 109478	50	WP_041959142
NLP_3199	302	hypothetical protein	*Pedobacter* sp. Leaf216	38	KQM63951
NLP_3200	418	glycosyltransferase	*Mastigocladopsis repens* PCC 10914	61	WP_017314697
NLP_3201	245	methyltransferase	*Synechocystis* sp. PCC 7509	53	WP_009632864
NLP_3202	1649	NRPS[a]	*Scytonema tolypothrichoides* VB-61278	45	WP_052486119
NLP_3203	642	NRPS[b]	*S. tolypothrichoides* VB-61278	69	WP_052486118
NLP_3204	328	aspartate racemase	*Sc. hofmanni* PCC 7110	58	WP_017748823
NLP_3205	286	hypothetical protein	*Tolypothrix bouteillei* VB521301	84	KIE11607

[a] Deduced architecture of the encoded NRPS protein is C–A–PCP–C.
[b] Deduced architecture of the encoded NRPS protein is A–PCP.

Figure S18. *Continued.*

Nostoc sp. '*Lobaria pulmonaria*' *nrps5*

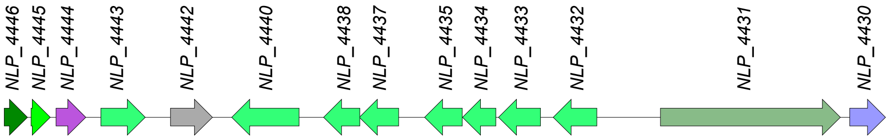

ORF	Protein size, aa	Proposed function	Closest homolog (origin)	% identity	GenBank accession number
NLP_4430	393	aldo/keto reductase	*Nostoc punctiforme* PCC 73102	91	ACC83802
NLP_4431	1983	NRPS[a]	*Scytonema hofmanni* PCC 7110	59	WP_017748312
NLP_4432	480	exopolysaccharide biosynthesis polyprenyl glycosylphosphotransferase	*N. punctiforme* PCC 73102	90	ACC83804
NLP_4433	462	polysaccharide biosynthesis protein	*N. punctiforme* PCC 73102	83	ACC83805
NLP_4434	367	glycosyltransferase	*N. punctiforme* PCC 73102	86	ACC83806
NLP_4435	414	O-antigen ligase	*Chlorogloeopsis* sp. PCC 7702	62	WP_017320729
NLP_4437	433	GDP-mannose dehydrogenase	*N. punctiforme* PCC 73102	90	ACC83809
NLP_4438	398	glycosyltransferase WbuB	*N. punctiforme* PCC 73102	93	ACC83810
NLP_4440	742	capsular exopolysaccharide biosynthesis protein	*N. punctiforme* PCC 73102	84	ACC83811
NLP_4442	462	parallel beta-helix repeat-containing protein	*N. punctiforme* PCC 73102	62	ACC83812
NLP_4443	489	polysaccharide export protein	*N. punctiforme* PCC 73102	92	ACC83813
NLP_4444	327	multidrug ABC transporter ATP-binding protein	*N. punctiforme* PCC 73102	94	ACC83814
NLP_4445	211	imidazoleglycerol-phosphate dehydratase	*N. punctiforme* PCC 73102	96	ACC83815
NLP_4446	258	enoyl-ACP reductase	*N. punctiforme* PCC 73102	96	ACC83816

[a] Deduced architecture of the encoded NRPS protein is A–PCP–C–A–PCP–TE.

Nostoc sp. '*Lobaria pulmonaria*' *nrps6*

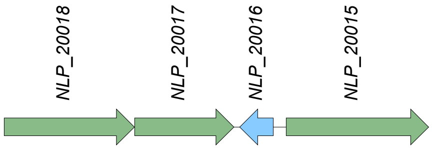

ORF	Protein size, aa	Proposed function	Closest homolog (origin)	% identity	GenBank accession number
NLP_20015	1569	NRPS[a]	*Scytonema hofmanni* PCC 7110	90	WP_051077115
NLP_20016	369	α/β hydrolase	*S. hofmanni* UTEX 2349	93	WP_029631593
NLP_20017	1091	NRPS[b]	*S. hofmanni* PCC 7110	91	WP_017747558
NLP_20018	1436	NRPS[c]	*S. hofmanni* PCC 7110	86	WP_017747559

[a] Deduced architecture of the encoded NRPS protein is C–A–MeT–PCP.
[b] Deduced architecture of the encoded NRPS protein is C–A–PCP.
[c] Deduced architecture of the encoded NRPS protein is C–A–PCP–TE.

Figure S18. *Continued.*

Nostoc sp. '*Lobaria pulmonaria*' *nrps7*

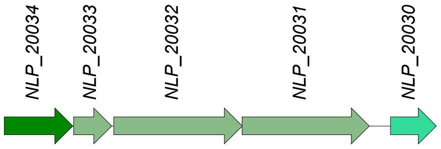

ORF	Protein size, aa	Proposed function	Closest homolog (origin)	% identity	GenBank accession number
NLP_20030	503	EmrB/QacA family drug resistance transporter	*Mastigocladopsis repens* PCC 10914	64	WP_017317776
NLP_20031	1399	NRPS[a]	*Scytonema hofmanni* PCC 7110	67	WP_017745372
NLP_20032	1414	PKS[b]	*Fischerella* sp. PCC 9339	48	WP_017308083
NLP_20033	418	cytochrome P450	*Scytonema hofmanni* PCC 7110	58	WP_017745373
NLP_20034	756	AMP-dependent synthetase/ligase	*Scytonema hofmanni* PCC 7110	60	WP_017745374

[a] Deduced architecture of the encoded NRPS protein is C–A–PCP–TE.
[b] Deduced architecture of the encoded PKS protein is KS–AT–MeT–ACP.

Figure S18. *Continued.*

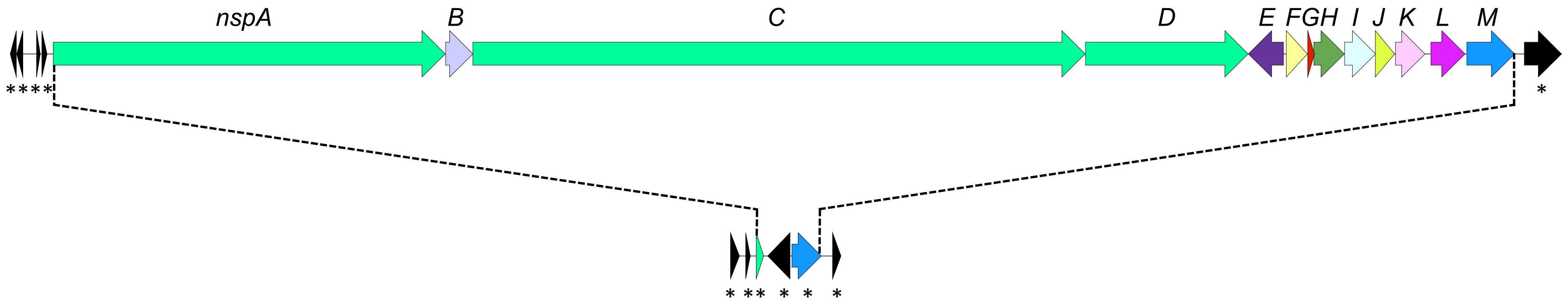

Figure S19. Nosperin gene cluster (*nsp*) in *Nostoc* sp. N6 (top) and its remnants in the *L. pulmonaria* cyanobiont (bottom). The genes are color coded as in (Kampa et al., 2013). Pseudogenes are denoted with an asterisk (*), black arrows represent transposase genes.

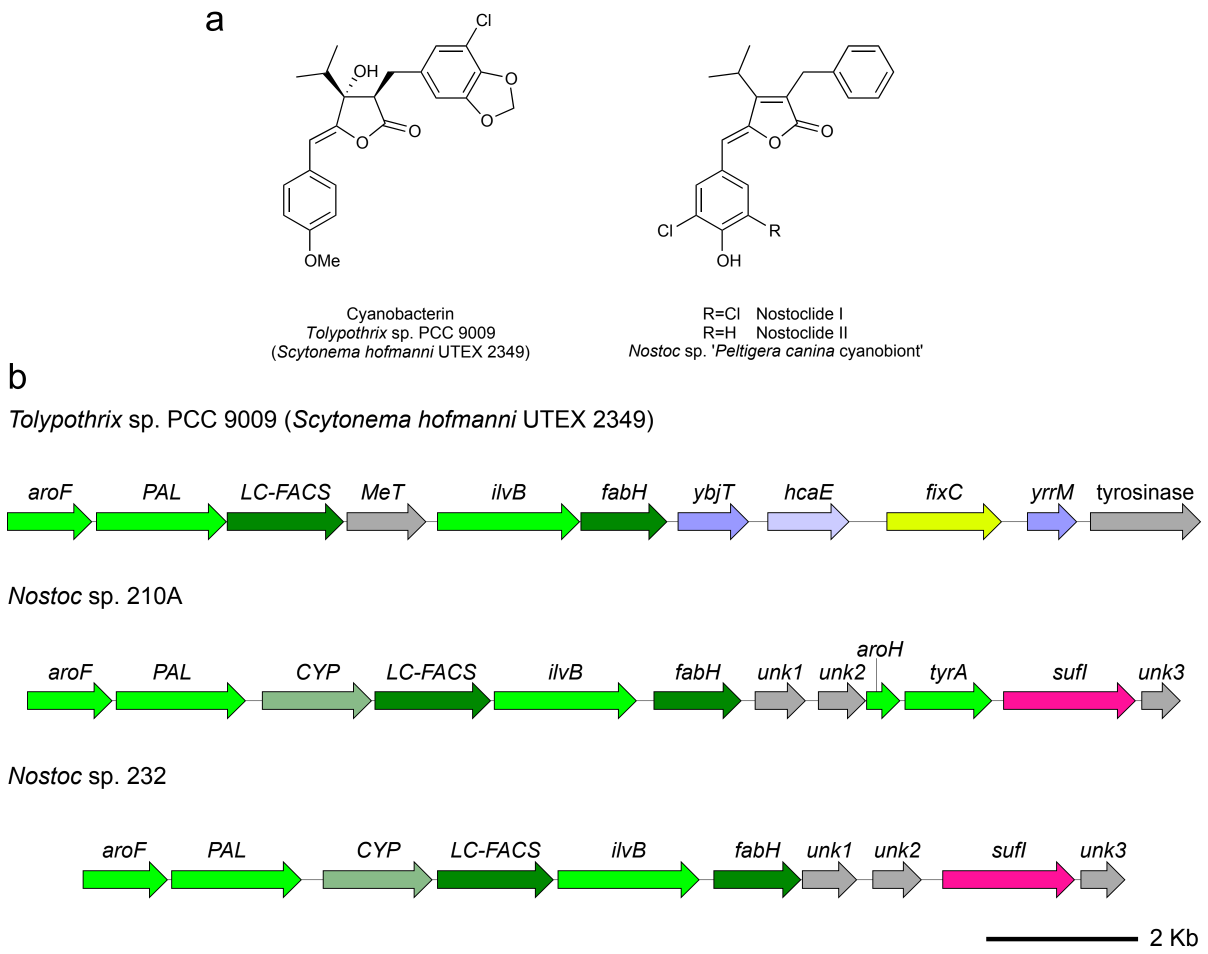

Figure S20. Structures (**a**) and putative biosynthetic gene clusters of cyanobacterin and nostoclides (**b**). *aroF*, 3-deoxy-7-phosphoheptulonate synthase; *PAL*, aromatic amino acid lyase; *CYP*, cytochrome P450; *LC-FACS*, long-chain-fatty-acid–CoA ligase; *MeT*, putative *O*-methyltransferase; *ilvB*, acetolactate synthase; *fabH*, 3-oxoacyl-ACP synthase; *ybjT*, NAD(P)-dependent oxidoreductase; *hcaE*, phenylpropionate dioxygenase or related ring-hydroxylating dioxygenase; *fixC*, putative halogenase; *yrrM*, predicted *O*-methyltransferase; *aroH*, chorismate mutase; *tyrA*, bifunctional chorismate mutase/prephenate dehydrogenase; *sufI*, multicopper oxidase; *unk*, unknown.

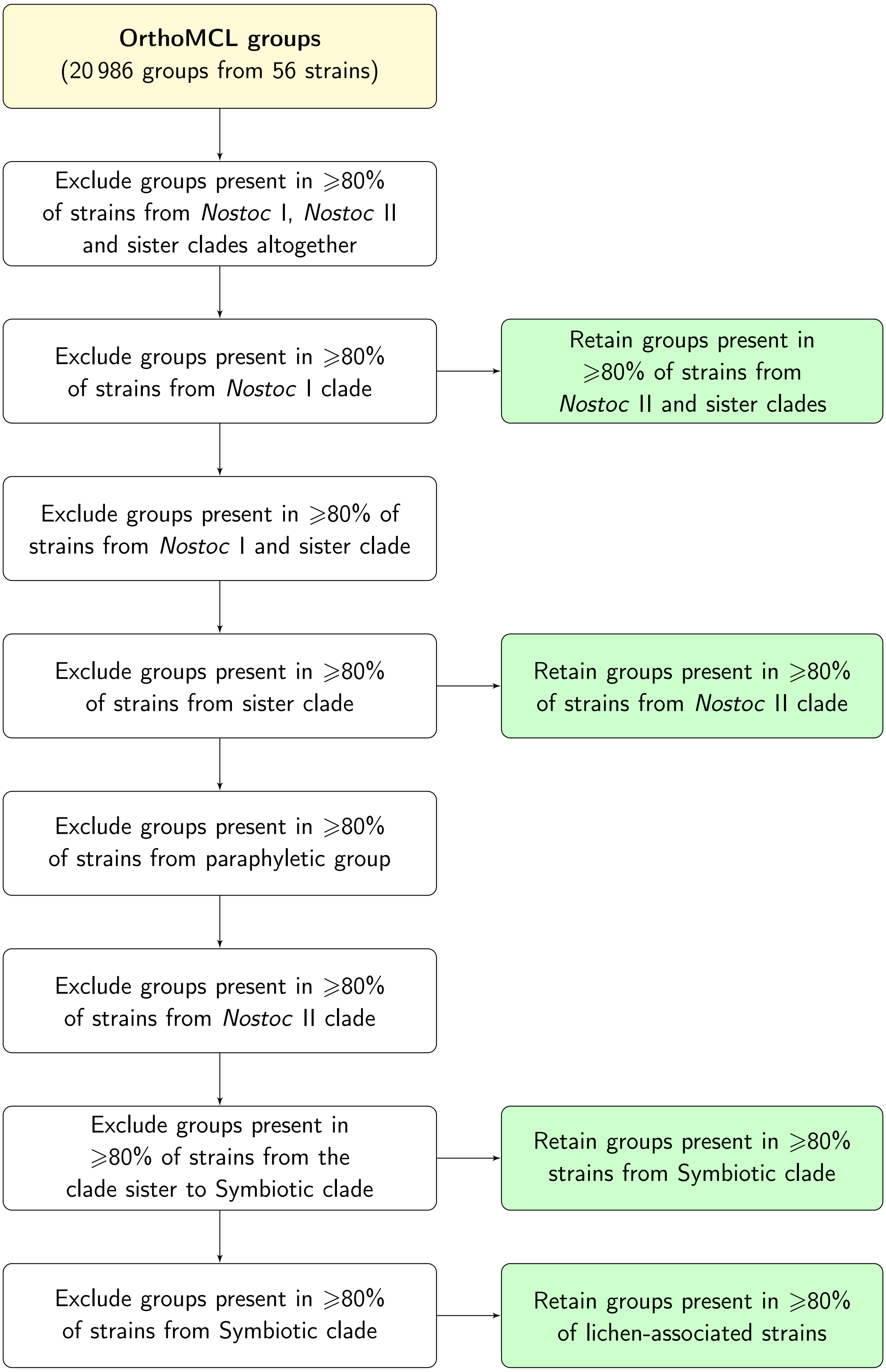

Figure S21. Workflow for extracting orthologous groups specific to different clades. The clades and groups are shown in Figure 4 where "paraphyletic group" corresponds to strains highlighted with blue, "symbiotic" – to strains highlighted with green and lichen-associated strains are shown in bold.

Table S1. Assemblies of *Nostoc* genomes

Strain	BioProject	BioSample	Accession	Sequencing library	Number of scaffolds	Assembly size	Mean coverage
Nostoc sp. N6	PRJNA279350	SAMN03445855	–	Illumina NexteraXT and Illumina Nextera Mate pair	–	8.9 Mb	413×
Nostoc sp. N6	PRJNA275880	SAMN03354403	–	Illumina NexteraXT and Illumina Nextera Mate pair	–	7.34 Mb	37×
Nostoc sp. 210A	PRJNA389199	SAMN07191894	NOLJ00000000	Illumina NexteraXT and Illumina Nextera Mate pair	30	8.33 Mb	90×
Nostoc sp. 213	PRJNA389200	SAMN07191895	NOLI00000000	Illumina NexteraXT	378	8.33 Mb	25×
Nostoc sp. 232	PRJNA389202	SAMN07191897	NOLK00000000	Illumina NexteraXT	397	9.16 Mb	35×
Nostoc sp. '*P. malacea*'	PRJNA389205	SAMN07192327	NSHF00000000	Roche 454 and Illumina Mate pair v2	985	8.52 Mb	13×

Table S2. Completeness and contamination of *Nostoc* genome assemblies assessed with CheckM (Parks et al., 2015)

**Strain**	**Marker lineage**[a]	**Number of genomes**[b]	**Number of markers**[c]	**Number of marker sets**[d]	**Completeness**	**Contamination**	**Strain heterogeneity**
Nostoc sp. N6	Cyanobacteria	82	579	450	99.44	1.19	0.00
Nostoc sp. '*L. pulmonaria*'	Cyanobacteria	82	579	450	99.55	0.44	0.00
Nostoc sp. 210A	Cyanobacteria	82	579	450	99.22	1.33	0.00
Nostoc sp. 213	Cyanobacteria	82	579	450	99.00	0.74	0.00
Nostoc sp. 232	Cyanobacteria	82	579	450	98.78	1.37	11.11
Nostoc sp. '*P. malacea*'	Cyanobacteria	82	579	450	94.54	4.60	44.44

[a] Lineage used for inferring marker set
[b] Number of reference genomes used to infer marker set
[c] Number of inferred marker genes
[d] Number of inferred co-located marker sets

Table S3. COG category assignment of the proteins encoded in the genome of *Nostoc* sp. N6.

COG functional category		chr	pNPM1	pNPM2	pNPM3	pNPM4	pNPM5	pNPM6	pNPM7	pNPM8	pNPM9	pNPM10	Total
**J**	Translation, ribosomal structure and biogenesis	246	1	2	0	2	0	0	0	0	0	0	251
**A**	RNA processing and modification	1	0	0	0	0	0	0	0	0	0	0	1
**K**	Transcription	154	8	3	0	0	0	1	0	2	0	0	168
**L**	Replication, recombination and repair	174	7	11	4	2	1	3	2	5	1	1	211
**B**	Chromatin structure and dynamics	2	0	0	0	0	0	0	0	0	0	0	2
**D**	Cell cycle control, cell division, chromosome partitioning	57	2	1	2	1	1	0	2	2	2	0	70
**Y**	Nuclear structure	0	0	0	0	0	0	0	0	0	0	0	0
**V**	Defense mechanisms	166	8	1	0	2	0	0	0	2	0	0	179
**T**	Signal transduction mechanisms	336	6	3	2	2	2	2	0	0	0	0	353
**M**	Cell wall/membrane/envelope biogenesis	306	3	2	0	0	2	0	0	0	0	0	313
**N**	Cell motility	53	0	0	0	0	0	0	0	0	0	0	53
**Z**	Cytoskeleton	0	0	0	0	0	0	0	0	0	0	0	0
**W**	Extracellular structures	0	0	0	0	0	0	0	0	0	0	0	0
**U**	Intracellular trafficking, secretion, and vesicular transport	51	3	1	0	0	0	0	0	0	0	0	55
**O**	Posttranslational modification, protein turnover, chaperones	212	2	1	0	0	0	0	0	1	0	0	216
**C**	Energy production and conversion	208	0	1	0	0	0	0	0	0	0	0	209
**G**	Carbohydrate transport and metabolism	192	0	0	0	0	0	0	0	2	0	0	194
**E**	Amino acid transport and metabolism	240	1	0	0	0	0	0	0	0	0	1	242
**F**	Nucleotide transport and metabolism	72	0	1	0	0	0	0	0	1	0	0	74
**H**	Coenzyme transport and metabolism	257	2	2	0	0	0	0	0	0	0	0	261
**I**	Lipid transport and metabolism	127	1	2	0	0	0	0	0	0	0	0	130
**P**	Inorganic ion transport and metabolism	233	4	1	0	0	0	0	0	0	0	0	238
**Q**	Secondary metabolites biosynthesis, transport and catabolism	132	1	0	0	0	0	0	0	1	0	0	134
**X**	Mobilome: prophages, transposons	84	7	1	0	0	0	1	0	2	2	0	97
**R**	General function prediction only	517	9	6	0	1	2	0	3	3	0	0	541
**S**	Function unknown	294	5	1	2	2	2	1	3	1	0	2	313
	Assigned to COGs	4115	70	40	10	12	10	8	10	22	5	4	4306
	Not assigned to COGs	2090	110	82	20	26	22	19	20	47	9	16	2461

Table S4. COG category assignment of the proteins encoded in the genome of *Nostoc* sp. '*Lobaria pulmonaria* cyanobiont'.

**COG functional category**		**chr**	**pNLP1**	**pNLP2**	**pNLP3**	**pNLP4**	**Total**
**J**	Translation, ribosomal structure and biogenesis	234	1	0	1	0	235
**A**	RNA processing and modification	1	0	0	0	0	1
**K**	Transcription	118	3	2	1	1	126
**L**	Replication, recombination and repair	127	4	2	4	2	139
**B**	Chromatin structure and dynamics	2	0	0	0	0	2
**D**	Cell cycle control, cell division, chromosome partitioning	52	2	1	1	1	57
**Y**	Nuclear structure	0	0	0	0	0	0
**V**	Defense mechanisms	134	0	0	3	3	140
**T**	Signal transduction mechanisms	314	3	0	0	0	317
**M**	Cell wall/membrane/envelope biogenesis	275	1	0	0	0	276
**N**	Cell motility	45	0	0	0	0	45
**Z**	Cytoskeleton	0	0	0	0	0	0
**W**	Extracellular structures	0	0	0	0	0	0
**U**	Intracellular trafficking, secretion, and vesicular transport	40	3	0	1	0	44
**O**	Posttranslational modification, protein turnover, chaperones	188	0	0	1	0	189
**C**	Energy production and conversion	208	0	0	0	0	208
**G**	Carbohydrate transport and metabolism	189	0	1	0	0	190
**E**	Amino acid transport and metabolism	231	1	0	1	1	234
**F**	Nucleotide transport and metabolism	69	0	0	1	0	70
**H**	Coenzyme transport and metabolism	245	2	1	0	0	248
**I**	Lipid transport and metabolism	114	0	1	0	0	115
**P**	Inorganic ion transport and metabolism	206	0	0	0	1	207
**Q**	Secondary metabolites biosynthesis, transport and catabolism	103	1	6	0	0	110
**X**	Mobilome: prophages, transposons	33	3	2	0	0	38
**R**	General function prediction only	464	1	1	4	1	471
**S**	Function unknown	288	2	0	1	0	291
	Assigned to COGs	3681	27	16	19	10	3753
	Not assigned to COGs	1630	55	20	41	23	1769

Table S5. COG category assignment of the proteins encoded in the genomes of *Nostoc* and *Anabaena* strains

COG functional category		*Nostoc* sp. N6	*Nostoc* sp. 'L.pulmonaria'	*Nostoc punctiforme*	*Nostoc* sp. PCC 7107	*Nostoc* sp. PCC 7120	*Nostoc* sp. PCC 7524	*Anabaena variabilis*	*Anabaena* sp. PCC 7108	*Anabaena* sp. 90	*Anabaena cylindrica*
J	Translation, ribosomal structure and biogenesis	251	235	235	221	235	224	228	210	208	228
A	RNA processing and modification	1	1	1	0	1	1	1	0	0	0
K	Transcription	168	126	150	114	154	132	135	120	77	135
L	Replication, recombination and repair	211	139	163	125	153	138	152	158	140	180
B	Chromatin structure and dynamics	2	2	2	2	2	2	2	2	1	3
D	Cell cycle control, cell division, chromosome partitioning	70	57	75	56	64	53	59	60	45	62
Y	Nuclear structure	0	0	0	0	0	0	0	0	0	0
V	Defense mechanisms	179	140	170	140	187	157	135	152	151	201
T	Signal transduction mechanisms	353	317	415	325	362	301	342	258	164	320
M	Cell wall/membrane/envelope biogenesis	313	276	325	276	312	273	292	263	236	262
N	Cell motility	53	45	52	48	35	45	37	43	33	35
Z	Cytoskeleton	0	0	0	0	0	0	0	0	0	0
W	Extracellular structures	0	0	0	1	1	1	1	0	0	0
U	Intracellular trafficking, secretion, and vesicular transport	55	44	48	52	64	38	50	32	28	66
O	Posttranslational modification, protein turnover, chaperones	216	189	228	190	206	200	216	181	175	196
C	Energy production and conversion	209	208	218	181	184	195	209	182	144	176
G	Carbohydrate transport and metabolism	194	190	208	151	168	152	165	144	111	160
E	Amino acid transport and metabolism	242	234	249	201	224	206	238	187	180	190
F	Nucleotide transport and metabolism	74	70	76	73	73	76	77	67	67	71
H	Coenzyme transport and metabolism	261	248	256	208	233	230	239	206	190	224
I	Lipid transport and metabolism	130	115	150	81	93	83	105	91	68	83
P	Inorganic ion transport and metabolism	238	207	239	220	283	256	278	179	141	209
Q	Secondary metabolites biosynthesis, transport and catabolism	134	110	170	77	98	85	110	74	72	92
X	Mobilome: prophages, transposons	97	38	71	57	140	85	99	74	70	82
R	General function prediction only	541	471	546	455	457	457	444	438	405	485
S	Function unknown	313	291	335	272	305	255	280	226	194	300
	Assigned to COGs	4306	3753	4382	3526	4034	3645	3894	3347	2900	3760
	Not assigned to COGs	2461	1769	2307	1711	2095	1804	1816	1822	1611	2078
	Total	6767	5522	6689	5237	6129	5449	5710	5169	4511	5838

Table S6. Scytonemin biosynthesis genes of *Nostoc punctiforme* and their presence in lichen-associated *Nostoc* strains. Pseudogenes are denoted with an asterisk (*).

**Locus tag**	**Gene symbol**	**Encoded protein**	***Nostoc* sp. *N6***	***Nostoc* sp. '*L. pulmonaria*'**	***Nostoc* sp. 210A**	***Nostoc* sp. 213**	***Nostoc* sp. 232**	***Nostoc* sp. '*P. malacea*'**
Npun_R1259	–	protein of unknown function	NPM_3284	NLP_2898	+	+	+	+
Npun_R1260	*aroG*	3-deoxy-7-phosphoheptulonate synthase	NPM_3285	NLP_1938	+	+	+	+
Npun_R1261	*trpD*	anthranilate phosphoribosyltransferase	NPM_3286	NLP_1937	+	+	+	+
Npun_R1262	*trpB*	tryptophan synthase subunit beta	NPM_3287	NLP_1936	+	+	+	+
Npun_R1263	*tyrP*	tyrosinase	–	NLP_1935	+	–	–	+
Npun_R1264	*trpA*	tryptophan synthase subunit alpha	NPM_3288	NLP_1934	+	+	+	+
Npun_R1265	*trpC*	indole-3-glycerol phosphate synthase	NPM_3289	NLP_1932	+	+	+	+
Npun_R1266	*trpE*	anthranilate synthase component I	NPM_3290	NLP_1931	+	+	+	+
Npun_R1267	*aroB*	3-dehydroquinate synthase	NPM_3291	NLP_1930	+	+	+	+
Npun_R1268	*frnE*	DSBA oxidoreductase/FrnE protein	NPM_3292*	NLP_1929	–	+	+	+
Npun_R1269	*tyrA*	bifunctional chorismate mutase/prephenate dehydrogenase	NPM_3293	NLP_1928	+	+	+*	+
Npun_R1270	–	group 1 glycosyltransferase	NPM_3294	NLP_1927	+	+	+	+
Npun_R1271	*scyF*	NHL repeat-containing protein	NPM_3295	NLP_1926	+	+	+	+
Npun_R1272	*scyE*	NHL repeat-containing protein	NPM_3296	NLP_1925	+	+	+	+
Npun_R1273	*scyD*	NHL repeat-containing protein	NPM_3297	NLP_1924	+	+	+	+
Npun_R1274	*scyC*	protein of unknown function	NPM_3298	NLP_1923	+	+	+	+
Npun_R1275	*scyB*	glutamate/leucine dehydrogenase	NPM_3299	NLP_1922	+	+	+	+
Npun_R1276	*scyA*	acetolactate synthase large subunit	NPM_3300	NLP_1921	+	+	+	+
Npun_F1277	–	PAS/PAC sensor signal transduction histidine kinase	NPM_3301	NLP_1920	+	+	+	+
Npun_F1278	*araC*	AraC family two component transcriptional regulator	NPM_3302	NLP_1919	+	+	+	+
Npun_F5232	–	protein of unknown function	NPM_5171	NLP_5009	+	+	+	+
Npun_F5233	–	putative metal-dependent hydrolase	NPM_5170	NLP_5010	+	+	+	+
Npun_F5234	*ubiA*	UbiA family prenyltransferase	NPM_5169	NLP_5011	+	+	+	+
Npun_F5235	–	protein of unknown function	NPM_5168	NLP_5012	+	+	+	+
Npun_F5236	–	type I phospodiesterase	NPM_5167	NLP_5013	+	+	+	+

Table S7. Number of transposase genes in the *Nostoc* sp. N6 and *Nostoc* sp. '*Lobaria pulmonaria* cyanobiont' genomes.

Replicon	Intact genes	Pseudogenes
***Nostoc* sp. N6**		
chromosome	88	85
pNPM1	3	2
pNPM2	2	2
pNPM3	0	1
pNPM4	0	0
pNPM5	1	0
pNPM6	0	0
pNPM7	0	0
pNPM8	1	0
pNPM9	2	0
pNPM10	0	0
Total	97	90
***Nostoc* sp. '*Lobaria pulmonaria*'**		
chromosome	29	114
pNLP1	5	0
pNLP2	1	4
pNLP3	0	0
pNLP4	0	0
Total	35	118

Table S8. IS family composition of the *Nostoc* sp. N6 and *Nostoc* sp. '*Lobaria pulmonaria* cyanobiont' genomes.

**IS family**	**Copy number**	
	***Nostoc* sp. N6**	***Nostoc* sp. '*L. pulmonaria*'**
IS110	0	1
IS1380	1	1
IS1595	1	0
IS1634	22	1
IS200/IS605	15	8
IS4	6	6
IS5	12	1
IS630	25	3
IS701	1	5
ISAzo13	5	1
ISNCY	0	4
Tn3	1	0
Undefined	8	4
Total	97	35

Table S9. Intein-containing proteins encoded in the genome of *Nostoc* sp. N6

Locus tag	Mature protein	Functional homolog without intein (% protein sequence identity/similarity)
NPM_0997	replicative DNA helicase DnaB	NPM_1772 (55%/72%), NPM_1944 (56%/73%), NPM_4704 (56%/73%), NPM_10102 (59%/78%)
NPM_1656	phosphoenolpyruvate synthase	NPM_3568 (25%/46%), NPM_5130 (33%/46%)
NPM_2566	ribonucleoside-diphosphate reductase	–
NPM_2586	tRNA-splicing ligase RtcB	NPM_1543 (30%/43%)
NPM_5024	superfamily II DNA/RNA helicase	–

Table S10. Distibution of thymidilate synthase (*thyA*/*thyX*) genes among Cyanobacteria. Pseudogenes are denoted with an asterisk (*).

Strain	*thyA*	*thyX*
Nostoc sp. N6	−	+
Nostoc sp. 210A	+	+*
Nostoc sp. 213	−	+
Nostoc sp. 232	−	+
Nostoc sp. '*Lobaria pulmonaria*'	+	−
Nostoc sp. '*Peltigera malacea*'	−	+
Nostoc punctiforme	−	+
Nostoc sp. KVJ20	−	+
Nostoc sp. PCC 7107	−	+
Nostoc sp. PCC 7120	−	+
Nostoc sp. PCC 7524	+	−
Nostoc sp. NIES-3756	+	−
Anabaena variabilis ATCC 29413	−	+
Anabaena sp. PCC 7108	−	+
Anabaena sp. WA102	−	+
Anabaena sp. 90	+	−
Anabaena cylindrica PCC 7122	+	−
Trichormus azollae 0708	+	−
Calothrix sp. PCC 7507	−	+
Cyanothece spp. (6 strains)	+	−
Cylindrospermum stagnale PCC 7417	+	−
Gloeocapsa sp. PCC 7428	+	−
Microcoleus sp. PCC 7113	−	+
Microcystis spp. (4 strains)	+	−
Nodularia spumigena CCY9414	−	+
Pleurocapsa sp. PCC 7327	+	−
Rivularia sp. PCC 7116	−	+
Synechococcus spp. (18 strains)	−	+

Table S11. Intervening sequences in ribonucleoside-diphosphate reductase *nrdJ* genes from *Nostoc* and *Anabaena* strains. Genes originally annotated as "pseudo" are denoted with an asterisk (*).

**Strain**	**Locus tag**	**Intervening sequence**
Nostoc sp. N6	NPM_2566	intein
Nostoc sp. 210A	–	group I intron
Nostoc sp. 213	–	intein
Nostoc sp. 232	–	intein
Nostoc sp. '*Lobaria pulmonaria*'	NLP_3546	group I intron
Nostoc sp. '*Peltigera malacea*'	–	group I intron
Nostoc punctiforme	Npun_F2495*	group I intron
Nostoc sp. PCC 7107	Nos7107_2758	intein
Nostoc sp. PCC 7120	all4035	intein
Nostoc sp. PCC 7524	Nos7524_1315	2 inteins
Anabaena variabilis ATCC 29413	Ava_1670	–
Anabaena sp. PCC 7108	Ana7108_0097	group I intron
Anabaena sp. 90	ANA_C12699,<br>ANA_C12700<br>ANA_C12701*	group II intron with<br>reverse-transcriptase/maturase,<br>intein
Anabaena cylindrica PCC 7122	Anacy_1019*	group I intron, intein
Trichormus azollae 0708	Aazo_1083	2 inteins

Table S12. Expression of phospholipases C and D by the *Peltigera membranacea* mycobiont

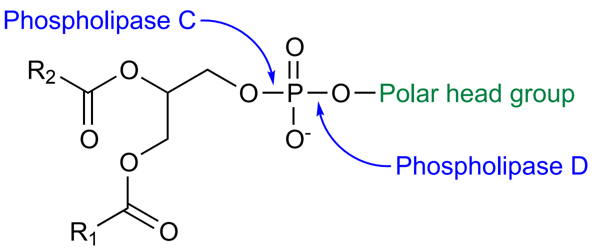

Gene symbol	Encoded protein	RPKM values			
		CDS	Gene	Transcript with introns	Transcript without introns
plc1	phospholipase C1	78	64	63	74
plc2	phospholipase C2	65	65	59	59
pld	phospholipase D	122	103	95	110
tub-2	β-tubulin	897	656	527	658
gpd-1	glycerol-3-phosphate dehydrogenase	1396	1019	789	983

Table S13. Gene clusters encoding glycolipid synthases, polyketide synthases (PKSs) and non-ribosomal peptide synthases (NRPSs) in the *Nostoc* sp. N6 and *Nostoc* sp. '*Lobaria pulmonaria* cyanobiont' genomes.

Gene cluster	Type	Size, kb	Gene cluster location	Actual or predicted product	Reference
***Nostoc* sp. N6**					
nos	NRPS-PKS	41.6	NPM_0706-NPM_0718	Nostopeptolide-like compound	Hoffmann et al., 2003; Liaimer et al., 2015
ncp	NRPS	25.2	NPM_1843-NPM_1844	Nostocyclopeptide	Becker et al., 2004
nsp	PKS-NRPS	59.5	NPM_1889-NPM_1901	Nosperin	Kampa et al., 2013
nrps1	NRPS	5.5	NPM_1940	Unknown	
nrps2	NRPS	13.6	NPM_2203-NPM_2206	Unknown	
nrps3	NRPS	46.1	NPM_3792-NPM_3822	Banyaside/suomilide-like compound	Liaimer et al., 2016
nrps4	NRPS	8.0	NPM_4088	Unknown	
pks1	PKS	27.2	NPM_4996-NPM_5009	Unknown	
nrps5	NRPS	31.9	NPM_5146-NPM_5156	Unknown	
hgl	glycolipid synthase	17.9	NPM_5725-NPM_5731	Heterocyst glycolipid	Campbell et al., 1997
pks2	PKS-NRPS		NPM_5829-NPM_5850	Unknown	
nrps6	NRPS	28.8	NPM_6509-NPM_6523	Unknown	
***Nostoc* sp. '*Lobaria pulmonaria*'**					
nrps1	NRPS-PKS	74.8	NLP_0287-NLP_0322	Unknown	
nrps2	NRPS-PKS	22.1	NLP_0518-NLP_0525	Unknown	
hgl	glycolipid synthase	18.1	NLP_0851-NLP_0857	Heterocyst glycolipid	Campbell et al., 1997
nrps3	NRPS	38.9	NLP_2474-NLP_2487 (including microviridin gene cluster)	Cyanopeptolin	Rounge et al., 2008
nrps4	NRPS	14.9	NLP_3197-NLP_3205	Unknown	
nrps5	NRPS	29.1	NLP_4430-NLP_4446	Unknown	
nrps6	NRPS	14.0	NLP_20015-NLP_20018	Unknown	
nrps7	NRPS-PKS	14.3	NLP_20030-NLP_20034	Unknown	

Table S14. Analysis of adenylation domains present in the non-ribosomal peptide synthases encoded in the the *Nostoc* sp. N6 and *Nostoc* sp. '*Lobaria pulmonaria* cyanobiont' genomes. Substrate specificity was determined using NRPSpredictor2 (*http://nrps.informatik.uni-tuebingen.de*; Röttig et al., 2011).

Protein	Adenylation domain	Nonribosomal code	Predicted specificity (similarity to the nearest neighbor)
**NPM_0709**	1	DVQFIAHVVK	Pro (100%)
	2	DAFFLGVTFK	Ile (100%)
**NPM_0710**	1	DVWHISLIDK	Ser (100%)
	2	DVQFIAHLAK	Pro (100%)
	3	DAWTIAAVCK	Phe (90%)
**NPM_0712**	1	DVQFIAHVAK	Pro (90%)
(*pseudo*)	2	DAWQFGLIDK	Gln (100%)
**NPM_1843**	1	DASTVAAVCK	Tyr (100%)
(NcpA)	2	DILQLGLIWK	Gly (100%)
	3	DAWQFGLIDK	Gln (100%)
**NPM_1844**	1	DVQFIAHVVK	Pro (100%)
(NcpB)	2	DVWHISLIDK	Ser (100%)
	3	DAFFLGVTFK	Ile (100%)
	4	DAWTIGAVCK	Phe (100%)
**NPM_1899**	1	DILQLGLIWK	Gly (100%)
(NspC)	2	DVQYIAQVIK	Pro (80%)
**NPM_1940**	1	DASTVAAVCK	Tyr (100%)
	2 (truncated)	Not assigned	Not assigned
**NPM_2203**	1	DAFTIAAVWK	Phe (80%)
**NPM_2204**	1	DAFFLGVTFK	Ile (100%)
**NPM_2206**	1	DAWFLGNVVK	Leu (100%)
**NPM_3792**	1	DAFFLGVTFK	Ile (100%)
**NPM_3801**	1	DVHFICLLVK	Pro (60%)
**NPM_4088**	1	DAHITTHVVK	Tyr (60%)
	2	DVQFIAHVVK	Pro (100%)
**NPM_5151**	1	DAFFLGVTFK	Ile (100%)
	2	DTEDIGSVVK	Lys (70%)
**NPM_5152**	1	DGLLIGAVFK	Val (80%)
**NPM_5153**	1	DLGAIGCVIK	Asn (60%)
	2	DLFNNALTYK	Ala (100%)
**NPM_5155**	1	DLGAIGCVIK	Asn (60%)
**NPM_5830**	1	DLFNNALTYK	Ala (100%)
**NPM_5834**	1	DLTKIGHVGK	Asn (90%)

Continued on next page

Table S14. *(continued from previous page)*

Protein	Adenylation domain	Nonribosomal code	Predicted specificity (similarity to the nearest neighbor)
**NPM_5839**	1	DIWEMVADDK	Ser (50%)
**NPM_5842**	1	Not assigned	Not assigned
**NPM_5846**	1	Not assigned	Not assigned
**NPM_6522**	1	Not assigned	Not assigned
	2	DVWHFSLIDK	Ser (100%)
**NLP_0287**	1	DLFNNALTYK	Ala (100%)
**NLP_0293**	1	DLFNNALTYK	Ala (100%)
	2	DVWHISLVDK	Ser (100%)
**NLP_0295**	1	DILQLGMIWK	Gly (100%)
**NLP_0299**	1	DVEEIGVVTK	Orn (100%)
**NLP_0304**	1	DVWHISLVDK	Ser (100%)
	2	DFWNIGMVHK	Thr (100%)
**NLP_0305**	1	DAPTLGAVNK	Bht (70%)
**NLP_0518**	1	DFWNIGMVHK	Thr (100%)
**NLP_0520**	1	DILQLGMIWK	Gly (100%)
**NLP_0521**	1	DAFWLGGTFK	Val (90%)
**NLP_2485**	1	DAFWLGGTFK	Val (90%)
**NLP_2486**	1	DFWNIGMVHK	Thr (100%)
	2	DAWFLGNVVK	Leu (100%)
	3	DVENAGVVTK	Aph (100%)
	4	DAWFLGNVVK	Leu (100%)
	5	DASTIAAVCK	Tyr (100%)
**NLP_2487**	1	DAWQFGLIDK	Gln (100%)
**NLP_3202**	1	DFWNIGMVHK	Thr (100%)
**NLP_3203**	1	AGRFQG-DK	Ile (40%)
**NLP_4431**	1	DPWATGCIDK	Gln (60%)
	2	DVRHFALLAK	Ser (60%)
**NLP_20015**	1	DAWFLGNVVK	Leu (100%)
**NLP_20017**	1	DAFFLGVTFK	Ile (100%)
**NLP_20018**	1	DAFTIAAVWK	Phe (100%)
**NLP_20031**	1	DLYNLSLIWK	Cys (100%)

References

Argüelles, J. C. (2000). Physiological roles of trehalose in bacteria and yeasts: a comparative analysis. *Archives of Microbiology 174*(4), 217–224.

Balskus, E. P. and C. T. Walsh (2010). The genetic and molecular basis for sunscreen biosynthesis in Cyanobacteria. *Science 329*(5999), 1653–1656.

Barbosa, L. C., A. J. Demuner, E. S. de Alvarenga, A. Oliveira, B. King-Diaz, and B. Lotina-Hennsen (2006). Phytogrowth- and photosynthesis-inhibiting properties of nostoclide analogues. *Pest Management Science 62*(3), 214–222.

Becker, J. E., R. E. Moore, and B. S. Moore (2004). Cloning, sequencing, and biochemical characterization of the nostocyclopeptide biosynthetic gene cluster: molecular basis for imine macrocyclization. *Gene 325*, 35–42.

Bergman, B. and L. Hällbom (1982). *Nostoc* of *Peltigera canina* when lichenized and isolated. *Canadian Journal of Botany 60*(10), 2092–2098.

Bergman, B., A. Rai, and U. Rasmussen (2007). Cyanobaterial associations. In C. Elmerich and W. E. William Edward Newton (Eds.), *Associative and Endophytic Nitrogen-fixing Bacteria and Cyanobacterial Associations*, pp. 257–301. Springer.

Black, T. A., Y. Cai, and C. P. Wolk (1993). Spatial expression and autoregulation of *hetR*, a gene involved in the control of heterocyst development in *Anabaena*. *Molecular Microbiologyicrobiology 9*(1), 77–84.

Boissière, M.-C. (1987). Ultrastructural evidence for polyglucosidic reserves in *Nostoc* cells in *Peltigera* and *Collema* and the effect of thallus hydratation. *Bibliotheca Lichenologica 25*, 109–116.

Bordenstein, S. and W. Reznikoff (2005). Mobile DNA in obligate intracellular bacteria. *Nature Reviews Microbiology 3*(9), 688–699.

Bugg, T. and C. Walsh (1992). Intracellular steps of bacterial-cell wall peptidoglycan biosynthesis – enzymology, antibiotics, and antibiotic-resistance. *Natural Product Reports 9*(3), 199–215.

Campbell, E., F. Wong, and J. Meeks (2003). DNA binding properties of the HrmR protein of *Nostoc punctiforme* responsible for transcriptional regulation of genes involved in the differentiation of hormogonia. *Molecular Microbiology 47*(2), 573–582.

Campbell, E. L., M. F. Cohen, and J. C. Meeks (1997). A polyketide-synthase-like gene is involved in the synthesis of heterocyst glycolipids in *Nostoc punctiforme* strain ATCC 29133. *Archives of Microbiology 167*(4), 251–258.

Chandler, M. and O. Fayet (1993). Translational frameshifting in the control of transposition in bacteria. *Molecular Microbiology 7*(4), 497–503.

Chen, X., K. Schreiber, J. Appel, A. Makowka, B. Fähnrich, M. Roettger, M. R. Hajirezaei, F. D. Sönnichsen, P. Schönheit, W. F. Martin, et al. (2016). The Entner–Doudoroff pathway is an overlooked glycolytic route in cyanobacteria and plants. *Proceedings of the National Academy of Sciences of the United States of America*, 201521916.

Cohen, M. and J. Meeks (1997). A hormogonium regulating locus, *hrmUA*, of the cyanobacterium *Nostoc punctiforme* strain ATCC 29133 and its response to an extract of a symbiotic plant partner *Anthoceros punctatus*. *Molecular Plant-Microbe Interactions 10*(2), 280–289.

Cohen, M. F., Y. Sakihama, Y. C. Takagi, T. Ichiba, and H. Yamasaki (2002). Synergistic effect of deoxyanthocyanins from symbiotic fern *Azolla* spp. on *hrmA* gene induction in the cyanobacterium *Nostoc punctiforme*. *Molecular Plant-Microbe Interactions 15*(9), 875–882.

Cohen, M. F. and H. Yamasaki (2000). Flavonoid-induced expression of a symbiosis-related gene in the cyanobacterium *Nostoc punctiforme*. *Journal of Bacteriology 182*(16), 4644–4646.

Crona, M., A. Hofer, J. Astorga-Wells, B.-M. Sjöberg, and F. Tholander (2015). Biochemical characterization of the split class ii ribonucleotide reductase from *Pseudomonas aeruginosa*. *PLOS One 10*(7), e0134293.

Cundliffe, E. (1992). Self-protection mechanisms in antibiotic producers. In D. J. Chadwick and J. Whelan (Eds.), *Secondary metabolites: their function and evolution*, Volume 171, pp. 199–214. Wiley Chichester, UK.

da Silva, N. M. V., T. M. Pereira, A. F. Filho, and T. Matsuura (2011). Taxonomic characterization and antimicrobial activity of actinomycetes associated with foliose lichens from the amazonian ecosystems. *Australian Journal of Basic and Applied Sciences 5*, 910–918.

Daiyasu, H., K. Kuma, T. Yokoi, H. Morii, Y. Koga, and T. H (2005). A study of archaeal enzymes involved in polar lipid synthesis linking amino acid sequence information, genomic contexts and lipid composition. *Archaea 1*(6), 399–410.

Dal Grande, F., I. Widmer, H. H. Wagner, and C. Scheidegger (2012). Vertical and horizontal photobiont transmission within populations of a lichen symbiosis. *Molecular Ecology 21*(13), 3159–3172.

Dao, T., H. Linthorst, and R. Verpoorte (2011). Chalcone synthase and its functions in plant resistance. *Phytochemistry Reviews 10*(3), 397–412.

Darling, A., B. Mau, F. Blattner, and N. Perna (2004). Mauve: Multiple alignment of conserved genomic sequence with rearrangements. *Genome Research 14*(7), 1394–1403.

Díaz, E.-M., M. Sacristán, M.-E. Legaz, and C. Vicente (2009). Isolation and characterization of a cyanobacterium-binding protein and its cell wall receptor in the lichen *Peltigera canina*. *Plant Signaling & Behavior 4*(7), 598–603.

Dijksterhuis, J., K. G. van Driel, M. G. Sanders, D. Molenaar, J. A. Houbraken, R. A. Samson, and E. P. Kets (2002). Trehalose degradation and glucose efflux precede cell ejection during germination of heat-resistant ascospores of *Talaromyces macrosporus*. *Archives of Microbiology 178*(1), 1–7.

Dittmann, E., M. Gugger, K. Sivonen, and D. P. Fewer (2015). Natural product biosynthetic diversity and comparative genomics of the cyanobacteria. *Trends in Microbiology 23*(10), 642–652.

Doehlemann, G., P. Berndt, and M. Hahn (2006). Trehalose metabolism is important for heat stress tolerance and spore germination of *Botrytis cinerea*. *Microbiology 152*(9), 2625–2634.

Dwivedi, B., B. Xue, D. Lundin, R. A. Edwards, and M. Breitbart (2013). A bioinformatic analysis of ribonucleotide reductase genes in phage genomes and metagenomes. *BMC Evolutionary Biology 13*(33).

Edgar, R. C. (2004). Muscle: multiple sequence alignment with high accuracy and high throughput. *Nucleic Acids Research 32*(5), 1792–1797.

Ekman, M., S. Picossi, E. L. Campbell, J. C. Meeks, and E. Flores (2013). A *Nostoc punctiforme* sugar transporter necessary to establish a cyanobacterium-plant symbiosis. *Plant Physiology 161*(4), 1984–1992.

Enderlin, C. and J. Meeks (1983). Pure culture and reconstitution of the *Anthoceros-Nostoc* symbiotic association. *Planta 158*(2), 157–165.

Escartin, F., S. Skouloubris, U. Liebl, and H. Myllykallio (2008). Flavin-dependent thymidylate synthase X limits chromosomal DNA replication. *Proceedings of the National Academy of Sciences of the United States of America 105*(29), 9948–9952.

Feoktistov, A., A. Kitashov, and E. Lobakova (2009). The characterization of lectins from the thripartite lichen *Peltigera aphthosa* (L.) Willd. *Moscow University Biological Sciences Bulletin 64*(1), 23–27.

Filee, J., P. Siguier, and M. Chandler (2007). Insertion sequence diversity in Archaea. *Microbiology and Molecular Biology Reviews 71*(1), 121–157.

Frangeul, L., P. Quillardet, A.-M. Castets, J.-F. Humbert, H. C. P. Matthijs, D. Cortez, A. Tolonen, C.-C. Zhang, S. Gribaldo, J.-C. Kehr, Y. Zilliges, N. Ziemert, S. Becker, E. Talla, A. Latifi, A. Billault, A. Lepelletier, E. Dittmann, C. Bouchier, and N. T. de Marsac (2008). Highly plastic genome of *Microcystis aeruginosa* PCC 7806, a ubiquitous toxic freshwater cyanobacterium. *BMC Genomics 9*.

Freiberg, C., R. Fellay, A. Bairoch, W. J. Broughton, A. Rosenthal, and X. Perret (1997). Molecular basis of symbiosis between *Rhizobium* and legumes. *Nature 387*(6631), 394–401.

Gao, Q. and F. Garcia-Pichel (2011). An ATP-grasp ligase involved in the last biosynthetic step of the iminomycosporine shinorine in *Nostoc punctiforme* ATCC 29133. *Journal of Bacteriology 193*(21), 5923–5928.

Ghannoum, M. (2000). Potential role of phospholipases in virulence and fungal pathogenesis. *Clinical Microbiology Reviews 13*(1), 122–143.

Giladi, M., G. Bitan-Banin, M. Mevarech, and R. Ortenberg (2002). Genetic evidence for a novel thymidylate synthase in the halophilic archaeon *Halobacterium salinarum* and in *Campylobacter jejuni*. *FEMS Microbiology Letters 216*(1), 105–109.

Gleason, F. K. (1990). The natural herbicide, cyanobacterin, specifically disrupts thylakoid membrane structure in *Euglena gracilis* strain Z. *FEMS Microbiology Letters 68*(1-2), 77–81.

Gleason, F. K. and C. A. Baxa (1986). Activity of the natural algicide, cyanobacterin, on eukaryotic microorganisms. *FEMS Microbiology Letters 33*(1), 85–88.

Gleason, F. K. and D. E. Case (1986). Activity of the natural algicide, cyanobacterin, on angiosperms. *Plant Physiology 80*(4), 834–837.

Gleason, F. K. and J. L. Paulson (1984). Site of action of the natural algicide, cyanobacterin, in the blue-green alga, *Synechococcus* sp. *Archives of Microbiology 138*(3), 273–277.

Gogarten, J., A. Senejani, O. Zhaxybayeva, L. Olendzenski, and E. Hilario (2002). Inteins: structure, function, and evolution. *Annual Review of Microbiology 56*, 263–287.

Golakoti, T., W. Y. Yoshida, S. Chaganty, and R. E. Moore (2001). Isolation and structure determination of nostocyclopeptides A1 and A2 from the terrestrial cyanobacterium *Nostoc* sp. ATCC53789. *Journal of Natural Products 64*(1), 54–59.

Golecki, J. (1988). Analysis of the structure and development of bacterial membranes (outer, cytoplasmic and intracytoplasmic membranes). *Methods In Microbiology 20*, 61–77.

González, I., A. Ayuso-Sacido, A. Anderson, and O. Genilloud (2005). Actinomycetes isolated from lichens: evaluation of their diversity and detection of biosynthetic gene sequences. *FEMS Microbiology Ecology 54*(3), 401–415.

Hagemann, M. (2011). Molecular biology of cyanobacterial salt acclimation. *FEMS Microbiology Reviews 35*(1), 87–123.

Hashimoto, M., T. Nonaka, and I. Fujii (2014). Fungal type III polyketide synthases. *Natural Product Reports 31*(10), 1306–1317.

Hausner, G., M. Hafez, and D. R. Edgell (2014). Bacterial group I introns: mobile RNA catalysts. *Mobile DNA 5*(8).

Healy, V., I. Lessard, D. Roper, J. Knox, and C. Walsh (2000). Vancomycin resistance in enterococci: reprogramming of the D-Ala-D-Ala ligases in bacterial peptidoglycan biosynthesis. *Chemistry and Biology 7*(5), R109–R119.

Herdman, M. and R. Rippka (1988). Cellular-differentiation: hormogonia and baeocytes. *Methods In Enzymology 167*, 232–242.

Hoffmann, D., J. M. Hevel, R. E. Moore, and B. S. Moore (2003). Sequence analysis and biochemical characterization of the nostopeptolide A biosynthetic gene cluster from *Nostoc* sp. GSV224. *Gene 311*, 171–180.

Hounsa, C.-G., E. V. Brandt, J. Thevelein, S. Hohmann, and B. A. Prior (1998). Role of trehalose in survival of *Saccharomyces cerevisiae* under osmotic stress. *Microbiology 144*(3), 671–680.

Iyer, L. M., S. Abhiman, A. M. Burroughs, and L. Aravind (2009). Amidoligases with ATP-grasp, glutamine synthetase-like and acetyltransferase-like domains: synthesis of novel metabolites and peptide modifications of proteins. *Molecular BioSystems 5*(12), 1636–1660.

Izard, T. and J. Ellis (2000). The crystal structures of chloramphenicol phosphotransferase reveal a novel inactivation mechanism. *The EMBO Journal 19*(11), 2690–2700.

Jayasimhulu, K., S. M. Hunt, E. S. Kaneshiro, Y. Watanabe, and J.-L. Giner (2007). Detection and identification of *Bacteriovorax stolpii* UKi2 sphingophosphonolipid molecular species. *Journal of the American Society for Mass Spectrometry 18*(3), 394–403.

Johansson, C. and B. Bergman (1994). Reconstitution of the symbiosis of *Gunnera manicata* Linden: cyanobacterial specificity. *New Phytologist*, 643–652.

Jurgens, U. and J. Weckesser (1985). The fine structure and chemical composition of the cell wall and sheath layers of cyanobacteria. *Annales de l'Institut Pasteur / Microbiologie 136A*(1), 41–44.

Kaasalainen, U., D. P. Fewer, J. Jokela, M. Wahlsten, K. Sivonen, and J. Rikkinen (2012). Cyanobacteria produce a high variety of hepatotoxic peptides in lichen symbiosis. *Proceedings of the National Academy of Sciences of the United States of America 109*(15), 5886–5891.

Kaasalainen, U., J. Jokela, D. P. Fewer, K. Sivonen, and J. Rikkinen (2009). Microcystin production in the tripartite cyanolichen *Peltigera leucophlebia*. *Molecular Plant-Microbe Interactions 22*(6), 695–702.

Kacan, S. (2007). *Untersuchungen zur Produktion von Cyanobacterin, einem Toxin aus dem Cyanobakterium* Scytonema hofmanni *Ag. (UTEX1581) – anwendungsbezogene und ökologische Aspekte*. Ph. D. thesis, Universität Rostock.

Kampa, A., A. N. Gagunashvili, T. A. M. Gulder, B. I. Morinaka, C. Daolio, M. Godejohann, V. P. W. Miao, J. Piel, and O. S. Andresson (2013). Metagenomic natural product discovery in lichen provides evidence for a family of biosynthetic pathways in diverse symbioses. *Proceedings of the National Academy of Sciences of the United States of America 110*(33), E3129–E3137.

Kaneshiro, E. S., S. A. Hunt, and Y. Watanabe (2008). *Bacteriovorax stolpii* proliferation and predation without sphingophosphonolipids. *Biochemical and Biophysical Research Communications 367*(1), 21–25.

Khamar, H. J., E. K. Breathwaite, C. E. Prasse, E. R. Fraley, C. R. Secor, F. L. Chibane, J. Elhai, and W.-L. Chiu (2010). Multiple roles of soluble sugars in the establishment of *Gunnera-Nostoc* endosymbiosis. *Plant Physiology 154*(3), 1381–1389.

Kleiner, M., J. C. Young, M. Shah, N. C. VerBerkmoes, and N. Dubilier (2013). Metaproteomics reveals abundant transposase expression in mutualistic endosymbionts. *MBIO 4*(3).

Kodani, S., K. Ishida, and M. Murakami (1999). Occurrence and identification of UDP-*N*-acetylmuramyl-pentapeptide from the cyanobacterium *Anabaena cylindrica*. *FEMS Microbiology Letters 176*(2), 321–325.

Koriem, A. and V. Ahmadjian (1986). An ultrastructural-study of lichenized and cultured nostoc photobionts of *Peltigera canina*, *Peltigera rufescens*, and *Peltigera spuria*. *Endocytobiosis and Cell Research 3*(1), 65–78.

Kraft, L., G. A. Sprenger, and Y. Lindqvist (2002). Conformational changes during the catalytic cycle of gluconate kinase as revealed by X-ray crystallography. *Journal of Molecular Biology 318*(4), 1057–1069.

Kudo, F., A. Miyanaga, and T. Eguchi (2014). Biosynthesis of natural products containing β-amino acids. *Natural Product Reports 31*, 1056–1073.

Kurtz, S., A. Phillippy, A. Delcher, M. Smoot, M. Shumway, C. Antonescu, and S. Salzberg (2004). Versatile and open software for comparing large genomes. *Genome Biology 5*(2).

Leduc, D., S. Graziani, G. Lipowski, C. Marchand, P. Le Maréchal, U. Liebl, and H. Myllykallio (2004). Functional evidence for active site location of tetrameric thymidylate synthase X at the interphase of three monomers. *Proceedings of the National Academy of Sciences of the United States of America 101*(19), 7252–7257.

Leduc, D., S. Graziani, L. Meslet-Cladiere, A. Sodolescu, U. Liebl, and H. Myllykallio (2004). Two distinct pathways for thymidylate (dTMP) synthesis in (hyper) thermophilic bacteria and archaea. *Biochemical Society Transactions 32*(2), 231–235.

Lehr, H., G. Fleminger, and M. Galun (1995). Lectin from the lichen *Peltigera membranacea* (Ach.) Nyl.: characterization and function. *Symbiosis 18*(1), 1–13.

Lehr, H., M. Galun, S. Ott, H.-M. Jahns, and G. Fleminger (2000). Cephalodia of the lichen *Peltigera aphthosa* (L.) Willd. Specific recognition of the compatible photobiont. *Symbiosis 29*(4), 357–365.

Liaimer, A., E. J. Helfrich, K. Hinrichs, A. Guljamow, K. Ishida, C. Hertweck, and E. Dittmann (2015). Nostopeptolide plays a governing role during cellular differentiation of the symbiotic cyanobacterium *Nostoc punctiforme*. *Proceedings of the National Academy of Sciences of the United States of America 112*(6), 1862–1867.

Liaimer, A., J. B. Jensen, and E. Dittmann (2016). A genetic and chemical perspective on symbiotic recruitment of cyanobacteria of the genus *Nostoc* into the host plant *Blasia pusilla* L. *Frontiers in Microbiology 7*.

Lin, S., S. Haas, T. Zemojtel, P. Xiao, M. Vingron, and R. Li (2011). Genome-wide comparison of cyanobacterial transposable elements, potential genetic diversity indicators. *Gene 473*(2), 139–149.

Lockhart, C., P. Rowell, and W. Stewart (1978). Phytohaemagglutinins from the nitrogen-fixing lichens *Peltigera canina* and *P. polydactyla*. *FEMS Microbiology Letters 3*(3), 127–130.

Mason, C., K. Edwards, R. Carlson, J. Pignatello, F. Gleason, and J. Wood (1982). Isolation of chlorine-containing antibiotic from the freshwater cyanobacterium *Scytonema hofmanni*. *Science 215*(4531), 400–402.

Matsuhashi, M., M. Furuyama, and B. Maruo (1969). Effect of inhibitors of bacterial cell wall synthesis on the growth of *Anabaena variabilis*, a blue-green alga. *Agricultural and Biological Chemistry 33*(12), 1758–1760.

McIntosh, E. M., M. H. Gadsden, and R. H. Haynes (1986). Transcription of genes encoding enzymes involved in dna synthesis during the cell cycle of *Saccharomyces cerevisiae*. *Molecular and General Genetics 204*(3), 363–366.

McIntosh, E. M. and R. H. Haynes (1984). Isolation of a *Saccharomyces cerevisiae* mutant strain deficient in deoxycytidylate deaminase activity and partial characterization of the enzyme. *Journal of Bacteriology 158*(2), 644–649.

Meeks, J. (1998). Symbiosis between nitrogen-fixing cyanobacteria and plants – The establishment of symbiosis causes dramatic morphological and physiological changes in the cyanobacterium. *BioScience 48*(4), 266–276.

Meeks, J. C. (2003). Symbiotic interactions between *Nostoc punctiforme*, a multicellular cyanobacterium, and the hornwort *Anthoceros punctatus*. *Symbiosis 35*(1-3), 55–71.

Meeks, J. C. (2005). The genome of the filamentous cyanobacterium *Nostoc punctiforme*. what can we learn from it about free-living and symbiotic nitrogen fixation? In R. Palacios and W. E. Newton (Eds.), *Genomes and Genomics of Nitrogen-fixing Organisms*, Volume 3 of *Nitrogen Fixation: Origins, Applications, and Research Progress*, pp. 27–70. Springer.

Meng, Q., Y. Zhang, and X.-Q. Liu (2007). Rare group I intron with insertion sequence element in a bacterial ribonucleotide reductase gene. *Journal of Bacteriology 189*(5), 2150–2154.

Metcalf, W. W. and W. A. van der Donk (2009). Biosynthesis of phosphonic and phosphinic acid natural products. *Annual Review of Biochemistry 78*, 65–94.

Millbank, J. (1977). The oxygen tension within lichen thalli. *New Phytologist 79*(3), 649–657.

Moran, N. and G. Plague (2004). Genomic changes following host restriction in bacteria. *Current Opinion In Genetics and Development 14*(6), 627–633.

Mosher, R. H., D. J. Camp, K. Yang, M. P. Brown, W. V. Shaw, and L. C. Vining (1995). Inactivation of chloramphenicol by *O*-phosphorylation a novel resistance mechanism in *Streptomyces venezuelae* ISP5230, a chloramphenicol producer. *Journal of Biological Chemistry 270*(45), 27000–27006.

Myllykallio, H., G. Lipowski, D. Leduc, J. Filee, P. Forterre, and U. Liebl (2002). An alternative flavin-dependent mechanism for thymidylate synthesis. *Science 297*(5578), 105–107.

Newton, I. L. G. and S. R. Bordenstein (2011). Correlations between cacterial ecology and mobile DNA. *Current Microbiology 62*(1), 198–208.

Nilsson, M., U. Rasmussen, and B. Bergman (2006). Cyanobacterial chemotaxis to extracts of host and nonhost plants. *FEMS Microbiology Ecology 55*(3), 382–390.

Ow, S. Y., J. Noirel, T. Cardona, A. Taton, P. Lindblad, K. Stensjö, and P. C. Wright (2008). Quantitative overview of N_2 fixation in *Nostoc punctiforme* ATCC 29133 through cellular enrichments and iTRAQ shotgun proteomics. *Journal of Proteome Research 8*(1), 187–198.

Paquin, B., S. D. Kathe, S. A. Nierzwicki-Bauer, and D. A. Shub (1997). Origin and evolution of group I introns in cyanobacterial tRNA genes. *Journal of Bacteriology 179*(21), 6798–6806.

Park, J. (1952). Uridine-5'-pyrophosphate derivatives. *Journal of Biological Chemistry 194*(2), 877–904.

Parks, D. H., M. Imelfort, C. T. Skennerton, P. Hugenholtz, and G. W. Tyson (2015). Checkm: assessing the quality of microbial genomes recovered from isolates, single cells, and metagenomes. *Genome Research 25*(7), 1043–1055.

Paulsrud, P., J. Rikkinen, and P. Lindblad (1998). Cyanobiont specificity in some *Nostoc*-containing lichens and in a *Peltigera aphthosa* photosymbiodeme. *New Phytologist 139*(3), 517–524.

Paulsrud, P., J. Rikkinen, and P. Lindblad (2001). Field investigations on cyanobacterial specificity in *Peltigera aphthosa*. *New Phytologist 152*(1), 117–123.

Pearce, J. and N. Carr (1969). The incorporation and metabolism of glucose by *Anabaena variabilis*. *Journal of General Microbiology 54*(3), 451–462.

Pearson, L. A., E. Dittmann, R. Mazmouz, S. E. Ongley, P. M. D'Agostino, and B. A. Neilan (2016). The genetics, biosynthesis and regulation of toxic specialized metabolites of cyanobacteria. *Harmful Algae 54*, 98–111.

Petit, P. (1982). Phytolectins from the nitrogen-fixing lichen *Peltigera horizontalis*: the binding pattern of primary protein extract. *New Phytologist 91*(4), 705–710.

Petit, P., R. Lallemant, and D. Savoye (1983). Purified phytolectin from the lichen *Peltigera canina* var. *canina* which binds to the phycobiont cell walls and its use as cytochemical marker in situ. *New Phytologist 94*(1), 103–110.

Picossi, S., E. Flores, and M. Ekman (2013). Diverse roles of the glcp glucose permease in free-living and symbiotic cyanobacteria. *Plant Signaling and Behavior 8*(12), e27416.

Pignatello, J. J., J. Porwoll, R. E. Carlson, A. Xavier, F. K. Gleason, and J. M. Wood (1983). Structure of the antibiotic cyanobacterin, a chlorine-containing γ-lactone from the freshwater cyanobacterium *Scytonema hofmanni*. *The Journal of Organic Chemistry 48*(22), 4035–4038.

Ran, L., J. Larsson, T. Vigil-Stenman, J. A. A. Nylander, K. Ininbergs, W.-W. Zheng, A. Lapidus, S. Lowry, R. Haselkorn, and B. Bergman (2010). Genome erosion in a nitrogen-fixing vertically transmitted endosymbiotic multicellular cyanobacterium. *PLOS One 5*(7).

Rasmussen, U., C. Johansson, A. Renglin, C. Petersson, and B. Bergman (1996). A molecular characterization of the *Gunnera-Nostoc* symbiosis: comparison with *Rhizobium-* and *Agrobacterium*-plant interactions. *New Phytologist 133*(3), 391–398.

Robert, X. and P. Gouet (2014). Deciphering key features in protein structures with the new endscript server. *Nucleic Acids Research 42*(W1), W320–W324.

Röttig, M., M. H. Medema, K. Blin, T. Weber, C. Rausch, and O. Kohlbacher (2011). NRPSpredictor2—a web server for predicting NRPS adenylation domain specificity. *Nucleic Acids Research 39*, W362–W367.

Rounge, T. B., T. Rohrlack, T. Kristensen, and K. S. Jakobsen (2008). Recombination and selectional forces in cyanopeptolin NRPS operons from highly similar, but geographically remote *Planktothrix* strains. *BMC Microbiology 8*(141).

Saeng-in, P., N. Kuncharoen, N. Chamroensaksri, and S. Tanasupawat (2015). Identification and antimicrobial activity of *Streptomyces* and *Actinoplanes* strains from lichens. *Journal of Applied Pharmaceutical Science 5*(1), 023–029.

Scheurwater, E., C. W. Reid, and A. J. Clarke (2008). Lytic transglycosylases: bacterial space-making autolysins. *International Journal of Biochemistry and Cell Biology 40*(4), 586–591.

Schmitz-Esser, S., T. Penz, A. Spang, and M. Horn (2011). A bacterial genome in transition – an exceptional enrichment of IS elements but lack of evidence for recent transposition in the symbiont *Amoebophilus asiaticus*. *BMC Evolutionary Biology 11*.

Seigler, D. S. (2012). *Plant secondary metabolism*. Springer Science & Business Media.

Seshime, Y., P. R. Juvvadi, I. Fujii, and K. Kitamoto (2005). Discovery of a novel superfamily of type III polyketide synthases in *Aspergillus oryzae*. *Biochemical and Biophysical Research Communications 331*(1), 253–260.

Sievers, F., A. Wilm, D. Dineen, T. J. Gibson, K. Karplus, W. Li, R. Lopez, H. McWilliam, M. Remmert, J. Söding, J. D. Thompson, and D. G. Higgins (2011). Fast, scalable generation of high-quality protein multiple sequence alignments using Clustal Omega. *Molecular Systems Biology 7*(1), 539.

Sigurbjörnsdóttir, M., Ó. Andrésson, and O. Vilhelmsson (2015). Analysis of the *Peltigera membranacea* metagenome indicates that lichen-associated bacteria are involved in phosphate solubilization. *Microbiology 161*(5), 989–996.

Singh, R. S. and A. K. Walia (2014). Characteristics of lichen lectins and their role in symbiosis. *Symbiosis 62*(3), 123–134.

Spiteller, P. and F. von Nussbaum (2005). β-amino acids in natural products. In E. Juaristi and V. Soloshonok (Eds.), *Enantioselective Synthesis Of β-Amino Acids* (Second ed.)., pp. 19–92. John Wiley & Sons, Inc.

Svanström, Å. and P. Melin (2013). Intracellular trehalase activity is required for development, germination and heat-stress resistance of *Aspergillus niger* conidia. *Research in Microbiology 164*(2), 91–99.

Tang, L., I. Watanabe, and C. Liu (1990). Limited multiplication of symbiotic cyanobacteria of *Azolla* spp. on artificial media. *Applied and Environmental Microbiology 56*(11), 3623–3626.

Torrents, E. (2014). Ribonucleotide reductases: essential enzymes for bacterial life. *Frontiers in Cellular and Infection Microbiology 4*.

Tschermak-Woess, E. (1988). The algal partner. In M. Galun (Ed.), *CRC Handbook of Lichenology*, Volume I, pp. 39–92. CRC Press Inc., Boca Raton.

Vining, L. and C. Stuttard (1995). Chloramphenicol. *Biotechnology 28*, 505–530.

Vining, L. and D. Westlake (1984). Chloramphenicol: properties, biosynthesis, and fermentation. In E. Vandamme (Ed.), *Biotechnology of Industrial Antibiotics*, pp. 387–409. Marcel Dekker, New York, NY.

von Nussbaum, F. and P. Spiteller (2006). β-amino acids in nature. In C. Schmuck and H. Wennemers (Eds.), *Highlights In Bioorganic Chemistry: Methods And Applications*, pp. 90–106. John Wiley & Sons, Inc.

Watanabe, Y., M. Nakajima, T. Hoshino, K. Jayasimhulu, E. Brooks, and E. Kaneshiro (2001). A novel sphingophosphonolipid head group 1-hydroxy-2-aminoethyl phosphonate in *Bdellovibrio stolpii*. *Lipids 36*(5), 513–519.

Waterhouse, A. M., J. B. Procter, D. M. A. Martin, M. Clamp, and G. J. Barton (2009). Jalview Version 2 – a multiple sequence alignment editor and analysis workbench. *Bioinformatics 25*(9), 1189–1191.

Wu, H., Z. Hu, and X. Liu (1998). Protein trans-splicing by a split intein encoded in a split DnaE gene of *Synechocystis* sp. PCC6803. *Proceedings of the National Academy of Sciences of the United States of America 95*(16), 9226–9231.

Yang, X., Y. Shimizu, J. R. Steiner, and J. Clardy (1993). Nostoclide I and II, extracellular metabolites from a symbiotic cyanobacterium, *Nostoc* sp., from the lichen *Peltigera canina*. *Tetrahedron Letters 34*(5), 761–764.

Yu, X., J. R. Doroghazi, S. C. Janga, J. K. Zhang, B. Circello, B. M. Griffin, D. P. Labeda, and W. W. Metcalf (2013). Diversity and abundance of phosphonate biosynthetic genes in nature. *Proceedings of the National Academy of Sciences of the United States of America 110*(51), 20759–20764.

Zhu, T., S. Hou, X. Lu, and W. R. Hess (2017). Draft genome sequences of nine cyanobacterial strains from diverse habitats. *Genome Announcements 5*(9), e01676–16.
